# Global, regional, and national economic burden of hematologic malignancies (1990–2021) with projections to 2050

**DOI:** 10.3389/fpubh.2025.1570792

**Published:** 2025-06-27

**Authors:** Zhexian Li, Guangpeng Chen, Guibin Du

**Affiliations:** ^1^Department of Geriatrics, Hebei PetroChina Central Hospital, Langfang, Hebei, China; ^2^School of Public Health, Fudan University, Shanghai, China; ^3^Department of Ultrasound Medicine, Hebei PetroChina Central Hospital, Langfang, Hebei, China

**Keywords:** Global Burden of Disease, hematologic malignancies, economic burden, Hodgkin lymphoma, leukemia, multiple myeloma, non-Hodgkin lymphoma

## Abstract

**Background:**

Hematologic malignancies (HM) impose substantial healthcare and productivity-related costs globally. However, disparities in economic impact across regions and countries remain insufficiently explored. This study aimed to evaluate the global, regional, and national economic burden of HM and its subtypes (leukemia, non-Hodgkin lymphoma, multiple myeloma, and Hodgkin lymphoma) from 1990 to 2021, with projections to 2050.

**Methods:**

Data from the Global Burden of Disease 2021 study were utilized to estimate the economic burden of HM using the value of a statistical life year (VSLY) approach, based on disability-adjusted life years (DALYs). Decomposition analysis was conducted to identify drivers of economic burden, including population growth and aging. Future trends were modeled using the Bayesian Age-Period-Cohort (BAPC) model, and comparisons of economic burden were made across countries by income levels.

**Results:**

In 2021, the global economic burden of HM reached $1.516 trillion, a 52.8% increase from $992 billion in 1990. This represented approximately 1% of the global GDP, with high-income countries (HICs) bearing the largest share of 2.17% of GDP, compared to 0.58% in lower-middle-income countries (LMICs). The United States had the highest national burden at $417.42 billion (95% UI: $389.49–$435.80 billion), followed by China $133.84 billion (95% UI: $98.63–$166.21 billion), $113.03 billion (95% UI: $101.62–$122.88 billion), and Japan $88.30 billion (95% UI: $85.65–$90.24 billion). By 2050, the global burden is projected to decline to $1.249 trillion, driven by healthcare advancements in HICs, but with a rising burden in upper-middle-income countries (UMICs), which are expected to account for 48.1% of the global burden. China is projected to lead globally with $421.65 billion (95% UI: $314.68–$1,495.35 billion), followed by India ($123.8 billion), while the United States is expected to decline to $101.6 billion. Subtype-specific analysis revealed that Leukemia accounted for the largest proportion of the burden in 2021 ($651 billion, 42.9%), followed by NHL ($492 billion, 32.5%), multiple myeloma ($278.17 billion, 100.5%), and Hodgkin lymphoma ($43.84 billion, 21%).

**Conclusions:**

The economic burden of HM has increased significantly, with marked disparities across regions and income levels. By 2050, the burden is expected to shift from high- to middle-income countries. Investments in early diagnosis, affordable treatments, and healthcare improvements are essential to reduce the burden and address inequities.

## Introduction

Hematologic malignancies (HM) encompass a diverse group of blood cancers that impose substantial healthcare and productivity-related costs on global healthcare systems due to their prolonged and costly treatments, as well as productivity losses from morbidity and premature mortality (1, 2). The four major types of HMs—leukemia, Hodgkin lymphoma (HL), non-Hodgkin lymphoma (NHL), and multiple myeloma (MM)—each contribute to this burden to varying extents. For instance, in 2020, leukemia alone accounted for an estimated 474,519 new cases and 311,594 deaths worldwide, demonstrating the substantial healthcare costs associated with this disease (3). According to a recent study by Chen et al. based on GBD data, the cumulative global economic cost of cancer from 2020 to 2050 is projected to reach $25.2 trillion in international dollars (at constant 2017 prices). Among all cancer types, leukemia ranks fifth, with an estimated 6.3% share of the total cost, equivalent to approximately $1.59 trillion over the 30-year period (4). Similarly, NHL and MM add to the global economic impact, primarily driven by treatment expenses and disability-adjusted life years (DALYs) lost annually (5). Lower-middle-income countries (LMICs), in particular, face dual challenges: constrained healthcare resources and the substantial costs associated with managing these cancers, both of which exacerbate their overall economic burden (6).

Recent advancements in medical technology, such as targeted therapies, immunotherapies, and precision medicine, have significantly improved the prognosis and survival of HM patients, particularly in high-income countries (7). However, these innovations come with high costs, exacerbating inequalities in access across regions. In LMICs, restricted availability of advanced therapies and underdeveloped healthcare infrastructure limit the benefits of these breakthroughs, resulting in disparities in economic burden and patient outcomes (8). Furthermore, the high costs of novel treatments, coupled with the ongoing need for supportive care, have placed increasing financial pressure on both patients and healthcare systems worldwide. These challenges underline the importance of evaluating the global economic burden of HM to inform resource allocation, improve cost-effectiveness, and reduce disparities across regions (9).

To effectively address these issues, it is essential to adopt a long-term perspective that extends to 2050. The global population is aging, and hematologic malignancies are more prevalent among older adults, suggesting a substantial rise in disease burden in the coming decades. Long-term projections are therefore necessary to anticipate future healthcare demands, guide sustainable resource planning, and inform policies that strengthen health system resilience. Moreover, such projections align with the Sustainable Development Goals (SDGs), particularly those targeting health equity, universal health coverage, and the reduction of non-communicable disease burden by 2030 and beyond. By incorporating demographic shifts and the expected rise in incidence, long-range forecasting provides critical insights for ensuring equitable and effective responses across diverse healthcare settings.

## Methods

### Data sources and analysis

This study used data from the Global Burden of Disease (GBD) 2021 Study, which provides comprehensive estimates of incidence, mortality, and DALYs for diseases across 204 countries and territories (10, 11). We focused on the economic burden of HM and their four subtypes (leukemia, NHL, MM, and HL) from 1990 to 2021, with projections to 2050.

Economic burden was estimated using the value of a statistical life year (VSLY) method, which measures the economic value of lost health due to DALYs. This approach accounts for differences in socio-economic conditions across countries and regions. All monetary values were adjusted to 2021 international dollars and discounted at an annual rate of 3% (12).

DALYs for HM and its subtypes were extracted from the GBD 2021 database, which provides age-standardized estimates for every country and region. The GBD study applies a standardized modeling framework that integrates a wide range of data sources, including vital registration systems, cancer registries, household surveys, and verbal autopsy data. In countries where data are limited or of lower quality—particularly in low- and middle-income settings—the GBD employs statistical “borrowing of strength” across time, age groups, and geographic regions using Bayesian meta-regression techniques (DisMod-MR 2.1) to generate internally consistent estimates of disease burden. These DALY figures were then converted into monetary costs using region-specific VSLY data to reflect the economic consequences of health losses. To analyze historical trends and project future economic burden, population data were sourced from the United Nations World Population Prospects, incorporating demographic factors such as population growth, aging, and economic development (13, 14).

Future trends in the economic burden were projected using the Bayesian Age-Period-Cohort (BAPC) model, an extension of the classical age-period-cohort (APC) framework (14). The APC model assumes that incidence or mortality rates are influenced by age structure, calendar time (period), and birth cohort. Mathematically, it is formulated as a log-linear Poisson model of the form:


$$\begin{array}{l} \text{nij}=\text{log}\left(\text{λij}\right)=\text{μ}+\text{αi}+\text{βj}+\text{γk}+\text{ε} \end{array}$$

Where μ represents the intercept, ε is the random error, αi, βj, γk denote the effects of age, period, and cohort, respectively. To improve the flexibility and predictive power of the model, the Bayesian extension (BAPC) applies second-order random walk (RW2) priors to the age, period, and cohort effects, allowing for smooth trends across adjacent time points. The variances of these priors are modeled using inverse gamma distributions. Bayesian inference is implemented using the Integrated Nested Laplace Approximation (INLA) algorithm, which provides greater computational efficiency and accuracy than traditional Markov Chain Monte Carlo (MCMC) methods (14, 15). This approach allowed us to incorporate demographic changes, such as population aging, cohort-specific risks, and economic growth, thereby enhancing the robustness of the long-term projections. For forecasting purposes, we defined three reference scenarios: a baseline scenario assuming incidence and mortality rates remained constant at 2021 levels; a negative trend scenario assuming a 1% annual increase; and a positive trend scenario assuming a 1% annual decrease from 2021 onward. To explore disparities, countries were grouped into high-income (HICs), upper-middle-income (UMICs), lower-middle-income (LMICs), and low-income (LICs) groups based on the World Bank 2021 income classification.

To assess the robustness of our economic burden estimates, we conducted an uncertainty analysis following the approach proposed by Chen et al. (4). Specifically, the VSLY was artificially varied by ±20% to reflect reasonable fluctuations in economic valuation. For DALYs, we used the corresponding lower and upper bounds of the GBD-provided uncertainty intervals. We assumed a normal distribution of DALYs within each country's uncertainty range and generated 1,000 bootstrap samples. These simulated samples were then used to derive the 95% confidence intervals of the economic burden estimates for each World Bank income group.

To identify the drivers of changes in health-related costs from 1990 to 2021, we employed a cost estimation approach based on the VSLY and DALYs. Given that both VSLY and DALYs changed over time, we applied the Shapley decomposition method to attribute the total cost variation to each factor. This game-theoretic approach allows for fair and consistent attribution of marginal contributions, with desirable properties such as additivity and order independence. Decomposition was conducted at the country level and aggregated globally to ensure robust and interpretable results (16).

Additionally, All analyses in this study were conducted using R version 4.2.1. The main analytical packages included dplyr, BAPC, INLA, and easyGBDR. Data visualization was performed using ggplot2, patchwork, and ggsci, among others.

### US and global estimates of the value of a statistical life

To estimate the value of a statistical life (VSL) in the US and globally, we utilized estimates derived from a 2018 meta-analysis of 1,025 VSL values across 68 studies, which concluded that the median VSL ranged between $9.7 million and $10.1 million (2015 US dollars) depending on the methodology employed (17). This analysis specifically focused on studies using data from the US Census of Fatal Occupational Injuries (CFOI), known for providing accurate estimates of occupational mortality risks. After accounting for publication bias, the CFOI-based VSL was approximately $10 million (2015 US dollars). The 2015 estimate of $9.4 million (in 2015 US dollars) for the VSL, as provided by the US Department of Health and Human Services (HHS), was adjusted for inflation to $11.23 million in 2021 US dollars using the Consumer Price Index (CPI) (18). Globally, the VSL was estimated using a methodology recommended by an international panel of benefit-cost experts (19, 20), which adjusts for differences in income levels across countries by incorporating the gross national income per capita (GNIPC). This adjustment was performed using the formula below:


$$\begin{array}{l} VSL_{x}=VSL_{USA}\times {\left[\frac{GNIPC_{x}}{GNIPC_{USA}}\right]}^{E} \end{array}$$

Here, VSLx represents the VSL of country x, VSL_USA_ is the US VSL, GNIPC denotes the gross national income per capita (PPP) for either country x or the US (2019 values), and E is the income elasticity of VSL. We used elasticity values of 1 for HICs and 1.5 for LMICs, consistent with prior meta-analyses (12). GNIPC data were sourced from the World Bank (21), and a minimum VSL of 20 times the GNI per capita was applied in cases where estimates appeared too low (12). Country income classifications followed the World Bank's definitions for 1990 and 2021, with adjustments for future projections (22).

### Economic valuation of DALYs using the value of a statistical life year

To estimate the economic impact of a year of life lost to HM, we used the VSLY. The VSLY is derived by distributing the total VSL across the remaining life years of a population targeted by a policy or intervention aimed at reducing mortality risk. For example, when analyzing interventions that reduce neonatal mortality, life expectancy at birth is used to estimate the VSLY. For policies targeting adults or working-age individuals, the median age and the life expectancy at that age are recommended (23). We obtained data on median age and life expectancy from the World Population Prospects and distributed the value of a statistical life (VSL) evenly across the remaining life years, without applying any discounting (21, 22).

Future VSLY values were projected by adjusting the 2021 VSLY based on income growth. Using World Bank data, we calculated the average annual growth rate of GNI per capita (PPP, current international dollars) from 2011 to 2020, and projected VSLY values for 2030, 2040, and 2050 based on this growth rate (12, 21).

Each DALY lost was equated to one VSLY lost, following standard economic evaluation methods. The economic burden of HM was calculated by summing all VSLYs lost, with monetary values expressed in 2021 international dollars. Adjustments were made using the CPI for values before 2021, and a 3% discount rate was applied for values after 2021 (12).

### Regional and income-level disparities

To explore disparities, economic burden estimates were compared across income levels—HICs, UMICs, LMICs, and LICs—and geographic regions. This stratification allowed us to capture variations in economic burden due to differences in socio-economic conditions, mortality risks, and access to healthcare.

## Results

### Global and regional trends in the economic burden of HM

**Figure 1:**
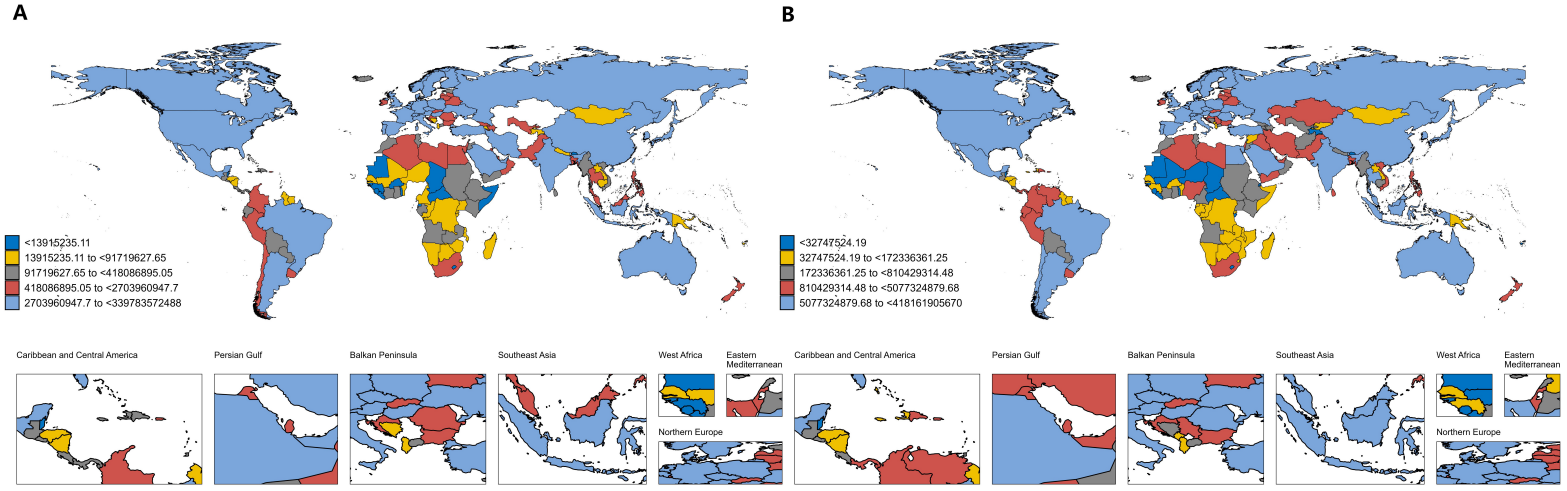
Global economic burden of hematologic malignancies; **(A)** economic burden in 1990; **(B)** economic burden in 2021. This figure shows the estimated total economic burden attributable to hematologic malignancies by country in 1990 **(A)** and 2021 **(B)**, with countries grouped into quintiles based on total burden for each year. Each country is color-coded accordingly: dark blue represents the lowest quintile (< $1.39 billion in 1990; < $32.7 million in 2021), yellow the second quintile ($1.39–9.17 billion in 1990; $32.7–172.3 million in 2021), gray the third quintile ($9.17–41.8 billion in 1990; $172.3–810.4 million in 2021), red the fourth quintile ($41.8–270.4 billion in 1990; $810.4 million–$5.08 billion in 2021), and light blue the highest quintile ($270.4 billion–$3.40 trillion in 1990; $5.08–$41.82 billion in 2021).

**Figure 2:**
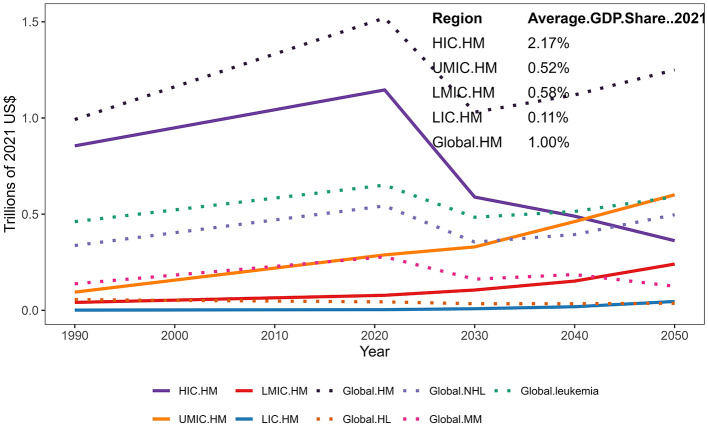
Estimated economic burden by country groups from 1990 to 2050. Projected healthcare expenditures related to hematological malignancies (HM) across income groups and globally from 1990 to 2050. Solid lines represent expenditures in High-Income Countries (HIC), Upper-Middle-Income Countries (UMIC), Lower-Middle-Income Countries (LMIC), and Low-Income Countries (LIC). Dotted lines indicate global expenditures, including total HM (Global.HM), Hodgkin Lymphoma (HL), Non-Hodgkin Lymphoma (NHL), Multiple Myeloma (MM), and leukemia. All values are expressed in trillions of 2021 US dollars. The inset table shows the average GDP share of HM-related healthcare spending in 2021 for each income group and globally.

As shown in Figures 1, 2, the global economic burden of HM increased from $992 billion (95%CI) in 1990 to $1.516 trillion in 2021, representing a 52.8% increase. Projections suggest that this burden will slightly decline to $1.055 trillion by 2030, before rising again to $1.169 trillion by 2040 and $1.249 trillion by 2050 (Figure 2). These trends likely reflect advancements in healthcare systems, improved access to treatment, and evolving economic dynamics across different income groups.

HICs consistently accounted for the majority of the global HM burden, contributing 86.2% ($855 billion) in 1990 and 75.6% ($1.146 trillion) in 2021 (Figure 2). However, their share is projected to decline significantly, reaching 52.69% ($589 billion) by 2030, 43.07% ($489 billion) by 2040, and 29.81% ($410 billion) by 2050, as the burden shifts to UMICs. By 2050, UMICs are expected to bear 47.79% ($657 billion) of the global burden, surpassing HICs and becoming the largest contributors. LMICs and LICs will also see rising burdens, reaching $260 billion (18.94%) and $47 billion (3.47%), respectively, by 2050. These trends highlight disparities in healthcare resource allocation and economic strain across regions. Detailed results and uncertainty intervals are provided in Supplementary Table S6.

**Figure 3:**
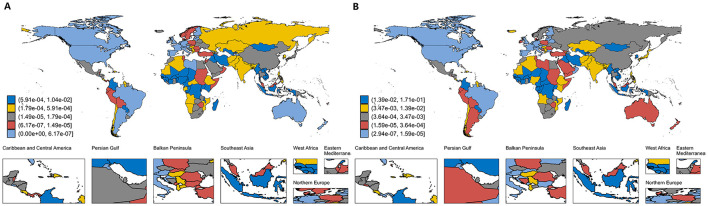
Proportion of global economic burden of hematologic malignancies relative to GDP; **(A)** economic burden in 1990; **(B)** economic burden in 2021. This figure presents country-level estimates of the economic burden attributable to hematologic malignancies as a share of national GDP in 1990 and 2021, grouped into quintiles based on relative burden. Each country is color-coded from highest to lowest burden as follows: dark blue (5.91 × 10^−4^−1.04 × 10^−2^ in 1990; 1.39 × 10^−2^−1.71 × 10^−1^ in 2021), yellow (1.79 × 10^−4^−5.91 × 10^−4^ in 1990; 3.47 × 10^−3^−1.39 × 10^−2^ in 2021), gray (1.49 × 10^−5^−1.79 × 10^−4^ in 1990; 3.64 × 10^−4^−3.47 × 10^−3^ in 2021), red (6.17 × 10^−7^−1.49 × 10^−5^ in 1990; 1.59 × 10^−5^−3.64 × 10^−4^ in 2021), and light blue (0–6.17 × 10^−7^ in 1990; 2.94 × 10^−7^−1.59 × 10^−5^ in 2021). Quintile thresholds are defined separately for each year.

In 1990, the economic burden of HM as a percentage of GDP was 2.48% in HICs, compared to 0.52% in LMICs, and 0.15% and 0.12% in LICs and UMICs, respectively. The global average economic burden accounted for 0.97% of the total GDP in 1990, increasing slightly to 1.00% by 2021. HICs continued to bear the highest proportion at 2.17% in 2021, reflecting substantial investments in healthcare systems. In contrast, LMICs and LICs reported considerably lower shares of 0.58% and 0.11%, respectively. Notably, several smaller economies experienced disproportionately high economic burdens from HM, with the highest GDP shares observed in Tajikistan (23.8%), Puerto Rico (17.1%), and Croatia (5.8%) in 2021 (Figure 3). These findings underscore the vulnerability of smaller economies where healthcare costs often far exceed national budgets. More details on the proportion of HM economic burden attributed to countries of different income levels can be found in Supplementary Table S7.

### Country-level disparities

As detailed in Figure 1 and Supplementary Table S1, the United States consistently incurred the highest national economic burden of HM, with costs increasing from $339.84 billion (95% UI: $327.85–$348.71 billion) in 1990 to $417.42 billion (95% UI: $389.49–$435.80 billion) in 2021. This substantial burden was primarily driven by the high prevalence of HM, widespread access to advanced therapies, and elevated treatment costs. In 1990, Japan and Germany ranked as the second and third highest contributors, with economic burdens of $88.30 billion (95% UI: $85.65–$90.24 billion) and $82.14 billion (95% UI: $77.61–$87.27 billion), respectively. However, by 2021, China had emerged as the second-largest contributor, with an economic burden of $133.84 billion (95% UI: $98.63–$166.21 billion), surpassing Germany, which reported a burden of $113.03 billion (95% UI: $101.62–$122.88 billion).

**Figure 4:**
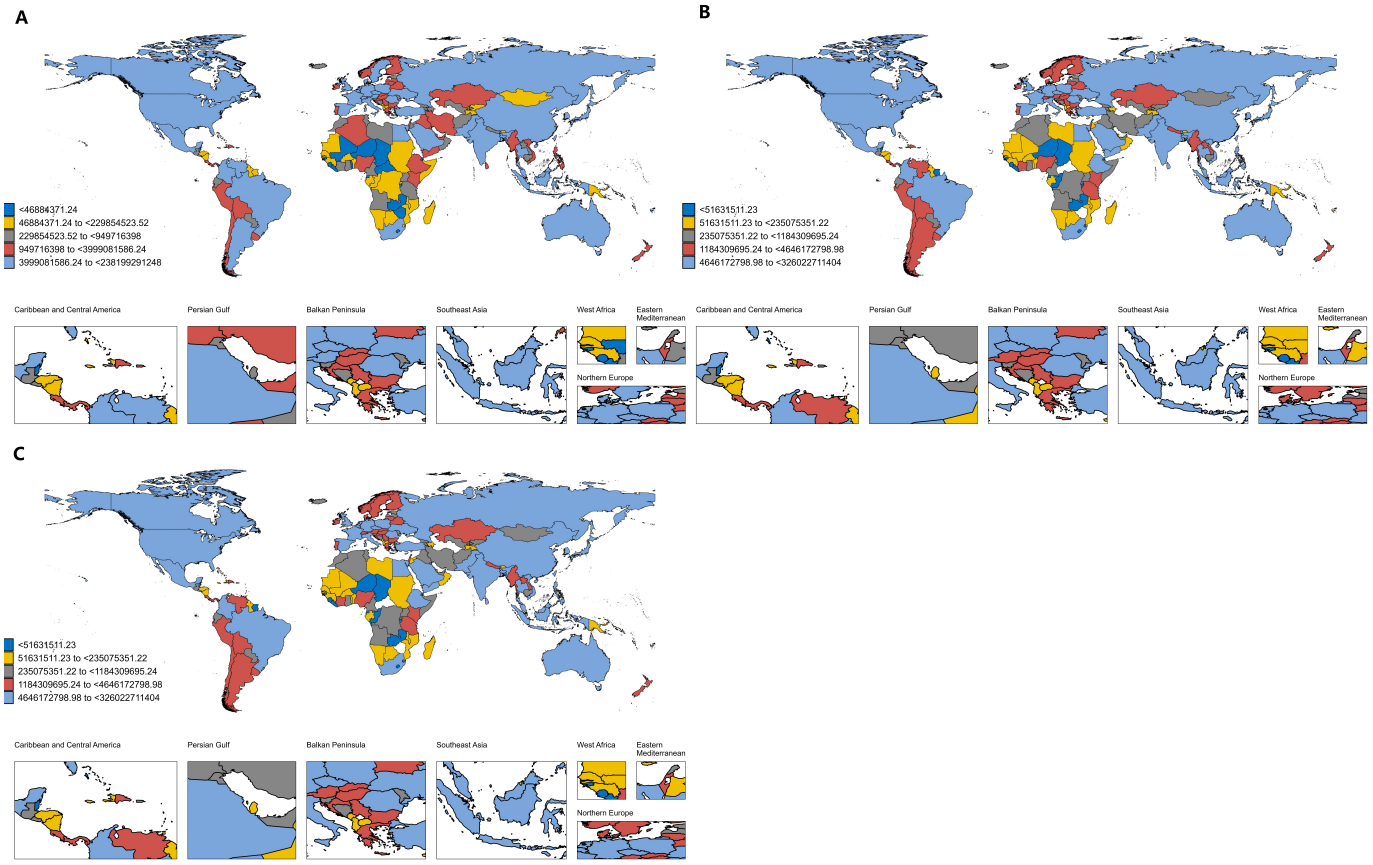
Predicted global economic burden of hematologic malignancies; **(A)** economic burden in 2030; **(B)** economic burden in 2040; **(C)** economic burden in 2050. This figure presents country-level projections of the total economic burden attributable to hematologic malignancies in 2030 **(A)**, 2040 **(B)**, and 2050 **(C)**, grouped into quintiles based on projected absolute burden. Each country is color-coded accordingly: dark blue represents the lowest quintile (< $469 million in 2030; < $516 million in 2040 and 2050), yellow the second quintile ($469–2.30 billion in 2030; $516–2.36 billion in 2040 and 2050), gray the third quintile ($2.30–5.00 billion in 2030; $2.36–11.84 billion in 2040 and 2050), red the fourth quintile ($5.00–10.00 billion in 2030; $11.84–46.46 billion in 2040 and 2050), and light blue the highest quintile ($10.00–23.82 billion in 2030; $46.46–326.02 billion in 2040 and 2050). The color scale is based on absolute predicted values and differs across years. Countries with missing or unavailable data are shown in white.

Projections reveal significant shifts in the global distribution of HM costs. As shown in Figure 4, By 2030, the United States is projected to incur the highest national economic burden from hematological malignancies, with costs estimated at $238.20 billion (95% UI: $164.98–$304.25 billion), followed by China at $175.90 billion (95% UI: $114.99–$249.42 billion) and Germany at $51.41 billion (95% UI: $39.75–$96.90 billion). By 2040, China is expected to surpass the United States, with projected costs reaching $275.04 billion (95% UI: $204.80–$621.94 billion), while the United States and India are estimated to bear $208.69 billion (95% UI: $146.08–$370.01 billion) and $55.96 billion (95% UI: $40.85–$97.39 billion), respectively. By 2050, the economic burden in China is anticipated to rise substantially to $421.65 billion (95% UI: $314.68–$1,495.35 billion), driven by rapid population aging and increasing healthcare expenditures, surpassing that of the United States ($183.12 billion, 95% UI: $128.18–$443.03 billion) and India ($81.95 billion, 95% UI: $59.82–$191.62 billion). These trends underscore growing economic pressures on rapidly developing economies (see Supplementary Tables S2–S4).

### Subgroup analysis

#### Leukemia

Leukemia emerged as the most economically burdensome subtype of HM, contributing 42.94% of the total global HM economic burden in 2021. Its associated costs rose from $462.63 billion in 1990 to $651 billion in 2021, reflecting a 40.7% increase over the three decades. Although the global burden of leukemia is projected to remain relatively stable at $496.86 billion by 2050, notable regional shifts are expected to occur. In 2021, the United States incurred the highest national economic burden related to leukemia, with costs reaching $166.56 billion (95% UI: $155.46–$172.87 billion), followed by China at $75.78 billion (95% UI: $55.42–$94.04 billion) and Germany at $46.35 billion (95% UI: $42.30–$49.78 billion). By 2050, China is projected to become the largest contributor globally, with leukemia-related costs estimated at $192.20 billion (95% UI: $140.31–$421.44 billion), followed by the United States ($69.15 billion, 95% UI: $48.40–$147.22 billion) and India ($32.05 billion, 95% UI: $23.40–$69.79 billion). In terms of burden relative to GDP in 2021, the highest proportions were observed in Tajikistan (14.83%, 95% UI: 9.41%−23.60%), Puerto Rico (6.19%, 95% UI: 5.11%−7.27%), and Croatia (2.46%, 95% UI: 2.07%−2.92%), reflecting its disproportionate impact on smaller and developing economies (see Supplementary Tables S2–S5 for details).

#### Non-Hodgkin lymphoma

Representing over one-third of the global economic burden of HM, NHL accounted for 35.81% of the total HM burden in 2021. Its economic impact rose from $337.66 billion in 1990 to $542.91 billion in 2021, indicating a 60.8% increase over the period. Despite this historical rise, NHL-related costs are projected to decrease to $496.86 billion by 2050, primarily due to enhanced treatment efficiency and broader access to care in HICs. Nevertheless, rising costs in UMICs, particularly China and India, will offset these declines. In 2021, the United States bore the highest national economic burden of NHL, totaling $154.70 billion (95% UI: $144.16–$162.50 billion), followed by Japan with $45.00 billion (95% UI: $39.66–$48.56 billion) and China with $43.89 billion (95% UI: $34.27–$53.33 billion). By 2050, China is projected to assume the leading position, with NHL-related costs reaching $152.54 billion (95% UI: $115.93–$560.88 billion), ahead of the United States ($63.91 billion, 95% UI: $44.74–$167.49 billion) and India ($27.57 billion, 95% UI: $20.13–$71.78 billion). When assessed as a proportion of GDP in 2021, the highest NHL-related economic burdens were observed in Tajikistan (7.97%, 95% UI: 6.02%−10.34%), Puerto Rico (6.41%, 95% UI: 5.35%−7.60%), and Croatia (1.99%, 95% UI: 1.70%−2.30%), highlighting the disproportionate impact on smaller economies. More details are provided in Supplementary Tables S2–S5.

### Multiple myeloma

Although MM accounted for a smaller proportion of the global HM burden in 2021 (18.35%), it experienced a substantial increase in economic cost, largely driven by rising prevalence and the adoption of advanced therapies. Between 1990 and 2021, the economic burden of MM grew from $138.71 billion to $278.17 billion, representing a 100.5% increase. However, this burden is projected to decline modestly to $250.00 billion by 2050, likely reflecting improvements in treatment efficiency and accessibility. In 2021, the United States accounted for the largest share of MM-related costs, totaling $86.56 billion (95% UI: $80.76–$90.32 billion), followed by Germany at $24.25 billion (95% UI: $21.78–$26.30 billion) and Japan at $15.79 billion (95% UI: $13.69–$17.10 billion). By 2050, China is projected to lead in MM-related costs, with an estimated burden of $70.62 billion (95% UI: $53.67–$493.54 billion), while the United States and India are expected to contribute $46.55 billion (95% UI: $32.59–$118.51 billion) and $19.03 billion (95% UI: $13.89–$42.52 billion), respectively. In 2021, the MM-related economic burden as a proportion of GDP was highest in Puerto Rico (3.91%, 95% UI: 3.25%−4.62%), followed by Lithuania (1.22%, 95% UI: 1.07%−1.38%) and Croatia (1.21%, 95% UI: 1.04%−1.44%), underscoring the significant economic pressure on smaller economies (full information are in Supplementary Tables S2–S5).

### Hodgkin lymphoma

HL contributed the smallest share to the global economic burden of HM in 2021, accounting for 2.89%, yet it remained a notable concern, particularly in LMICs and LICs. Its economic burden declined from $55.48 billion in 1990 to $43.84 billion in 2021, representing a 21.0% decrease. This downward trend is expected to continue, with projections indicating a further reduction to $36.69 billion by 2050, largely due to decreasing incidence and advancements in treatment strategies. In 2021, the United States incurred the highest national economic burden related to HL, with costs totaling $9.60 billion (95% UI: $9.10–$10.12 billion), followed by Russia at $3.28 billion (95% UI: $3.02–$3.56 billion) and Germany at $2.63 billion (95% UI: $2.18–$3.16 billion). By 2050, China is projected to lead in HL-related costs, with an estimated burden of $6.29 billion (95% UI: $4.78–$19.49 billion), followed by the United States ($3.51 billion, 95% UI: $2.45–$9.80 billion) and India ($3.31 billion, 95% UI: $2.41–$7.53 billion). In terms of economic burden relative to GDP in 2021, the highest proportions were observed in Puerto Rico (0.58%, 95% UI: 0.46%−0.72%), Tajikistan (0.47%, 95% UI: 0.29%−0.75%), and Bahrain (0.31%, 95% UI: 0.19%−0.48%), highlighting HL's disproportionate impact on smaller economies. Full details are available in Supplementary Tables S2–S5.

### Decomposition analysis

The decomposition analysis (Table 1) showed that the total change in costs was primarily driven by changes in the VSLY, which accounted for 70.65% of the overall increase. The remaining 29.35% was attributed to changes in DALYs. Within the DALY-related factors, population growth contributed most significantly (36.72%), followed by population aging (9.09%), both of which reflect demographic pressures that exacerbate the disease burden. In contrast, epidemiological changes had a negative effect, reducing the overall cost burden by 16.46%, likely reflecting improvements in disease prevention, early diagnosis, or treatment effectiveness. These findings suggest that rising economic costs are largely driven by increasing valuations of life and demographic expansion, while gains in public health and clinical care have modestly mitigated the burden. For decomposition analysis results of other hematologic malignancy subtypes, see Supplementary Tables S8–S11.

**Table 1:** Decomposition of the global economic burden of HMs.

**Factor category**	**Contribution to total cost change (%)**
Total cost change	100.00%
Change due to VSLY	70.65%
Change due to DALYs	29.35%
Population growth	36.72%
Population aging	9.09%
Epidemiological changes	−16.46%

## Discussion

This study provides an in-depth economic assessment of the burden of HMs, emphasizing the significant financial implications across countries with varying income levels. By leveraging the VSL and VSLY, our findings reveal stark disparities between HICs and LMICs in both the magnitude and composition of the economic burden of HMs. These disparities are shaped by differences in income levels, healthcare infrastructure, and development priorities.

HMs impose a substantial economic burden globally, but the extent and nature of this burden vary widely across countries. High-income countries experience a higher absolute economic burden due to their larger VSL and VSLY values, which are driven by higher gross national income (GNI) per capita and longer life expectancy compared to LMICs. For example, the adjusted VSL in the United States for 2021 is approximately $11.23 million, reflecting the country's high willingness to pay for mortality risk reduction (17, 20). In contrast, LMICs, despite having lower VSL estimates due to their lower GNI, face a relatively higher economic burden relative to their national income. This reflects an inequitable distribution of resources, where the proportional economic impact of HMs is far more severe in LMICs (24, 25).

Furthermore, the income elasticity of VSL plays a critical role in shaping these disparities. Previous studies have shown that the elasticity is typically higher in LMICs (ranging from 1.5 to 3) compared to HICs (elasticity≈1), which magnifies the relative economic losses in LMICs as income grows (17, 26). This underscores the urgent need for targeted policies to mitigate the disproportionate financial burden on LMICs and address the inequalities in health outcomes.

In high-income settings, the economic burden of HMs is primarily driven by the direct costs of medical care and the loss of productivity among working-age populations. Advanced healthcare systems in these countries enable prolonged survival and extensive treatment options, which, while improving health outcomes, also significantly increase healthcare expenditure. For example, therapies such as hematopoietic stem cell transplantation and immunotherapy are among the most effective treatments for HMs but are associated with substantial costs (13, 20). Additionally, the aging population in HICs further exacerbates the economic burden, as older patients often require prolonged and costly care.

While these treatment-related costs differ across HM subtypes, our study adopts a welfare economics perspective, using DALYs and VSLY to estimate population-level economic losses. The VSLY-based method does not capture micro-level treatment expenditures, and thus cannot account for heterogeneity in costs across subtypes. We acknowledge this as a methodological limitation and have added a clarification in the discussion to reflect this and recommend it as a direction for future research.

Conversely, in LMICs, the economic burden is compounded by limited access to effective treatments, delayed diagnoses, and a lack of robust healthcare infrastructure. The majority of patients in LMICs experience late-stage diagnoses, which not only worsens health outcomes but also increases indirect costs, such as loss of productivity and caregiving expenses. These indirect costs, when coupled with the relatively low VSL estimates in LMICs, highlight the dual challenge of addressing both disease treatment and economic losses (12, 25). Policies that focus on improving early detection and access to affordable treatments are essential to reducing the economic impact in these settings.

Moreover, our projections suggest a notable shift in the economic burden of hematologic malignancies from HICs to UMICs by 2050. While both groups are expected to see reductions in total DALYs, the decline is more substantial in HICs: 65.44% compared to 41.53% in UMICs. This may reflect the greater effectiveness of early detection and treatment in high-income settings, which helps reduce disease burden more rapidly.

At the same time, the VSLY is projected to increase dramatically in UMICs, rising by 184.65% between 2021 and 2050, compared to a 10.31% increase in HICs. This sharp rise, driven by economic growth and improved living standards, amplifies the economic valuation of health losses in UMICs. As a result, despite declining DALYs, the growing VSLY leads to an overall increase in the economic burden, eventually surpassing that of HICs.

At the global level, the total economic burden of HMs is projected to decline by 2050. After applying a 3% discount rate to bring future costs to 2021 values, total global DALYs are expected to decrease from 5,656,817.724 in 2021 to 1,955,049.023 in 2050: a 65% reduction. Meanwhile, the global average VSLY is projected to increase from USD 580,735,308.3 in 2021 to USD 827,421,443 in 2050, marking a 42.48% rise. Despite the increase in VSLY, the substantial reduction in DALYs outweighs the VSLY growth, resulting in a net decline in the aggregate global economic burden. This trend reflects the potential impact of ongoing advancements in prevention, early detection, therapy, and demographic transitions, particularly in high-income settings.

The findings of this study have significant implications for global health policy. The use of VSL and VSLY as tools for quantifying the economic burden of HMs provides a broader perspective on the societal cost of these diseases, beyond traditional health metrics such as DALYs and mortality rates. Policymakers should prioritize interventions that not only reduce disease burden but also alleviate the economic strain on vulnerable populations. For instance, targeted investments in early detection programs, such as population-wide screening for hematologic malignancies, could significantly reduce both mortality and economic losses, particularly in LMICs where late-stage diagnoses are prevalent (21, 26).

Additionally, international collaborations and funding mechanisms are crucial for addressing the disproportionate financial burden in LMICs. Programs such as the Global Fund or the World Bank's health financing initiatives could be expanded to include specific funding for HMs, with a focus on improving access to affordable treatments and diagnostic tools (12, 13). At the same time, high-income countries should explore ways to optimize the cost-effectiveness of treatments, such as reducing the price of innovative therapies and increasing the efficiency of healthcare delivery systems.

### Limitations and future directions

While this study offers valuable insights into the economic burden of HMs, several limitations should be noted. First, the estimation of the VSL and the VSLY relies heavily on income levels and elasticity parameters, which vary across studies and may affect the accuracy of the results. Second, the analysis primarily focuses on direct and indirect costs related to mortality and morbidity, while other indirect costs, such as the psychological impact on caregivers or long-term disability, are not fully captured. Future research should incorporate these additional dimensions for a more comprehensive assessment.

Additionally, while this study applies a uniform approach to estimate VSL and VSLY across countries, differences in healthcare systems, cultural attitudes toward healthcare spending, and preferences for mortality risk reduction may limit the generalizability of the findings. Region-specific models that account for these contextual factors should be explored in future studies. Another limitation is that the DALY estimates from the GBD 2021 database may not fully reflect disease burden variations due to underreporting in LMICs. This study relies on estimates from the GBD project, which, despite its comprehensive scope, is subject to several limitations. In some countries—particularly low-income regions—data quality may be limited due to weak health information systems and incomplete vital registration. In such settings, GBD estimates are often derived from statistical models that incorporate self-reported data, which can be influenced by individual perceptions and reporting biases. Although the GBD methodology addresses data gaps using standardized approaches such as statistical imputation, smoothing, and hierarchical modeling, the resulting estimates may still carry considerable uncertainty. These limitations are especially relevant for country-level comparisons and for long-term projections, and should be carefully considered when interpreting the findings. Finally, our projections based on the BAPC model are subject to several limitations (10). The inherent identifiability issue among age, period, and cohort effects may affect the stability and interpretability of long-term forecasts, particularly in countries with limited historical data. Additionally, while the INLA algorithm improves computational efficiency, it may underestimate uncertainty in complex hierarchical models. These factors should be considered when interpreting country-level projections, especially under scenarios where future demographic or economic trends deviate from historical patterns.

## Conclusions

The economic burden of HMs presents a significant challenge for global health systems, with LMICs disproportionately affected relative to their income levels. Addressing this issue requires a multifaceted approach, including targeted investments in early detection, access to affordable treatments, and international collaboration to reduce health inequalities. Integrating economic evaluations into health policy decisions can help governments and global health organizations prioritize interventions that optimize health outcomes while minimizing financial losses. Future research should also incorporate indirect costs, such as those related to mental health and long-term disability, to provide a more comprehensive understanding of the global impact of HMs.

## Data Availability

The original contributions presented in the study are included in the article/Supplementary material, further inquiries can be directed to the corresponding author.
