# Supplementary material for: Global, regional, and national economic burden of hematologic malignancies (1990–2021) with projections to 2050
Source: Front Public Health. 2025 Jun 27;13:1570792. doi: 10.3389/fpubh.2025.1570792 (PMC12245848; doi:10.3389/fpubh.2025.1570792)
Supplement: Supplementary file 1 [file Data_Sheet_1.docx]

Supplement

[Table S1 Economic burden of hematologic malignancies and its percentage of GDP in various countries in 1990 and 2021 (Discounted to 2021 US$) 2](#_Toc8354)

[Table S2 Projected Economic Burden of Hematologic Malignancies in Various Countries, 2030 (Discounted to 2021 US$) 14](#_Toc6965)

[Table S3 Projected Economic Burden of Hematologic Malignancies in Various Countries, 2040 (Discounted to 2021 US$) 26](#_Toc1097)

[Table S4 Projected Economic Burden of Hematologic Malignancies in Various Countries, 2050 (Discounted to 2021 US$) 39](#_Toc12308)

[Table S5 Economic burden of four subtypes of cancer and their percentage of GDP in various countries in 2021 51](#_Toc25420)

[Table S6 Estimated economic burden by country groups from 1990 to 2050 64](#_Toc8901)

[Table S7 Proportion of HM Economic Burden Attributed to Countries of Different Income Levels 64](#_Toc8745)

[Table S8 Decomposition of the Global Economic Burden of Hodgkin Lymphoma 64](#_Toc20228)

[Table S9 Decomposition of the Global Economic Burden of Non-Hodgkin Lymphoma 65](#_Toc23060)

[Table S10 Decomposition of the Global Economic Burden of Multiple Myeloma 65](#_Toc3056)

[Table S11 Decomposition of the Global Economic Burden of Leukemia 65](#_Toc9573)

# Table S1 Economic burden of hematologic malignancies and its percentage of GDP in various countries in 1990 and 2021 (Discounted to 2021 US$)

| Country | 1990 Economic Burden ($) | 1990 Proportion of Economic Burden to National GDP | 2021 Economic Burden ($) | 2021 Proportion of Economic Burden to National GDP |
| --- | --- | --- | --- | --- |
| Afghanistan | NA | NA | 172,441,701 (102,774,551, 260,436,228) | 1.6E-04 (9.3E-05, 2.4E-04) |
| Albania | 46,502,904 (36,703,402, 59,408,124) | 5.3E-05 (4.2E-05, 6.7E-05) | 110,634,072 (77,663,891, 157,834,932) | 1.3E-04 (8.8E-05, 1.8E-04) |
| Algeria | 1,975,598,726 (1,459,940,710, 2,491,133,342) | 2.4E-04 (1.8E-04, 3.1E-04) | 1,544,097,081 (1,135,848,170, 2,020,844,336) | 1.9E-04 (1.4E-04, 2.5E-04) |
| Andorra | NA | NA | 82,865,704 (54,841,826, 115,116,579) | 3.2E-02 (2.1E-02, 4.5E-02) |
| Angola | 230,147,027 (136,019,462, 353,827,943) | 1.6E-04 (9.2E-05, 2.4E-04) | 369,062,121 (237,180,998, 529,423,183) | 2.5E-04 (1.6E-04, 3.6E-04) |
| Antigua and Barbuda | 13,717,851 (12,660,126, 14,883,332) | 4.3E-03 (3.9E-03, 4.6E-03) | 22,705,014 (21,236,075, 24,357,212) | 7.1E-03 (6.6E-03, 7.6E-03) |
| Argentina | 4,688,869,974 (4,428,007,127, 4,967,309,319) | 6.8E-03 (6.4E-03, 7.2E-03) | 7,729,004,025 (7,158,162,130, 8,387,962,471) | 1.1E-02 (1.0E-02, 1.2E-02) |
| Armenia | 86,302,193 (79,739,596, 93,080,219) | 2.1E-05 (2.0E-05, 2.3E-05) | 206,138,314 (176,925,747, 237,675,281) | 5.1E-05 (4.4E-05, 5.9E-05) |
| Australia | 11,969,999,774 (11,414,973,751, 12,534,368,959) | 5.2E-03 (5.0E-03, 5.5E-03) | 22,190,269,717 (20,000,515,826, 24,264,105,627) | 9.7E-03 (8.7E-03, 1.1E-02) |
| Austria | 7,057,435,566 (6,679,648,606, 7,438,028,874) | 1.9E-02 (1.8E-02, 2.0E-02) | 9,363,219,525 (8,454,814,916, 10,157,596,862) | 2.6E-02 (2.3E-02, 2.8E-02) |
| Azerbaijan | 532,759,747 (415,158,759, 668,898,851) | 1.7E-02 (1.4E-02, 2.2E-02) | 802,135,987 (554,959,133, 1,141,759,217) | 2.6E-02 (1.8E-02, 3.7E-02) |
| Bahamas | 160,220,846 (146,654,846, 176,139,472) | 1.4E-02 (1.3E-02, 1.6E-02) | 145,410,173 (115,865,029, 182,214,800) | 1.3E-02 (1.0E-02, 1.6E-02) |
| Bahrain | 288,129,434 (217,927,096, 353,816,631) | 2.3E-02 (1.7E-02, 2.8E-02) | 529,034,120 (378,800,939, 712,569,106) | 4.2E-02 (3.0E-02, 5.6E-02) |
| Bangladesh | 610,854,786 (380,232,750, 896,377,040) | 2.2E-05 (1.3E-05, 3.2E-05) | 2,572,205,791 (1,769,565,887, 3,794,510,606) | 9.1E-05 (6.2E-05, 1.3E-04) |
| Barbados | 68,053,968 (63,673,477, 72,365,762) | 8.6E-03 (8.1E-03, 9.2E-03) | 78,040,525 (60,750,331, 97,761,362) | 9.9E-03 (7.7E-03, 1.2E-02) |
| Belarus | 945,014,954 (865,947,164, 1,025,892,706) | 7.5E-03 (6.9E-03, 8.1E-03) | 2,442,491,920 (1,980,867,258, 3,004,839,871) | 1.9E-02 (1.6E-02, 2.4E-02) |
| Belgium | 10,008,454,309 (9,338,202,029, 10,681,236,646) | 2.2E-02 (2.1E-02, 2.4E-02) | 12,987,028,735 (11,580,497,097, 14,313,819,150) | 2.9E-02 (2.6E-02, 3.2E-02) |
| Belize | 6,062,043 (5,540,321, 6,777,324) | 1.3E-03 (1.2E-03, 1.5E-03) | 9,799,775 (8,710,460, 11,057,235) | 2.1E-03 (1.9E-03, 2.4E-03) |
| Benin | 17,797,949 (12,744,148, 24,470,869) | 1.9E-06 (1.4E-06, 2.6E-06) | 47,393,434 (27,272,570, 65,424,800) | 5.1E-06 (2.9E-06, 7.0E-06) |
| Bermuda | NA | NA | 105,532,109 (88,175,779, 129,531,009) | NA |
| Bhutan | 5,798,164 (3,463,566, 8,459,780) | 3.4E-05 (2.0E-05, 5.0E-05) | 26,342,180 (16,207,433, 44,410,529) | 1.6E-04 (9.6E-05, 2.6E-04) |
| Bolivia (Plurinational State of) | 210,295,885 (145,993,373, 286,757,704) | 4.4E-03 (3.1E-03, 6.0E-03) | 543,892,525 (381,768,851, 743,820,125) | 1.1E-02 (8.0E-03, 1.6E-02) |
| Bosnia and Herzegovina | 16,332,742 (13,056,763, 20,344,285) | 4.8E-04 (3.8E-04, 5.9E-04) | 340,900,801 (244,617,520, 440,672,221) | 9.9E-03 (7.1E-03, 1.3E-02) |
| Botswana | 57,225,852 (39,597,224, 82,305,987) | 3.0E-04 (2.1E-04, 4.4E-04) | 135,124,177 (92,639,825, 194,281,057) | 7.2E-04 (4.9E-04, 1.0E-03) |
| Brazil | 12,289,766,022 (11,755,133,525, 12,854,515,050) | 1.0E-02 (9.6E-03, 1.1E-02) | 17,022,111,988 (16,216,483,732, 17,704,545,772) | 1.4E-02 (1.3E-02, 1.5E-02) |
| Brunei Darussalam | 428,740,734 (344,407,538, 560,322,384) | 2.3E-02 (1.8E-02, 2.9E-02) | 375,457,532 (296,363,776, 450,747,827) | 2.0E-02 (1.6E-02, 2.4E-02) |
| Bulgaria | 1,263,201,173 (1,145,399,110, 1,393,366,924) | 1.2E-02 (1.1E-02, 1.3E-02) | 2,197,472,400 (1,805,320,907, 2,647,614,739) | 2.1E-02 (1.7E-02, 2.5E-02) |
| Burkina Faso | 12,014,026 (8,582,070, 16,545,893) | 1.2E-06 (8.9E-07, 1.7E-06) | 48,410,803 (28,959,494, 66,041,449) | 5.0E-06 (3.0E-06, 6.8E-06) |
| Burundi | 21,024,389 (14,010,226, 29,990,128) | 1.1E-05 (7.6E-06, 1.6E-05) | 11,218,517 (6,548,132, 17,656,868) | 6.1E-06 (3.6E-06, 9.6E-06) |
| Cabo Verde | 870,922 (643,012, 1,245,670) | 5.0E-06 (3.7E-06, 7.1E-06) | 6,160,282 (3,915,230, 8,036,642) | 3.5E-05 (2.2E-05, 4.6E-05) |
| Cambodia | 48,816,174 (29,361,313, 74,575,208) | 3.8E-07 (2.3E-07, 5.7E-07) | 198,929,077 (140,100,873, 283,332,990) | 1.5E-06 (1.1E-06, 2.2E-06) |
| Cameroon | 80,212,357 (59,311,964, 103,650,228) | 3.4E-06 (2.5E-06, 4.5E-06) | 193,957,574 (118,498,181, 267,762,880) | 8.3E-06 (5.1E-06, 1.2E-05) |
| Canada | 24,242,889,274 (22,967,955,558, 25,502,821,261) | 1.1E-02 (1.0E-02, 1.1E-02) | 33,283,333,838 (30,111,607,896, 36,343,955,141) | 1.5E-02 (1.3E-02, 1.6E-02) |
| Central African Republic | 5,506,064 (3,573,429, 8,163,443) | 7.1E-06 (4.6E-06, 1.0E-05) | 5,068,326 (3,294,454, 7,366,350) | 6.5E-06 (4.2E-06, 9.5E-06) |
| Chad | 9,112,427 (6,273,558, 12,750,523) | 1.1E-06 (7.4E-07, 1.5E-06) | 25,182,199 (14,949,588, 37,022,324) | 3.0E-06 (1.8E-06, 4.4E-06) |
| Chile | 641,055,081 (608,050,721, 674,546,284) | 3.2E-06 (3.1E-06, 3.4E-06) | 5,089,552,208 (4,690,492,825, 5,505,987,198) | 2.6E-05 (2.4E-05, 2.8E-05) |
| China | 8,723,170,740 (6,658,517,934, 10,643,853,340) | 8.8E-05 (6.7E-05, 1.1E-04) | 133,843,000,000 (98,626,908,315, 166,207,000,000) | 1.3E-03 (9.9E-04, 1.7E-03) |
| Colombia | 2,181,811,684 (2,047,494,140, 2,319,135,663) | 2.4E-06 (2.3E-06, 2.6E-06) | 4,374,565,033 (3,667,822,638, 5,213,804,213) | 4.8E-06 (4.0E-06, 5.8E-06) |
| Comoros | 4,637,869 (2,689,971, 6,504,075) | 1.1E-05 (6.3E-06, 1.5E-05) | 5,295,825 (3,442,057, 7,556,513) | 1.2E-05 (8.1E-06, 1.8E-05) |
| Congo | 42,576,746 (30,194,246, 60,784,885) | 9.6E-06 (6.8E-06, 1.4E-05) | 55,691,282 (38,465,787, 77,879,222) | 1.3E-05 (8.7E-06, 1.8E-05) |
| Costa Rica | 167,952,001 (157,187,852, 178,895,673) | 4.5E-06 (4.2E-06, 4.8E-06) | 780,850,324 (694,256,141, 868,698,602) | 2.1E-05 (1.9E-05, 2.3E-05) |
| Coted' Ivoire | 141,050,694 (102,806,418, 184,844,587) | 3.8E-06 (2.7E-06, 4.9E-06) | 275,203,239 (184,099,736, 394,454,724) | 7.3E-06 (4.9E-06, 1.0E-05) |
| Croatia | 1,223,413,482 (1,084,277,135, 1,377,980,252) | 2.3E-02 (2.0E-02, 2.5E-02) | 3,145,209,982 (2,668,842,536, 3,697,390,504) | 5.8E-02 (4.9E-02, 6.8E-02) |
| Cyprus | 365,599,838 (297,608,210, 498,081,663) | 1.5E-02 (1.2E-02, 2.1E-02) | 746,917,209 (542,202,012, 942,764,086) | 3.1E-02 (2.3E-02, 3.9E-02) |
| Czechia | 5,318,801,100 (4,899,297,036, 5,783,494,639) | 1.0E-03 (9.4E-04, 1.1E-03) | 9,901,810,045 (8,324,741,126, 11,603,123,417) | 1.9E-03 (1.6E-03, 2.2E-03) |
| Democratic Republic of the Congo | 81,215,984 (55,480,229, 116,596,882) | 5.7E-06 (3.9E-06, 8.2E-06) | 71,034,046 (46,696,813, 101,736,857) | 5.0E-06 (3.3E-06, 7.1E-06) |
| Denmark | 4,747,307,323 (4,499,271,114, 4,998,102,900) | 2.1E-03 (2.0E-03, 2.3E-03) | 8,229,300,781 (7,473,344,537, 8,995,320,809) | 3.7E-03 (3.4E-03, 4.1E-03) |
| Djibouti | NA | NA | 17,843,293 (10,695,146, 28,226,084) | 3.2E-05 (1.9E-05, 5.1E-05) |
| Dominica | 4,686,865 (3,865,294, 5,647,342) | 4.2E-03 (3.5E-03, 5.1E-03) | 7,779,284 (5,836,567, 10,244,422) | 7.0E-03 (5.3E-03, 9.2E-03) |
| Dominican Republic | 205,249,946 (164,360,304, 254,440,097) | 7.5E-05 (6.0E-05, 9.2E-05) | 850,399,682 (631,714,995, 1,115,317,627) | 3.1E-04 (2.3E-04, 4.1E-04) |
| Ecuador | 418,086,895 (390,703,886, 447,592,343) | 3.9E-03 (3.6E-03, 4.2E-03) | 1,144,720,071 (928,019,281, 1,397,940,922) | 1.1E-02 (8.6E-03, 1.3E-02) |
| Egypt | 1,578,860,904 (1,285,214,531, 2,419,499,612) | 2.1E-04 (1.7E-04, 3.3E-04) | 5,499,166,243 (3,711,217,724, 7,821,961,858) | 7.5E-04 (5.0E-04, 1.1E-03) |
| El Salvador | 146,858,287 (124,066,103, 185,452,159) | 5.6E-03 (4.7E-03, 7.1E-03) | 238,932,603 (173,486,298, 298,981,827) | 9.1E-03 (6.6E-03, 1.1E-02) |
| Equatorial Guinea | 763,191 (502,551, 1,130,526) | 1.6E-07 (1.0E-07, 2.3E-07) | 42,274,094 (23,247,846, 67,773,209) | 8.8E-06 (4.8E-06, 1.4E-05) |
| Estonia | 306,486,959 (281,635,029, 333,690,383) | 1.2E-02 (1.1E-02, 1.3E-02) | 1,223,442,269 (1,043,517,703, 1,399,819,136) | 4.7E-02 (4.0E-02, 5.4E-02) |
| Eswatini | 18,239,345 (13,165,612, 27,032,620) | 3.9E-04 (2.8E-04, 5.8E-04) | 39,001,370 (23,825,760, 58,094,575) | 8.4E-04 (5.1E-04, 1.2E-03) |
| Ethiopia | 182,367,262 (90,609,232, 283,210,351) | 8.6E-05 (4.3E-05, 1.3E-04) | 717,979,905 (499,574,683, 1,015,411,289) | 3.4E-04 (2.4E-04, 4.8E-04) |
| Fiji | 25,876,847 (15,482,742, 35,388,730) | 3.1E-03 (1.8E-03, 4.2E-03) | 31,927,496 (19,011,062, 44,354,072) | 3.8E-03 (2.3E-03, 5.3E-03) |
| Finland | 4,383,984,672 (4,156,348,134, 4,623,384,340) | 1.9E-02 (1.8E-02, 2.0E-02) | 5,970,826,524 (5,372,602,595, 6,535,913,711) | 2.6E-02 (2.3E-02, 2.8E-02) |
| France | 50,700,670,826 (47,949,036,617, 53,320,766,782) | 2.2E-02 (2.1E-02, 2.3E-02) | 67,008,296,769 (59,286,622,387, 74,620,356,314) | 2.9E-02 (2.6E-02, 3.3E-02) |
| Gabon | 114,925,383 (85,498,443, 155,804,302) | 2.0E-05 (1.5E-05, 2.7E-05) | 86,019,536 (55,081,154, 123,862,278) | 1.5E-05 (9.6E-06, 2.2E-05) |
| Gambia | 3,900,717 (2,731,423, 5,197,948) | 6.0E-05 (4.2E-05, 7.9E-05) | 4,946,777 (3,407,766, 6,970,637) | 7.6E-05 (5.2E-05, 1.1E-04) |
| Georgia | 591,173,043 (508,232,574, 670,350,869) | 1.1E-02 (9.9E-03, 1.3E-02) | 479,842,453 (415,274,859, 552,701,034) | 9.3E-03 (8.1E-03, 1.1E-02) |
| Germany | 82,143,004,906 (77,613,532,706, 87,269,072,609) | 2.6E-02 (2.4E-02, 2.7E-02) | 113,025,000,000 (101,624,000,000, 122,878,000,000) | 3.5E-02 (3.2E-02, 3.8E-02) |
| Ghana | 134,772,570 (66,675,564, 189,085,790) | 7.7E-04 (3.8E-04, 1.1E-03) | 268,716,603 (187,401,150, 366,030,275) | 1.5E-03 (1.1E-03, 2.1E-03) |
| Greenland | 35,526,330 (27,312,602, 43,615,742) | NA | NA | NA |
| Grenada | 8,044,098 (7,177,497, 9,022,526) | 3.6E-03 (3.2E-03, 4.0E-03) | 11,187,539 (9,688,678, 12,832,991) | 5.0E-03 (4.3E-03, 5.7E-03) |
| Guatemala | 212,960,402 (196,560,439, 228,635,199) | 3.9E-04 (3.6E-04, 4.2E-04) | 546,327,017 (469,138,314, 635,809,950) | 1.0E-03 (8.6E-04, 1.2E-03) |
| Guinea | 13,915,235 (9,685,128, 18,651,048) | 1.9E-07 (1.3E-07, 2.5E-07) | 32,796,428 (20,600,775, 52,973,813) | 4.4E-07 (2.8E-07, 7.1E-07) |
| Guinea-Bissau | 2,739,802 (1,852,650, 3,880,999) | 3.1E-06 (2.1E-06, 4.4E-06) | 4,466,638 (2,909,376, 6,015,366) | 5.1E-06 (3.3E-06, 6.8E-06) |
| Guyana | 14,683,147 (12,406,970, 16,707,750) | 8.2E-06 (6.9E-06, 9.3E-06) | 82,144,213 (62,726,376, 105,447,990) | 4.6E-05 (3.5E-05, 5.9E-05) |
| Haiti | 245,994,377 (120,266,005, 394,117,846) | 4.0E-04 (2.0E-04, 6.4E-04) | 127,745,079 (75,552,616, 196,921,166) | 2.1E-04 (1.2E-04, 3.2E-04) |
| Honduras | 69,800,112 (56,771,575, 87,688,651) | 3.1E-04 (2.5E-04, 3.9E-04) | 107,817,216 (72,350,792, 162,629,732) | 4.8E-04 (3.2E-04, 7.2E-04) |
| Hungary | 3,164,805,936 (2,904,087,926, 3,462,997,864) | 7.5E-05 (6.9E-05, 8.2E-05) | 7,101,622,346 (5,976,255,332, 8,380,378,604) | 1.7E-04 (1.4E-04, 2.0E-04) |
| Iceland | 155,195,162 (142,937,804, 167,935,093) | 5.8E-05 (5.3E-05, 6.3E-05) | 265,124,839 (234,585,652, 297,021,799) | 9.9E-05 (8.8E-05, 1.1E-04) |
| India | 4,528,792,667 (3,374,979,383, 5,695,498,407) | 3.0E-05 (2.2E-05, 3.8E-05) | 22,880,501,721 (18,690,853,410, 28,124,504,926) | 1.5E-04 (1.2E-04, 1.9E-04) |
| Indonesia | 4,658,660,414 (3,436,680,597, 6,387,868,800) | 4.2E-07 (3.1E-07, 5.7E-07) | 12,384,315,670 (9,625,875,508, 16,827,539,705) | 1.1E-06 (8.7E-07, 1.5E-06) |
| Iran (Islamic Republic of) | 7,233,968,152 (4,695,425,221, 9,365,414,654) | 4.7E-07 (3.1E-07, 6.1E-07) | 4,467,502,903 (3,066,512,167, 5,190,348,794) | 2.9E-07 (2.0E-07, 3.4E-07) |
| Iraq | NA | NA | 1,968,466,641 (1,424,874,204, 2,615,285,459) | 9.9E-06 (7.2E-06, 1.3E-05) |
| Ireland | 1,863,199,110 (1,760,938,394, 1,976,480,204) | 4.3E-03 (4.1E-03, 4.6E-03) | 5,016,492,742 (4,479,008,576, 5,563,798,176) | 1.2E-02 (1.0E-02, 1.3E-02) |
| Israel | 2,703,960,948 (2,537,315,735, 2,872,426,575) | 1.8E-03 (1.7E-03, 1.9E-03) | 5,074,268,047 (4,553,371,770, 5,517,054,254) | 3.4E-03 (3.1E-03, 3.7E-03) |
| Italy | 60,749,878,860 (58,374,206,402, 62,759,020,274) | 3.6E-02 (3.4E-02, 3.7E-02) | 64,583,740,689 (58,358,147,902, 69,181,489,256) | 3.8E-02 (3.4E-02, 4.1E-02) |
| Jamaica | 109,836,470 (100,483,611, 120,071,855) | 1.5E-04 (1.4E-04, 1.6E-04) | 112,631,132 (86,012,829, 145,959,113) | 1.5E-04 (1.2E-04, 2.0E-04) |
| Japan | 88,300,298,846 (85,651,174,297, 90,235,425,759) | 1.6E-04 (1.6E-04, 1.7E-04) | 96,589,090,669 (85,989,137,677, 103,455,000,000) | 1.8E-04 (1.6E-04, 1.9E-04) |
| Jordan | 177,886,200 (141,389,757, 226,455,159) | 5.8E-03 (4.6E-03, 7.3E-03) | 291,707,058 (213,380,212, 381,403,631) | 9.5E-03 (6.9E-03, 1.2E-02) |
| Kazakhstan | NA | NA | 2,286,405,154 (2,000,921,676, 2,605,851,976) | 1.5E-04 (1.3E-04, 1.7E-04) |
| Kenya | 113,476,903 (79,808,969, 150,378,077) | 1.2E-05 (8.5E-06, 1.6E-05) | 336,938,886 (243,991,070, 446,893,581) | 3.6E-05 (2.6E-05, 4.8E-05) |
| Kiribati | 367,510 (234,976, 472,772) | 1.1E-03 (7.0E-04, 1.4E-03) | 698,868 (382,324, 1,042,371) | 2.1E-03 (1.1E-03, 3.1E-03) |
| Kuwait | 1,583,962,723 (1,436,839,920, 1,735,193,402) | 4.1E-02 (3.7E-02, 4.5E-02) | 1,249,369,351 (1,034,066,763, 1,494,310,374) | 3.2E-02 (2.7E-02, 3.9E-02) |
| Kyrgyzstan | NA | NA | 49,781,521 (40,242,444, 60,391,333) | 2.7E-04 (2.2E-04, 3.3E-04) |
| Lao People's Democratic Republic | 32,184,238 (16,821,037, 51,652,217) | 2.4E-07 (1.3E-07, 3.9E-07) | 172,178,352 (119,675,222, 252,043,569) | 1.3E-06 (9.1E-07, 1.9E-06) |
| Latvia | 553,508,717 (507,066,626, 599,737,804) | 2.0E-02 (1.8E-02, 2.1E-02) | 1,468,371,066 (1,249,360,175, 1,709,790,505) | 5.2E-02 (4.5E-02, 6.1E-02) |
| Lebanon | 174,941,762 (121,592,431, 245,305,696) | 4.4E-06 (3.0E-06, 6.1E-06) | 331,718,251 (251,842,414, 428,183,688) | 8.3E-06 (6.3E-06, 1.1E-05) |
| Lesotho | 5,728,793 (4,031,962, 8,236,115) | 2.9E-04 (2.0E-04, 4.1E-04) | 11,027,416 (7,529,169, 15,332,024) | 5.5E-04 (3.8E-04, 7.7E-04) |
| Liberia | 12,323,029 (8,101,452, 17,245,860) | 3.8E-03 (2.5E-03, 5.3E-03) | 6,833,588 (3,975,750, 9,739,455) | 2.1E-03 (1.2E-03, 3.0E-03) |
| Libya | 1,899,570,947 (1,421,025,327, 2,469,664,100) | 2.3E-02 (1.7E-02, 3.0E-02) | 822,869,306 (587,680,029, 1,126,776,625) | 9.9E-03 (7.1E-03, 1.4E-02) |
| Lithuania | 716,930,306 (664,074,502, 769,800,760) | 1.6E-02 (1.4E-02, 1.7E-02) | 2,506,208,637 (2,173,872,049, 2,841,522,351) | 5.4E-02 (4.7E-02, 6.2E-02) |
| Luxembourg | 647,346,583 (612,731,780, 682,561,065) | 1.0E-02 (9.6E-03, 1.1E-02) | 935,872,625 (836,295,718, 1,034,590,681) | 1.5E-02 (1.3E-02, 1.6E-02) |
| Madagascar | 72,622,345 (53,178,648, 94,257,171) | 3.4E-06 (2.5E-06, 4.4E-06) | 48,167,003 (31,952,930, 66,518,357) | 2.2E-06 (1.5E-06, 3.1E-06) |
| Malawi | 51,159,062 (38,665,324, 65,785,243) | 6.7E-06 (5.1E-06, 8.7E-06) | 65,773,644 (40,841,422, 100,541,893) | 8.7E-06 (5.4E-06, 1.3E-05) |
| Malaysia | 1,553,113,244 (1,171,716,969, 1,958,715,318) | 1.1E-03 (8.4E-04, 1.4E-03) | 5,366,997,265 (4,315,530,624, 6,803,545,362) | 3.9E-03 (3.1E-03, 4.9E-03) |
| Maldives | 8,048,604 (4,291,133, 12,383,599) | 9.9E-05 (5.3E-05, 1.5E-04) | 15,588,525 (11,339,450, 20,815,328) | 1.9E-04 (1.4E-04, 2.6E-04) |
| Mali | 17,432,836 (11,567,977, 23,928,630) | 3.2E-06 (2.1E-06, 4.3E-06) | 32,551,908 (20,371,923, 51,850,459) | 5.9E-06 (3.7E-06, 9.4E-06) |
| Malta | 91,719,628 (84,188,733, 99,659,086) | 6.7E-03 (6.2E-03, 7.3E-03) | 335,009,410 (293,622,456, 378,477,062) | 2.5E-02 (2.2E-02, 2.8E-02) |
| Marshall Islands | 547,365 (400,662, 711,706) | 2.5E-03 (1.8E-03, 3.2E-03) | 924,779 (608,133, 1,308,726) | 4.2E-03 (2.7E-03, 5.9E-03) |
| Mauritania | 11,619,707 (8,581,956, 15,323,554) | 1.4E-04 (1.1E-04, 1.9E-04) | 24,601,008 (15,331,599, 35,467,823) | 3.1E-04 (1.9E-04, 4.4E-04) |
| Mauritius | 42,904,861 (40,935,546, 44,800,231) | 9.4E-05 (9.0E-05, 9.9E-05) | 187,662,412 (172,649,811, 198,373,950) | 4.1E-04 (3.8E-04, 4.4E-04) |
| Mexico | 11,882,445,353 (11,385,257,198, 12,446,955,580) | 5.1E-04 (4.9E-04, 5.3E-04) | 14,245,124,887 (12,799,937,965, 15,722,794,671) | 6.1E-04 (5.5E-04, 6.7E-04) |
| Micronesia (Federated States of) | 1,114,672 (794,567, 1,538,976) | 4.3E-03 (3.1E-03, 6.0E-03) | 782,198 (523,470, 1,075,197) | 3.0E-03 (2.0E-03, 4.2E-03) |
| Mongolia | 60,979,827 (44,485,804, 83,064,777) | 2.3E-06 (1.6E-06, 3.1E-06) | 99,580,687 (72,999,569, 130,773,746) | 3.7E-06 (2.7E-06, 4.8E-06) |
| Montenegro | NA | NA | 93,821,646 (72,673,829, 119,897,195) | 3.2E-02 (2.4E-02, 4.0E-02) |
| Morocco | 300,830,171 (219,791,533, 393,206,144) | 2.6E-04 (1.9E-04, 3.4E-04) | 568,073,291 (400,676,645, 774,504,475) | 4.8E-04 (3.4E-04, 6.6E-04) |
| Mozambique | NA | NA | 52,410,990 (30,148,296, 86,906,115) | 5.3E-05 (3.1E-05, 8.9E-05) |
| Myanmar | 97,460,214 (51,904,241, 150,693,779) | 1.2E-06 (6.6E-07, 1.9E-06) | 774,721,916 (562,730,848, 1,082,039,354) | 9.8E-06 (7.1E-06, 1.4E-05) |
| Namibia | 33,078,897 (24,582,584, 42,475,341) | 2.4E-04 (1.8E-04, 3.1E-04) | 67,808,096 (46,343,261, 98,184,981) | 4.9E-04 (3.4E-04, 7.1E-04) |
| Nauru | 2,852,303 (2,035,956, 3,847,022) | 2.1E-02 (1.5E-02, 2.8E-02) | 1,910,894 (1,220,731, 2,801,857) | 1.4E-02 (8.8E-03, 2.0E-02) |
| Nepal | 72,805,541 (45,972,387, 106,943,982) | 3.0E-05 (1.9E-05, 4.5E-05) | 226,233,518 (156,708,164, 351,218,015) | 9.4E-05 (6.5E-05, 1.5E-04) |
| Netherlands | 12,131,537,579 (11,485,675,474, 12,749,367,065) | 1.6E-02 (1.5E-02, 1.6E-02) | 19,138,104,498 (17,262,352,916, 20,865,545,330) | 2.5E-02 (2.2E-02, 2.7E-02) |
| New Zealand | 2,039,888,356 (1,914,814,803, 2,165,927,182) | 7.6E-03 (7.1E-03, 8.0E-03) | 3,664,765,389 (3,321,002,293, 3,961,151,696) | 1.4E-02 (1.2E-02, 1.5E-02) |
| Nicaragua | 54,873,971 (45,136,872, 70,462,348) | 2.9E-04 (2.4E-04, 3.7E-04) | 75,897,568 (55,129,712, 93,975,396) | 4.0E-04 (2.9E-04, 5.0E-04) |
| Niger | 17,663,743 (11,072,323, 25,188,854) | 2.3E-06 (1.5E-06, 3.3E-06) | 21,676,525 (11,248,377, 33,758,231) | 2.9E-06 (1.5E-06, 4.5E-06) |
| Nigeria | NA | NA | 2,473,097,727 (1,234,953,967, 3,589,279,628) | 3.4E-05 (1.7E-05, 4.9E-05) |
| North Macedonia | 136,262,683 (109,427,250, 173,949,448) | 2.9E-04 (2.3E-04, 3.7E-04) | 252,739,137 (180,119,885, 328,658,256) | 5.4E-04 (3.8E-04, 7.0E-04) |
| Norway | 3,819,593,270 (3,645,671,721, 3,963,768,931) | 8.8E-04 (8.4E-04, 9.2E-04) | 7,600,541,430 (6,960,318,883, 8,123,418,911) | 1.8E-03 (1.6E-03, 1.9E-03) |
| Oman | 859,540,787 (582,439,516, 1,196,224,841) | 2.5E-02 (1.7E-02, 3.5E-02) | 734,347,869 (533,030,306, 968,545,068) | 2.1E-02 (1.5E-02, 2.8E-02) |
| Pakistan | 1,635,703,001 (1,177,255,849, 2,147,059,839) | 4.5E-05 (3.2E-05, 5.9E-05) | 3,865,890,527 (2,867,143,210, 5,203,352,754) | 1.1E-04 (7.8E-05, 1.4E-04) |
| Palau | 1,545,700 (1,101,990, 2,062,297) | 6.8E-03 (4.9E-03, 9.1E-03) | 792,923 (605,005, 997,989) | 3.5E-03 (2.7E-03, 4.4E-03) |
| Panama | 130,058,269 (120,816,531, 140,479,847) | 2.0E-03 (1.8E-03, 2.1E-03) | 1,273,219,025 (1,042,189,689, 1,519,734,048) | 1.9E-02 (1.6E-02, 2.3E-02) |
| Papua New Guinea | 30,205,408 (16,233,375, 44,351,775) | 4.8E-04 (2.6E-04, 7.0E-04) | 53,996,911 (32,569,754, 76,618,658) | 8.6E-04 (5.2E-04, 1.2E-03) |
| Paraguay | 196,185,009 (155,745,985, 242,310,710) | 9.1E-07 (7.2E-07, 1.1E-06) | 378,983,395 (276,238,334, 495,897,718) | 1.8E-06 (1.3E-06, 2.3E-06) |
| Peru | 779,611,527 (637,301,526, 1,018,251,630) | 1.4E-03 (1.2E-03, 1.8E-03) | 2,417,235,583 (1,644,401,071, 3,193,284,329) | 4.4E-03 (3.0E-03, 5.8E-03) |
| Philippines | 1,376,055,406 (1,125,386,115, 1,741,334,028) | 7.4E-05 (6.1E-05, 9.4E-05) | 2,897,114,871 (2,347,284,412, 3,460,940,742) | 1.6E-04 (1.3E-04, 1.9E-04) |
| Poland | 4,685,830,879 (4,383,847,226, 4,877,081,168) | 2.1E-03 (1.9E-03, 2.2E-03) | 24,955,987,367 (22,785,634,562, 27,146,978,887) | 1.1E-02 (1.0E-02, 1.2E-02) |
| Portugal | 3,723,089,637 (3,526,779,929, 3,910,194,352) | 1.9E-02 (1.8E-02, 2.0E-02) | 7,379,423,702 (6,640,056,742, 8,070,957,665) | 3.7E-02 (3.4E-02, 4.1E-02) |
| Puerto Rico | 910,674,168 (856,712,778, 970,622,954) | 9.8E-02 (9.2E-02, 1.0E-01) | 1,583,177,729 (1,312,289,798, 1,871,530,583) | 1.7E-01 (1.4E-01, 2.0E-01) |
| Qatar | 442,890,325 (305,208,463, 582,825,405) | 6.7E-04 (4.6E-04, 8.8E-04) | 1,292,611,525 (872,512,289, 1,801,762,817) | 2.0E-03 (1.3E-03, 2.7E-03) |
| Republic of Korea | 5,833,975,166 (4,665,249,555, 7,289,250,918) | 3.0E-06 (2.4E-06, 3.8E-06) | 21,606,421,590 (14,051,177,194, 26,255,148,480) | 1.1E-05 (7.3E-06, 1.4E-05) |
| Republic of Moldova | 665,369,221 (620,491,227, 715,856,472) | 4.8E-02 (4.5E-02, 5.2E-02) | 306,510,597 (271,191,508, 348,709,257) | 2.2E-02 (2.0E-02, 2.5E-02) |
| Romania | 1,984,582,376 (1,846,024,215, 2,125,170,144) | 2.2E-03 (2.1E-03, 2.4E-03) | 10,211,714,933 (8,745,956,249, 11,618,001,881) | 1.1E-02 (9.8E-03, 1.3E-02) |
| Russian Federation | 27,053,790,254 (26,454,630,274, 27,716,703,328) | 2.0E-04 (1.9E-04, 2.0E-04) | 54,593,895,112 (50,529,956,687, 58,934,102,643) | 4.0E-04 (3.7E-04, 4.3E-04) |
| Rwanda | 26,113,524 (18,002,442, 35,489,376) | 2.7E-06 (1.8E-06, 3.6E-06) | 64,110,464 (41,342,683, 95,096,046) | 6.5E-06 (4.2E-06, 9.7E-06) |
| Saint Lucia | 14,262,646 (13,293,351, 15,424,162) | 3.0E-03 (2.8E-03, 3.3E-03) | 22,426,453 (18,514,322, 27,072,707) | 4.7E-03 (3.9E-03, 5.7E-03) |
| Saint Vincent and the Grenadines | 6,154,749 (5,626,448, 6,701,205) | 2.6E-03 (2.4E-03, 2.9E-03) | 12,171,357 (10,610,914, 13,992,565) | 5.2E-03 (4.6E-03, 6.0E-03) |
| Samoa | 4,049,886 (3,111,079, 5,718,679) | 2.1E-03 (1.6E-03, 2.9E-03) | 3,179,229 (2,363,001, 4,586,253) | 1.6E-03 (1.2E-03, 2.3E-03) |
| San Marino | NA | NA | 37,232,558 (24,221,231, 53,710,641) | 2.8E-02 (1.9E-02, 4.1E-02) |
| Sao Tome and Principe | 432,828 (320,701, 583,030) | 1.0E-04 (7.7E-05, 1.4E-04) | 931,892 (608,207, 1,381,842) | 2.2E-04 (1.5E-04, 3.3E-04) |
| Saudi Arabia | 9,646,725,394 (6,586,329,158, 14,140,766,689) | 3.0E-03 (2.0E-03, 4.3E-03) | 12,216,964,337 (8,425,089,633, 17,570,994,415) | 3.8E-03 (2.6E-03, 5.4E-03) |
| Senegal | 37,381,331 (27,534,052, 48,908,861) | 2.6E-06 (1.9E-06, 3.4E-06) | 58,952,126 (35,839,836, 86,629,226) | 4.1E-06 (2.5E-06, 6.1E-06) |
| Serbia | NA | NA | 1,677,621,750 (1,253,185,593, 2,093,837,515) | 3.2E-04 (2.4E-04, 4.0E-04) |
| Seychelles | 12,474,075 (10,498,367, 15,767,185) | 5.5E-04 (4.6E-04, 6.9E-04) | 36,214,323 (29,214,997, 43,753,616) | 1.6E-03 (1.3E-03, 1.9E-03) |
| Sierra Leone | 7,632,186 (5,192,685, 10,782,556) | 7.3E-07 (5.0E-07, 1.0E-06) | 10,290,081 (6,216,813, 14,552,980) | 9.9E-07 (6.0E-07, 1.4E-06) |
| Singapore | 1,757,989,136 (1,655,458,811, 1,865,932,645) | 3.5E-03 (3.3E-03, 3.7E-03) | 4,688,081,812 (4,279,593,788, 5,083,089,431) | 9.2E-03 (8.4E-03, 1.0E-02) |
| Slovakia | 990,160,761 (815,545,973, 1,276,708,732) | 1.1E-02 (9.0E-03, 1.4E-02) | 3,210,725,697 (2,301,169,831, 4,126,986,445) | 3.5E-02 (2.5E-02, 4.5E-02) |
| Solomon Islands | 1,395,006 (729,437, 2,082,459) | 1.3E-04 (6.9E-05, 2.0E-04) | 2,224,042 (1,400,774, 3,085,876) | 2.1E-04 (1.3E-04, 2.9E-04) |
| Somalia | 11,838,551 (7,417,739, 16,961,445) | 1.2E-03 (7.3E-04, 1.7E-03) | 39,350,168 (24,956,866, 57,610,843) | 3.9E-03 (2.5E-03, 5.7E-03) |
| South Africa | 2,295,917,820 (1,871,679,690, 2,767,723,877) | 5.1E-04 (4.1E-04, 6.1E-04) | 3,190,365,487 (2,364,719,130, 3,687,406,611) | 7.1E-04 (5.2E-04, 8.2E-04) |
| Spain | 23,265,359,473 (22,055,633,045, 24,459,046,598) | 3.9E-02 (3.7E-02, 4.1E-02) | 29,645,010,662 (26,475,388,363, 32,464,809,052) | 5.0E-02 (4.5E-02, 5.5E-02) |
| Sri Lanka | 334,191,293 (266,427,927, 409,307,224) | 2.6E-05 (2.0E-05, 3.1E-05) | 1,024,434,643 (672,556,878, 1,451,857,123) | 7.9E-05 (5.2E-05, 1.1E-04) |
| Sudan | 243,166,929 (130,346,551, 405,257,437) | 1.2E-02 (6.2E-03, 1.9E-02) | 276,199,540 (170,606,078, 402,947,888) | 1.3E-02 (8.1E-03, 1.9E-02) |
| Suriname | 38,173,189 (27,546,600, 45,177,808) | 2.6E-03 (1.9E-03, 3.1E-03) | 50,405,363 (36,757,439, 64,859,401) | 3.4E-03 (2.5E-03, 4.4E-03) |
| Sweden | 9,394,861,999 (8,855,978,106, 9,872,592,886) | 1.6E-03 (1.5E-03, 1.7E-03) | 10,771,801,972 (9,462,557,866, 12,085,311,749) | 1.9E-03 (1.6E-03, 2.1E-03) |
| Switzerland | 9,064,226,986 (8,503,456,077, 9,666,354,943) | 1.2E-02 (1.1E-02, 1.3E-02) | 10,383,858,299 (9,216,471,641, 11,533,559,112) | 1.4E-02 (1.2E-02, 1.6E-02) |
| Syrian Arab Republic | NA | NA | 57,685,252 (41,403,303, 79,648,527) | NA |
| Tajikistan | 121,448,477 (87,075,564, 155,065,382) | 3.6E-01 (2.6E-01, 4.6E-01) | 80,855,196 (54,616,279, 120,522,824) | 2.4E-01 (1.6E-01, 3.5E-01) |
| Thailand | 2,652,405,062 (2,091,544,051, 3,350,808,587) | 2.5E-04 (2.0E-04, 3.2E-04) | 7,974,837,286 (5,210,647,670, 11,081,012,704) | 7.7E-04 (5.0E-04, 1.1E-03) |
| Timor-Leste | 7,989,090 (4,338,800, 12,030,018) | 5.2E-03 (2.8E-03, 7.9E-03) | 29,532,745 (21,652,574, 40,175,012) | 1.9E-02 (1.4E-02, 2.6E-02) |
| Togo | 10,031,435 (7,406,194, 13,174,149) | 2.3E-06 (1.7E-06, 3.0E-06) | 17,434,277 (10,328,984, 24,815,243) | 3.9E-06 (2.3E-06, 5.6E-06) |
| Tonga | 1,212,616 (913,625, 1,539,080) | 1.2E-03 (9.1E-04, 1.5E-03) | 1,951,811 (1,388,312, 2,691,852) | 1.9E-03 (1.4E-03, 2.7E-03) |
| Trinidad and Tobago | 132,187,608 (124,806,885, 140,405,773) | 8.9E-04 (8.4E-04, 9.5E-04) | 544,124,536 (414,991,472, 699,502,267) | 3.7E-03 (2.8E-03, 4.7E-03) |
| Tunisia | 261,254,538 (200,658,985, 328,151,252) | 2.8E-03 (2.2E-03, 3.5E-03) | 467,757,604 (322,531,666, 652,857,283) | 5.1E-03 (3.5E-03, 7.1E-03) |
| Turkey | 12,082,521,094 (8,817,227,305, 15,853,032,832) | 6.0E-03 (4.4E-03, 7.9E-03) | 18,627,840,440 (14,080,603,044, 23,771,218,057) | 9.3E-03 (7.0E-03, 1.2E-02) |
| Tuvalu | 263,732 (185,744, 373,512) | 4.1E-03 (2.9E-03, 5.7E-03) | 205,580 (144,638, 268,528) | 3.2E-03 (2.2E-03, 4.1E-03) |
| Uganda | 60,169,402 (43,883,442, 79,048,120) | 4.5E-07 (3.3E-07, 5.9E-07) | 273,556,940 (179,693,266, 400,632,080) | 2.1E-06 (1.3E-06, 3.0E-06) |
| Ukraine | 10,481,445,237 (9,600,935,072, 11,667,438,258) | 4.0E-03 (3.7E-03, 4.5E-03) | 5,172,401,713 (3,802,681,832, 6,745,474,849) | 2.0E-03 (1.5E-03, 2.6E-03) |
| United Arab Emirates | 4,145,253,798 (2,881,498,954, 5,805,047,281) | 2.8E-03 (1.9E-03, 3.9E-03) | 4,203,980,078 (2,988,990,968, 5,695,837,175) | 2.8E-03 (2.0E-03, 3.8E-03) |
| United Kingdom | 52,478,484,850 (50,973,629,835, 53,545,027,185) | 2.4E-02 (2.3E-02, 2.5E-02) | 64,131,959,596 (60,031,750,098, 66,939,643,738) | 2.9E-02 (2.8E-02, 3.1E-02) |
| United Republic of Tanzania | 136,694,103 (99,506,330, 176,823,756) | 1.0E-06 (7.4E-07, 1.3E-06) | 387,765,435 (256,078,507, 560,877,033) | 2.9E-06 (1.9E-06, 4.2E-06) |
| United States of America | 339,837,000,000 (327,851,000,000, 348,714,000,000) | 1.6E-02 (1.5E-02, 1.6E-02) | 417,422,000,000 (389,485,000,000, 435,803,000,000) | 1.9E-02 (1.8E-02, 2.0E-02) |
| Uruguay | 512,069,173 (481,054,349, 547,214,169) | 2.9E-04 (2.8E-04, 3.1E-04) | 1,710,803,403 (1,573,638,539, 1,859,913,566) | 9.8E-04 (9.0E-04, 1.1E-03) |
| Uzbekistan | 454,442,922 (396,238,955, 522,434,200) | 7.0E-07 (6.1E-07, 8.0E-07) | 597,970,617 (484,980,584, 734,883,578) | 9.2E-07 (7.5E-07, 1.1E-06) |
| Vanuatu | 810,652 (504,874, 1,144,404) | 1.2E-05 (7.6E-06, 1.7E-05) | 1,291,427 (878,107, 1,720,169) | 2.0E-05 (1.3E-05, 2.6E-05) |
| Venezuela (Bolivarian Republic of) | 2,890,811,524 (2,760,595,595, 3,021,714,800) | NA | 3,339,664,938 (2,481,362,895, 4,387,250,801) | NA |
| Viet Nam | 265,539,776 (204,128,190, 347,977,944) | 5.2E-08 (4.0E-08, 6.8E-08) | 2,949,430,490 (2,192,675,463, 4,044,374,029) | 5.7E-07 (4.3E-07, 7.9E-07) |
| Yemen | 162,969,952 (92,254,840, 275,530,701) | 7.7E-04 (4.4E-04, 1.3E-03) | 1,207,989,810 (687,330,727, 1,768,061,394) | 5.7E-03 (3.3E-03, 8.4E-03) |
| Zambia | 92,618,695 (67,680,149, 121,320,959) | 6.3E-04 (4.6E-04, 8.3E-04) | 138,409,055 (79,601,124, 235,685,371) | 9.5E-04 (5.4E-04, 1.6E-03) |
| Zimbabwe | 72,529,794 (50,561,701, 91,250,832) | 3.8E-03 (2.7E-03, 4.8E-03) | 141,544,992 (91,383,190, 191,111,524) | 7.5E-03 (4.8E-03, 1.0E-02) |

Note: GDP, Gross Domestic Product

# Table S2 Projected Economic Burden of Hematologic Malignancies in Various Countries, 2030 (Discounted to 2021 US$)

| Country | Hematologic Malignancies Economic Burden ($) | Hodgkin lymphoma Economic Burden ($) | Leukemia Economic Burden ($) | Multiple myeloma Economic Burden ($) | Non-Hodgkin lymphoma Economic Burden ($) |
| --- | --- | --- | --- | --- | --- |
| Afghanistan | 437,719,840 (330,697,418, 570,622,311) | 32,356,551 (24,590,979, 42,061,881) | 295,643,048 (224,688,717, 388,786,322) | 16,178,365 (12,295,558, 21,812,522) | 93,541,875 (69,122,165, 117,961,586) |
| Albania | 97,025,161 (66,559,090, 153,558,614) | 7,283,787 (5,317,164, 11,268,808) | 59,356,247 (45,110,748, 96,025,074) | 10,559,317 (8,025,081, 14,719,209) | 19,825,810 (8,106,097, 31,545,523) |
| Algeria | 1,179,225,601 (875,629,100, 1,411,390,265) | 120,538,799 (84,377,159, 145,905,835) | 433,382,495 (303,367,746, 516,668,393) | 162,159,688 (113,511,782, 196,899,214) | 463,144,619 (374,372,413, 551,916,824) |
| Angola | 476,733,497 (344,487,615, 624,902,253) | 26,859,246 (19,607,249, 34,523,692) | 172,247,002 (130,907,721, 226,817,440) | 39,601,523 (28,909,112, 52,573,201) | 238,025,726 (165,063,532, 310,987,920) |
| Antigua and Barbuda | 15,994,764 (10,058,139, 25,175,953) | 190 (139, 6,877) | 6,618,184 (4,831,275, 10,155,443) | 4,180,014 (3,051,410, 6,796,196) | 5,196,376 (2,175,315, 8,217,437) |
| Argentina | 4,767,627,598 (3,362,790,880, 7,076,664,239) | 184,985,132 (135,039,147, 488,274,137) | 2,219,411,606 (1,686,752,821, 3,312,094,417) | 671,434,570 (510,290,273, 923,411,745) | 1,691,796,290 (1,030,708,639, 2,352,883,940) |
| Armenia | 253,460,846 (195,087,342, 561,275,746) | 10,036,424 (7,627,682, 23,240,812) | 140,879,641 (107,068,527, 317,195,535) | 41,117,296 (31,249,145, 78,715,372) | 61,427,484 (49,141,988, 142,124,028) |
| Australia | 13,886,182,932 (6,395,924,606, 20,846,247,585) | 346,929,973 (263,666,780, 525,388,287) | 5,513,925,455 (612,274,318, 8,534,742,637) | 3,094,934,272 (2,135,504,648, 5,309,809,057) | 4,930,393,232 (3,384,478,860, 6,476,307,604) |
| Austria | 4,059,944,405 (3,322,211,389, 5,821,729,953) | 152,198,249 (780,706,917, 278,004,219) | 1,765,267,737 (1,218,034,738, 2,538,795,280) | 771,346,143 (532,228,839, 1,053,906,797) | 1,371,132,276 (791,240,895, 1,951,023,657) |
| Azerbaijan | 536,762,722 (340,427,360, 905,490,980) | 48,114,424 (36,566,962, 81,422,939) | 375,283,803 (285,215,690, 605,590,351) | 20,171,897 (15,330,642, 35,406,560) | 93,192,598 (3,314,066, 183,071,130) |
| Bahamas | 87,434,072 (59,248,069, 119,963,256) | 2,478,559 (1,734,991, 4,241,326) | 26,018,639 (18,213,047, 35,769,232) | 27,320,903 (19,124,632, 36,896,152) | 31,615,972 (20,175,398, 43,056,545) |
| Bahrain | 630,606,291 (405,801,160, 894,527,759) | 32,052,816 (22,436,971, 49,789,916) | 303,657,006 (212,559,904, 416,876,260) | 105,303,250 (73,712,275, 145,767,152) | 189,593,221 (97,092,010, 282,094,431) |
| Bangladesh | 5,562,118,651 (3,726,280,868, 7,694,407,044) | 444,534,454 (324,510,152, 586,710,600) | 2,738,286,669 (1,998,949,268, 3,731,965,833) | 655,730,506 (478,683,269, 852,734,746) | 1,723,567,022 (924,138,180, 2,522,995,865) |
| Barbados | 37,066,464 (24,572,093, 53,625,301) | 842,600 (615,098, 1,550,455) | 14,449,791 (10,548,348, 21,033,506) | 5,226,928 (3,815,658, 7,540,041) | 16,547,145 (9,592,990, 23,501,299) |
| Belarus | 2,470,206,179 (1,166,594,309, 4,367,252,330) | 167,962,692 (115,894,258, 303,921,143) | 1,141,062,438 (787,333,082, 1,865,274,964) | 352,001,928 (242,881,330, 600,183,620) | 809,179,121 (20,485,639, 1,597,872,603) |
| Belgium | 7,145,646,176 (4,815,979,685, 11,224,417,438) | 193,693,199 (147,206,831, 278,672,108) | 3,447,396,202 (2,516,599,227, 5,995,113,749) | 1,304,116,169 (991,128,288, 1,710,795,707) | 2,200,440,606 (1,161,045,338, 3,239,835,874) |
| Belize | 8,574,256 (5,790,440, 12,733,807) | 550,472 (401,844, 948,054) | 4,364,127 (3,185,813, 6,315,965) | 958,032 (699,364, 1,569,957) | 2,701,625 (1,503,419, 3,899,831) |
| Benin | 71,950,834 (51,286,855, 93,773,625) | 1,871,201 (1,309,841, 2,883,413) | 27,481,777 (19,237,244, 36,155,809) | 1,375,335 (962,735, 2,066,398) | 41,222,521 (29,777,036, 52,668,006) |
| Bermuda | 57,880,513 (35,590,534, 95,814,933) | 8,564 (6,509, 123,302) | 20,597,256 (15,653,915, 34,105,522) | 16,321,629 (12,404,438, 27,205,654) | 20,953,064 (7,525,673, 34,380,455) |
| Bhutan | 62,519,945 (41,905,161, 87,057,878) | 4,206,358 (2,944,451, 6,204,514) | 28,707,145 (20,095,001, 39,007,324) | 9,328,282 (6,529,797, 13,625,632) | 20,278,160 (12,335,912, 28,220,408) |
| Bolivia (Plurinational State of) | 1,059,769,318 (814,275,013, 1,271,836,809) | 36,281,035 (27,573,587, 46,330,832) | 525,994,964 (399,756,172, 616,575,991) | 104,341,801 (79,299,769, 130,272,435) | 393,151,518 (307,645,485, 478,657,551) |
| Bosnia and Herzegovina | 272,547,828 (170,029,990, 423,936,690) | 15,812,828 (12,017,749, 26,577,851) | 125,608,354 (95,462,349, 191,248,451) | 50,780,812 (38,593,417, 69,375,195) | 80,345,834 (23,956,475, 136,735,194) |
| Botswana | 172,547,385 (81,082,678, 289,284,887) | 6,921,070 (4,844,749, 11,336,270) | 62,719,901 (43,903,931, 98,681,104) | 32,452,341 (22,716,639, 47,976,727) | 70,454,073 (9,617,360, 131,290,786) |
| Brazil | 12,636,377,206 (8,877,666,185, 16,865,204,965) | 466,129,653 (326,290,757, 748,358,669) | 5,814,919,806 (4,070,443,864, 7,289,426,978) | 2,300,998,275 (1,610,698,792, 3,588,993,146) | 4,054,329,472 (2,870,232,772, 5,238,426,172) |
| Brunei Darussalam | 323,753,188 (215,164,717, 467,173,474) | 10,213,420 (7,762,200, 17,333,369) | 128,740,725 (97,842,951, 179,317,734) | 44,409,994 (33,751,595, 65,552,244) | 140,389,049 (75,807,970, 204,970,127) |
| Bulgaria | 1,867,004,300 (1,179,277,814, 3,829,197,079) | 113,185,101 (78,097,719, 196,127,467) | 980,077,276 (676,253,320, 2,483,735,764) | 239,831,838 (165,483,968, 340,956,483) | 533,910,086 (259,442,806, 808,377,365) |
| Burkina Faso | 83,922,640 (54,827,519, 118,577,701) | 2,657,758 (1,860,431, 4,196,389) | 30,660,745 (21,462,522, 44,435,299) | 1,767,951 (1,237,566, 2,540,641) | 48,836,186 (30,267,001, 67,405,372) |
| Burundi | 16,856,656 (11,643,027, 21,936,350) | 2,039,689 (1,407,385, 2,567,305) | 4,855,720 (3,399,004, 6,172,224) | 1,281,859 (897,301, 1,777,382) | 8,679,388 (5,939,337, 11,419,440) |
| Cabo Verde | 5,982,676 (4,462,372, 10,345,176) | NA | 2,958,645 (2,071,052, 4,095,243) | 279,041 (195,329, 571,946) | 2,744,989 (2,195,991, 5,677,980) |
| Cambodia | 416,133,094 (298,750,641, 503,779,631) | 9,749,488 (6,824,642, 12,608,353) | 255,585,690 (178,909,983, 302,366,173) | 16,409,760 (11,486,832, 21,557,980) | 134,388,155 (101,529,184, 167,247,127) |
| Cameroon | 280,499,921 (213,552,252, 354,330,005) | 6,338,883 (4,817,551, 9,124,229) | 84,308,178 (64,074,215, 109,144,410) | 6,897,438 (5,242,053, 9,568,955) | 182,955,423 (139,418,433, 226,492,412) |
| Canada | 15,813,848,368 (11,318,209,815, 20,554,020,013) | 396,954,228 (277,867,960, 547,622,926) | 6,191,667,233 (4,519,917,080, 8,055,121,784) | 3,027,458,953 (2,210,045,036, 3,866,119,133) | 6,197,767,954 (4,310,379,739, 8,085,156,170) |
| Central African Republic | 5,289,082 (3,904,678, 6,561,144) | 436,102 (305,272, 569,373) | 2,027,321 (1,419,124, 2,499,712) | 216,270 (151,389, 302,174) | 2,609,389 (2,028,892, 3,189,886) |
| Chad | 23,139,973 (16,921,176, 29,818,468) | 871,688 (636,332, 1,257,617) | 9,343,053 (6,820,428, 12,082,997) | 396,067 (289,129, 594,808) | 12,529,166 (9,175,287, 15,883,045) |
| Chile | 3,530,573,739 (2,440,253,308, 5,723,959,612) | 168,303,352 (122,861,447, 848,188,299) | 1,440,978,461 (1,051,914,277, 2,019,665,869) | 730,228,790 (533,067,017, 1,206,389,740) | 1,191,063,135 (732,410,567, 1,649,715,704) |
| China | 175,902,004,325 (114,989,501,269, 249,422,137,346) | 3,048,797,231 (2,317,085,896, 4,325,666,118) | 100,471,000,000 (73,343,743,218, 125,809,000,000) | 15,822,400,693 (12,025,024,527, 33,471,506,054) | 56,559,806,401 (27,303,647,628, 85,815,965,174) |
| Colombia | 5,081,839,909 (3,190,114,763, 7,982,771,939) | 191,555,072 (134,088,551, 529,241,763) | 2,374,595,574 (1,662,216,902, 3,591,285,428) | 872,323,084 (610,626,159, 1,358,695,543) | 1,643,366,178 (783,183,151, 2,503,549,205) |
| Comoros | 8,403,293 (4,008,450, 14,530,889) | 866,600 (606,620, 1,447,164) | 2,458,134 (1,720,694, 4,473,323) | 834,408 (584,086, 1,219,152) | 4,244,150 (1,097,050, 7,391,251) |
| Congo | 57,602,016 (38,121,746, 80,548,337) | 2,788,711 (2,035,759, 3,810,732) | 18,553,281 (14,100,493, 25,531,797) | 5,029,099 (3,822,115, 6,907,336) | 31,230,925 (18,163,378, 44,298,473) |
| Costa Rica | 1,329,065,985 (752,556,182, 2,570,808,120) | 50,649,794 (35,454,855, 75,062,398) | 633,893,258 (443,725,281, 1,394,092,742) | 207,245,131 (145,071,592, 355,401,829) | 437,277,803 (128,304,455, 746,251,151) |
| Coted' Ivoire | 932,427,905 (578,264,764, 1,353,037,699) | 62,222,680 (47,289,237, 95,463,037) | 130,948,039 (99,520,509, 201,900,011) | 157,948,475 (120,040,841, 204,471,404) | 581,308,712 (311,414,177, 851,203,247) |
| Croatia | 1,688,357,547 (1,274,103,644, 3,202,699,056) | 41,415,534 (30,233,340, 127,038,573) | 723,353,872 (528,048,326, 1,162,260,024) | 329,264,794 (240,363,299, 560,327,141) | 594,323,348 (475,458,678, 1,353,073,319) |
| Cyprus | 370,542,833 (265,503,978, 490,118,198) | 13,117,978 (9,576,124, 19,967,462) | 161,723,950 (118,058,483, 211,208,028) | 69,435,523 (50,687,932, 93,593,382) | 126,265,383 (87,181,439, 165,349,326) |
| Czechia | 5,548,422,484 (3,812,342,430, 7,674,195,214) | 195,495,558 (134,891,935, 256,633,988) | 2,414,101,567 (1,665,730,081, 3,399,171,573) | 1,055,730,219 (728,453,851, 1,535,465,935) | 1,883,095,140 (1,283,266,563, 2,482,923,718) |
| Democratic Republic of the Congo | 225,929,815 (168,505,032, 280,438,816) | 15,844,747 (11,566,666, 19,665,283) | 81,557,468 (59,536,952, 100,126,253) | 12,815,661 (9,355,433, 17,269,385) | 115,711,939 (88,045,982, 143,377,896) |
| Denmark | 4,166,328,709 (2,313,582,759, 7,312,261,828) | 62,971,938 (47,858,673, 104,508,029) | 1,980,030,785 (1,504,823,396, 3,080,225,270) | 889,088,968 (675,707,616, 1,744,247,568) | 1,234,237,017 (85,193,074, 2,383,280,961) |
| Dominica | 9,000,225 (5,618,064, 13,667,466) | 222,613 (155,829, 427,200) | 3,800,337 (2,660,236, 5,649,577) | 1,208,645 (846,052, 2,009,376) | 3,768,630 (1,955,947, 5,581,312) |
| Dominican Republic | 1,212,918,515 (804,812,931, 1,660,500,185) | 8,955,145 (6,268,601, 13,668,112) | 608,595,133 (444,274,447, 802,258,549) | 239,664,201 (174,954,867, 312,480,469) | 355,704,036 (179,315,016, 532,093,055) |
| Ecuador | 946,970,580 (430,345,580, 2,127,328,458) | 38,802,994 (29,490,275, 159,892,382) | 457,198,232 (315,466,780, 950,823,490) | 110,979,010 (76,575,517, 345,444,905) | 339,990,344 (8,813,007, 671,167,681) |
| Egypt | 10,127,112,276 (7,304,786,960, 15,263,731,593) | 111,858,431 (85,012,408, 158,244,841) | 7,593,295,750 (5,770,904,770, 11,704,957,560) | 407,534,071 (309,725,894, 510,825,033) | 2,014,424,024 (1,139,143,888, 2,889,704,159) |
| El Salvador | 268,928,560 (201,307,737, 455,297,345) | 10,743,103 (7,842,465, 15,191,122) | 170,164,168 (124,219,843, 270,868,517) | 16,737,175 (12,218,138, 22,237,047) | 71,284,114 (57,027,291, 147,000,659) |
| Equatorial Guinea | 42,596,341 (26,951,808, 62,116,251) | 1,216,737 (924,720, 1,946,553) | 13,209,697 (10,039,370, 18,507,093) | 5,371,417 (4,082,277, 7,971,067) | 22,798,490 (11,905,441, 33,691,538) |
| Estonia | 919,115,206 (695,692,336, 1,706,740,180) | 20,005,684 (14,604,150, 34,729,642) | 328,802,614 (240,025,908, 524,068,106) | 216,903,549 (158,339,591, 389,865,087) | 353,403,359 (282,722,687, 758,077,346) |
| Eswatini | 50,752,655 (28,211,338, 79,206,460) | 2,187,890 (1,662,797, 3,498,872) | 16,335,636 (11,925,014, 24,087,052) | 8,136,635 (5,939,743, 12,119,332) | 24,092,494 (8,683,784, 39,501,204) |
| Ethiopia | 3,021,582,551 (2,241,969,408, 3,714,091,518) | 261,271,206 (198,566,116, 325,487,678) | 1,639,957,911 (1,197,169,275, 1,987,480,002) | 132,960,301 (101,049,829, 171,521,758) | 987,393,134 (745,184,188, 1,229,602,080) |
| Fiji | 46,598,560 (33,023,194, 64,310,276) | 1,466,414 (1,114,475, 2,746,512) | 31,475,320 (23,921,243, 41,367,634) | 3,519,380 (2,674,729, 5,233,985) | 10,137,446 (5,312,748, 14,962,145) |
| Finland | 2,875,898,638 (2,053,150,664, 4,044,636,401) | 65,457,190 (49,747,465, 107,417,382) | 1,047,554,070 (764,714,471, 1,583,431,296) | 563,641,189 (428,367,304, 765,616,770) | 1,199,246,189 (810,321,424, 1,588,170,953) |
| France | 30,665,271,579 (20,204,593,746, 42,249,891,908) | 892,019,802 (624,413,862, 1,287,909,770) | 13,748,391,150 (9,623,873,805, 17,826,725,934) | 5,761,530,026 (4,033,071,018, 8,531,830,063) | 10,263,330,601 (5,923,235,061, 14,603,426,141) |
| Gabon | 108,190,997 (79,532,383, 141,692,876) | 3,896,221 (2,844,242, 5,696,001) | 31,119,553 (23,650,860, 40,520,417) | 15,486,677 (11,769,875, 21,366,773) | 57,688,545 (41,267,407, 74,109,684) |
| Gambia | 6,817,955 (2,365,722, 11,757,312) | 699,709 (489,796, 1,068,862) | 1,063,652 (744,557, 1,653,572) | 144,833 (101,383, 245,342) | 4,909,760 (1,029,985, 8,789,535) |
| Georgia | 573,256,804 (412,998,852, 1,706,300,630) | 63,313,006 (43,685,974, 205,548,577) | 268,361,287 (185,169,288, 564,662,737) | 82,931,085 (57,222,448, 401,491,623) | 158,651,427 (126,921,142, 534,597,693) |
| Germany | 51,414,556,069 (39,751,233,953, 96,898,361,678) | 1,451,877,903 (1,103,427,207, 2,916,351,279) | 24,119,941,063 (18,331,155,208, 32,205,928,803) | 8,938,453,627 (6,793,224,757, 12,901,875,559) | 16,904,283,476 (13,523,426,781, 48,874,206,037) |
| Ghana | 515,689,482 (141,823,603, 967,375,936) | 1,875,294 (1,312,706, 3,838,552) | 151,689,352 (106,182,547, 272,524,131) | 13,303,089 (9,312,162, 18,385,946) | 348,821,748 (25,016,188, 672,627,307) |
| Greece | 2,820,970,294 (1,619,453,489, 4,360,655,127) | 245,228,773 (179,017,005, 524,666,440) | 1,430,376,829 (1,001,263,780, 1,971,318,860) | 505,239,400 (353,667,580, 669,924,369) | 640,125,291 (85,505,124, 1,194,745,458) |
| Grenada | 12,698,877 (8,062,957, 18,759,150) | 145,855 (102,098, 310,647) | 3,468,329 (2,427,830, 5,203,855) | 2,175,558 (1,522,891, 3,436,516) | 6,909,135 (4,010,138, 9,808,132) |
| Guatemala | 826,347,417 (595,474,299, 1,662,227,569) | 24,786,085 (17,350,259, 35,470,276) | 569,724,741 (398,807,319, 1,174,579,743) | 61,525,516 (43,067,861, 80,796,283) | 170,311,075 (136,248,860, 371,381,267) |
| Guinea | 72,101,407 (52,288,752, 92,271,587) | 11,779,640 (8,245,748, 14,895,663) | 10,247,392 (7,173,175, 13,803,368) | 4,016,824 (2,811,777, 5,515,508) | 46,057,550 (34,058,053, 58,057,048) |
| Guinea-Bissau | 8,987,844 (6,454,363, 11,964,043) | 285,102 (208,125, 433,001) | 2,801,921 (2,045,402, 3,804,525) | 216,066 (157,728, 400,117) | 5,684,754 (4,043,108, 7,326,401) |
| Guyana | 129,033,206 (80,015,846, 244,339,503) | 7,925,894 (5,785,903, 12,281,456) | 58,113,353 (42,422,748, 116,858,519) | 15,948,309 (11,642,265, 41,273,158) | 47,045,650 (20,164,930, 73,926,370) |
| Haiti | 130,336,400 (92,935,770, 165,900,164) | 7,682,709 (5,377,897, 9,831,680) | 67,070,807 (46,949,565, 85,872,461) | 9,055,380 (6,338,766, 11,410,558) | 46,527,504 (34,269,542, 58,785,465) |
| Honduras | 171,369,666 (121,362,447, 213,336,561) | 4,892,289 (3,424,603, 6,581,783) | 112,105,862 (78,474,104, 137,813,714) | 20,839,429 (14,587,600, 26,753,034) | 33,532,085 (24,876,140, 42,188,031) |
| Hungary | 3,958,506,374 (2,568,757,767, 5,623,349,005) | 110,933,618 (84,309,550, 183,285,215) | 1,988,774,213 (1,511,468,402, 2,512,283,320) | 645,238,179 (490,381,016, 983,258,541) | 1,213,560,364 (482,598,800, 1,944,521,929) |
| Iceland | 231,004,633 (162,057,425, 323,620,517) | 5,457,713 (4,147,862, 10,207,205) | 89,376,587 (67,926,206, 122,306,878) | 47,391,832 (36,017,792, 67,514,997) | 88,778,501 (53,965,565, 123,591,437) |
| India | 36,946,121,310 (26,277,447,086, 47,054,138,780) | 2,321,380,154 (1,694,607,513, 2,923,521,943) | 16,347,697,808 (11,933,819,400, 20,338,856,985) | 5,595,362,999 (4,084,614,989, 6,992,804,338) | 12,681,680,349 (8,564,405,184, 16,798,955,514) |
| Indonesia | 14,051,706,636 (10,662,705,156, 16,329,061,419) | 325,233,515 (247,177,471, 380,745,456) | 9,037,523,766 (6,597,392,349, 10,447,411,213) | 657,188,465 (479,747,579, 775,770,726) | 4,031,760,890 (3,338,387,756, 4,725,134,024) |
| Iran (Islamic Republic of) | 1,825,853,940 (1,264,593,963, 2,358,960,379) | 67,497,806 (47,248,464, 122,130,056) | 1,199,858,243 (839,900,770, 1,487,170,768) | 151,776,993 (106,243,895, 207,418,594) | 406,720,898 (271,200,834, 542,240,962) |
| Iraq | 1,765,882,436 (1,232,662,347, 2,235,722,645) | 62,611,211 (43,827,848, 92,316,401) | 978,278,043 (684,794,630, 1,206,688,858) | 209,899,199 (146,929,440, 263,639,849) | 515,093,983 (357,110,430, 673,077,537) |
| Ireland | 3,337,033,853 (1,964,372,984, 5,150,918,322) | 140,200,853 (102,346,623, 212,575,685) | 1,148,275,426 (838,241,061, 1,728,328,439) | 853,937,854 (623,374,634, 1,221,185,427) | 1,194,619,719 (400,410,666, 1,988,828,771) |
| Israel | 3,384,314,036 (1,951,775,766, 5,389,857,255) | 89,964,486 (65,674,075, 120,636,081) | 1,241,148,493 (856,392,460, 1,977,447,860) | 700,129,401 (511,094,463, 1,104,244,771) | 1,353,071,656 (518,614,768, 2,187,528,543) |
| Italy | 25,019,494,087 (16,826,570,739, 36,204,236,711) | 1,235,619,425 (939,070,763, 2,664,654,541) | 10,209,354,254 (7,759,109,233, 13,308,091,231) | 4,975,083,804 (3,781,063,691, 7,379,944,782) | 8,599,436,604 (4,347,327,052, 12,851,546,157) |
| Jamaica | 104,672,712 (79,660,442, 455,514,927) | 1,954,188 (1,426,557, 3,613,719) | 33,050,940 (24,127,186, 97,865,650) | 23,248,117 (16,971,125, 119,217,452) | 46,419,467 (37,135,573, 234,818,106) |
| Japan | 32,695,130,294 (23,018,182,817, 41,724,256,305) | 437,520,251 (306,264,176, 1,472,105,812) | 13,959,723,179 (9,771,806,226, 16,839,991,309) | 4,243,298,153 (2,970,308,707, 5,272,785,469) | 14,054,588,711 (9,969,803,708, 18,139,373,715) |
| Jordan | 260,859,479 (176,523,756, 364,653,609) | 4,417,687 (3,224,911, 6,724,505) | 139,587,443 (101,898,834, 194,118,260) | 19,387,859 (14,153,137, 26,124,739) | 97,466,490 (57,246,874, 137,686,106) |
| Kazakhstan | 2,655,389,845 (2,046,372,426, 5,925,513,832) | 164,510,357 (125,027,871, 326,852,788) | 1,450,680,060 (1,102,516,846, 2,538,354,460) | 333,295,853 (253,304,849, 726,167,643) | 706,903,575 (565,522,860, 2,334,138,941) |
| Kenya | 1,114,869,972 (761,933,468, 1,468,513,298) | 70,028,445 (49,019,911, 94,569,705) | 340,760,453 (238,532,317, 449,260,016) | 195,330,838 (136,731,586, 244,832,757) | 508,750,236 (337,649,653, 679,850,820) |
| Kiribati | 1,508,470 (962,753, 2,355,473) | NA | 1,222,313 (892,289, 1,794,976) | 52,812 (38,553, 125,709) | 233,345 (31,912, 434,778) |
| Kuwait | 666,805,665 (505,944,012, 2,899,969,591) | 18,047,657 (13,174,790, 198,098,316) | 309,221,835 (225,731,939, 653,277,722) | 65,595,072 (47,884,402, 203,637,395) | 273,941,102 (219,152,881, 1,844,956,158) |
| Kyrgyzstan | 91,412,080 (65,284,479, 195,138,221) | 5,810,665 (4,009,359, 25,875,756) | 58,773,307 (40,553,582, 102,564,400) | 6,735,891 (4,647,765, 13,462,606) | 20,092,217 (16,073,773, 53,235,460) |
| Lao People's Democratic Republic | 404,797,252 (293,404,985, 490,453,389) | 9,814,410 (7,164,519, 13,160,631) | 257,711,163 (180,397,814, 307,271,784) | 16,541,465 (12,075,269, 22,327,928) | 120,730,215 (93,767,383, 147,693,047) |
| Latvia | 965,951,739 (506,928,076, 1,901,700,911) | 36,730,094 (27,914,871, 60,213,849) | 384,232,908 (292,017,010, 821,689,835) | 220,117,079 (167,288,980, 389,761,124) | 324,871,659 (19,707,215, 630,036,102) |
| Lebanon | 457,062,415 (275,846,931, 643,101,331) | 21,335,955 (16,215,326, 28,372,477) | 159,053,794 (116,109,269, 196,528,482) | 66,208,663 (50,318,584, 90,476,117) | 210,464,004 (93,203,753, 327,724,255) |
| Lesotho | 14,391,224 (7,236,052, 23,592,096) | 773,067 (541,147, 1,282,180) | 5,281,552 (3,697,086, 8,354,729) | 1,733,886 (1,213,720, 2,533,846) | 6,602,720 (1,784,099, 11,421,340) |
| Liberia | 14,768,850 (8,054,535, 23,348,045) | 358,034 (250,624, 548,451) | 4,832,775 (3,382,943, 7,975,030) | 249,408 (174,585, 413,681) | 9,328,633 (4,246,383, 14,410,883) |
| Libya | 308,937,210 (189,633,032, 452,680,152) | 26,987,453 (18,891,217, 37,063,312) | 135,038,903 (94,527,232, 196,849,126) | 29,445,248 (20,611,674, 39,439,411) | 117,465,606 (55,602,909, 179,328,304) |
| Lithuania | 1,920,219,861 (1,445,345,187, 3,642,668,607) | 74,771,747 (54,583,376, 156,812,241) | 809,048,101 (590,605,114, 1,343,044,267) | 413,761,614 (302,045,978, 669,708,204) | 622,638,399 (498,110,719, 1,473,103,895) |
| Luxembourg | 523,033,569 (370,599,320, 713,798,206) | 9,619,449 (7,310,782, 17,162,075) | 245,440,884 (186,535,072, 320,346,844) | 107,764,814 (81,901,259, 150,724,652) | 160,208,421 (94,852,207, 225,564,635) |
| Madagascar | 60,922,987 (46,502,355, 73,880,888) | 6,660,884 (4,862,446, 8,081,886) | 17,891,647 (13,060,902, 21,667,617) | 5,420,078 (3,956,657, 6,852,979) | 30,950,378 (24,622,351, 37,278,406) |
| Malawi | 81,901,489 (61,265,651, 102,103,450) | 2,963,179 (2,074,225, 3,672,428) | 5,013,040 (3,509,128, 6,267,929) | 2,840,474 (1,988,332, 3,687,466) | 71,084,796 (53,693,966, 88,475,626) |
| Malaysia | 9,212,840,317 (6,984,683,279, 19,594,277,969) | 205,767,807 (150,210,499, 277,236,002) | 4,744,547,586 (3,463,519,738, 9,481,012,181) | 558,098,533 (407,411,929, 717,159,670) | 3,704,426,391 (2,963,541,113, 9,118,870,116) |
| Maldives | 12,914,917 (8,684,886, 18,713,214) | 267,399 (195,201, 563,800) | 7,437,175 (5,429,138, 10,178,451) | 1,726,320 (1,260,214, 2,803,252) | 3,484,022 (1,800,333, 5,167,711) |
| Mali | 43,664,906 (30,785,405, 56,876,894) | 9,571,599 (6,700,119, 12,410,305) | 13,226,690 (9,258,683, 17,550,466) | 7,407 (5,185, 19,120) | 20,859,210 (14,821,418, 26,897,003) |
| Malta | 201,962,320 (134,839,256, 287,745,109) | 7,839,051 (5,957,679, 13,335,665) | 86,494,418 (65,735,757, 117,054,063) | 33,195,516 (24,232,727, 47,401,805) | 74,433,335 (38,913,093, 109,953,576) |
| Marshall Islands | 1,777,069 (1,053,110, 2,966,916) | NA | 1,049,122 (797,333, 1,653,765) | 103,776 (78,870, 241,703) | 624,171 (176,908, 1,071,433) |
| Mauritania | 65,369,891 (46,472,129, 85,879,865) | 1,282,565 (936,273, 1,957,624) | 22,091,093 (16,126,498, 28,756,379) | 1,771,081 (1,292,889, 2,832,027) | 40,225,151 (28,116,469, 52,333,834) |
| Mauritius | 202,051,152 (147,933,255, 537,258,532) | 14,351,655 (10,046,159, 37,460,953) | 77,660,580 (54,362,406, 133,569,992) | 45,064,432 (31,545,102, 191,448,048) | 64,974,485 (51,979,588, 174,779,540) |
| Mexico | 14,293,717,629 (9,335,168,541, 19,711,787,516) | 765,212,886 (535,649,020, 1,590,844,628) | 7,552,021,251 (5,286,414,876, 9,076,469,175) | 1,731,189,572 (1,211,832,701, 2,855,157,816) | 4,245,293,920 (2,301,271,944, 6,189,315,897) |
| Micronesia (Federated States of) | 1,059,422 (667,596, 1,613,418) | 19 (14, 508) | 618,505 (451,509, 897,027) | 61,572 (44,948, 128,358) | 379,325 (171,126, 587,525) |
| Mongolia | 164,009,348 (97,889,046, 271,701,488) | 12,156,687 (9,239,082, 17,941,610) | 94,313,831 (71,678,512, 153,237,939) | 11,271,388 (8,566,255, 16,392,252) | 46,267,442 (8,405,197, 84,129,686) |
| Montenegro | 92,696,164 (66,845,040, 130,472,690) | 8,012,926 (6,089,824, 12,878,828) | 44,883,781 (34,111,674, 61,619,386) | 14,567,529 (11,071,322, 21,082,842) | 25,231,927 (15,572,220, 34,891,634) |
| Morocco | 528,642,013 (417,463,026, 634,497,074) | 50,029,626 (38,022,516, 62,468,403) | 102,597,816 (77,974,340, 121,911,639) | 75,084,771 (57,064,426, 92,659,177) | 300,929,799 (244,401,744, 357,457,855) |
| Mozambique | 98,842,329 (72,747,943, 129,877,583) | 12,921,128 (9,820,057, 17,639,501) | 41,061,440 (31,206,695, 53,883,954) | 10,898,169 (8,282,608, 13,869,528) | 33,961,591 (23,438,583, 44,484,600) |
| Myanmar | 1,175,444,721 (871,680,770, 1,442,030,500) | 27,522,397 (19,265,678, 35,603,411) | 754,496,897 (550,782,735, 923,961,013) | 45,629,062 (31,940,343, 56,565,361) | 347,796,365 (269,692,014, 425,900,715) |
| Namibia | 108,443,572 (73,811,009, 144,618,777) | 6,944,917 (4,861,442, 9,571,864) | 30,100,581 (21,070,407, 38,913,046) | 14,882,428 (10,417,699, 20,564,036) | 56,515,646 (37,461,460, 75,569,832) |
| Nepal | 478,634,937 (334,748,140, 614,503,219) | 35,815,424 (26,145,260, 46,445,486) | 222,283,808 (155,598,666, 278,502,421) | 52,806,153 (36,964,307, 70,136,117) | 167,729,552 (116,039,908, 219,419,196) |
| Netherlands | 8,474,269,775 (5,765,473,764, 11,870,274,369) | 295,431,160 (206,801,812, 401,476,228) | 3,262,530,726 (2,283,771,508, 4,749,038,534) | 2,196,302,608 (1,603,300,904, 2,951,348,585) | 2,720,005,281 (1,671,599,540, 3,768,411,022) |
| New Zealand | 2,407,036,162 (1,826,731,066, 5,593,737,724) | 43,009,657 (31,397,049, 179,308,955) | 842,625,727 (615,116,781, 2,055,231,441) | 527,191,242 (384,849,606, 797,078,274) | 994,209,536 (795,367,629, 2,562,119,055) |
| Nicaragua | 73,955,537 (49,302,373, 105,082,030) | 3,474,043 (2,431,830, 4,787,021) | 41,827,443 (29,279,210, 60,159,287) | 6,462,991 (4,524,094, 8,820,841) | 22,191,060 (13,067,238, 31,314,882) |
| Niger | 31,079,390 (19,522,210, 46,139,115) | 1,366,868 (956,807, 2,081,626) | 13,175,244 (9,222,671, 20,234,710) | 485,404 (339,783, 721,978) | 16,051,874 (9,002,948, 23,100,800) |
| Nigeria | 3,377,204,177 (2,491,233,552, 4,252,564,731) | 924,343,909 (674,771,053, 1,187,567,499) | 690,987,448 (504,420,837, 856,650,411) | 304,817,887 (222,517,058, 383,761,558) | 1,457,054,933 (1,089,524,604, 1,824,585,263) |
| North Macedonia | 216,305,627 (159,241,680, 286,577,587) | 19,656,836 (14,939,195, 29,176,588) | 112,809,694 (85,735,368, 145,699,463) | 28,072,521 (21,335,116, 37,400,385) | 55,766,576 (37,232,002, 74,301,151) |
| Norway | 3,855,663,458 (2,106,385,360, 6,235,607,154) | 125,120,592 (87,584,414, 217,088,634) | 1,526,182,573 (1,068,327,801, 2,518,626,265) | 1,042,546,755 (729,782,728, 1,396,955,596) | 1,161,813,538 (220,690,416, 2,102,936,659) |
| Oman | 379,276,373 (233,114,778, 588,118,388) | 22,038,791 (16,088,317, 36,106,775) | 132,803,055 (96,946,230, 209,202,046) | 65,389,602 (47,734,409, 97,065,537) | 159,044,926 (72,345,821, 245,744,030) |
| Pakistan | 5,263,537,871 (4,023,641,463, 6,470,822,133) | 613,054,660 (465,921,541, 791,263,186) | 1,737,958,093 (1,320,848,151, 2,127,885,639) | 601,005,010 (456,763,808, 708,741,055) | 2,311,520,108 (1,780,107,963, 2,842,932,253) |
| Panama | 1,634,417,878 (1,079,070,661, 2,289,668,776) | 34,960,415 (24,472,291, 49,433,808) | 813,341,452 (569,339,017, 1,150,375,951) | 282,919,685 (198,043,780, 370,681,940) | 503,196,326 (287,215,574, 719,177,078) |
| Papua New Guinea | 82,541,941 (62,735,761, 103,113,184) | 1,154,393 (842,707, 1,627,700) | 56,054,933 (42,601,749, 69,787,002) | 2,656,803 (1,939,466, 3,698,697) | 22,675,812 (17,351,839, 27,999,785) |
| Paraguay | 490,291,025 (354,836,774, 642,454,023) | 16,763,512 (12,237,364, 22,197,047) | 264,921,044 (193,392,362, 350,933,080) | 59,283,670 (43,277,079, 76,608,266) | 149,322,800 (105,929,969, 192,715,630) |
| Peru | 2,027,831,239 (1,419,481,868, 4,043,036,579) | 49,389,892 (34,572,924, 80,105,248) | 869,833,451 (608,883,416, 1,670,291,746) | 287,105,435 (200,973,804, 485,483,309) | 821,502,462 (575,051,723, 1,807,156,276) |
| Philippines | 3,801,660,991 (2,833,922,461, 4,614,553,229) | 59,277,069 (43,272,260, 76,570,041) | 2,355,094,367 (1,719,218,888, 2,844,279,133) | 187,443,047 (136,833,424, 228,608,927) | 1,199,846,508 (934,597,888, 1,465,095,128) |
| Poland | 15,546,250,842 (10,775,149,861, 22,315,158,326) | 697,807,766 (530,333,902, 1,697,500,851) | 6,634,368,457 (5,042,120,027, 8,732,407,112) | 3,425,770,974 (2,603,585,940, 4,907,753,065) | 4,788,303,645 (2,599,109,992, 6,977,497,298) |
| Portugal | 3,152,097,073 (1,913,460,713, 5,527,235,816) | 83,448,735 (60,917,577, 129,751,372) | 1,363,864,844 (995,621,336, 2,728,677,633) | 585,454,275 (427,381,621, 859,688,554) | 1,119,329,219 (429,540,180, 1,809,118,257) |
| Puerto Rico | 950,402,852 (735,404,061, 1,766,004,582) | 28,637,977 (20,046,584, 44,804,717) | 363,457,614 (276,227,786, 654,771,307) | 187,902,965 (142,806,254, 308,964,381) | 370,404,297 (296,323,437, 757,464,178) |
| Qatar | 742,146,383 (481,697,554, 1,060,328,713) | 22,490,173 (15,743,121, 35,986,833) | 371,121,812 (259,785,269, 520,336,482) | 120,509,802 (84,356,862, 169,768,510) | 228,024,596 (121,812,303, 334,236,888) |
| Republic of Moldova | 382,625,997 (167,560,721, 669,018,636) | 31,423,471 (21,682,195, 58,149,486) | 155,871,595 (107,551,401, 238,892,847) | 45,327,550 (31,276,010, 79,020,657) | 150,003,381 (7,051,116, 292,955,645) |
| Romania | 7,626,433,574 (5,415,078,469, 10,570,242,642) | 412,379,378 (301,036,946, 884,267,425) | 3,700,205,251 (2,701,149,833, 4,865,532,265) | 1,191,261,642 (869,620,998, 1,718,539,037) | 2,322,587,303 (1,543,270,692, 3,101,903,915) |
| Russian Federation | 46,135,729,743 (29,461,867,147, 71,875,882,553) | 2,784,724,076 (1,949,306,853, 4,880,792,219) | 19,771,473,888 (13,840,031,721, 32,111,035,179) | 6,855,325,141 (4,798,727,599, 10,309,442,853) | 16,724,206,638 (8,873,800,974, 24,574,612,302) |
| Rwanda | 164,432,427 (97,986,284, 239,906,421) | 13,423,899 (9,396,729, 19,081,569) | 44,610,626 (31,227,438, 63,267,585) | 18,878,641 (13,215,048, 26,665,812) | 87,519,262 (44,147,069, 130,891,455) |
| Saint Lucia | 18,153,620 (12,293,546, 26,387,045) | 657,141 (499,427, 1,155,683) | 5,275,450 (4,009,342, 7,575,384) | 4,300,416 (3,268,316, 6,331,213) | 7,920,613 (4,516,460, 11,324,765) |
| Saint Vincent and the Grenadines | 13,242,414 (8,466,494, 19,610,348) | 366,485 (256,540, 743,825) | 4,779,595 (3,345,716, 6,991,153) | 1,626,236 (1,138,365, 2,661,047) | 6,470,098 (3,725,872, 9,214,323) |
| Samoa | 5,486,822 (3,773,683, 7,889,936) | 442,509 (323,032, 727,349) | 2,650,047 (2,014,036, 3,665,174) | 275,646 (209,491, 487,298) | 2,118,620 (1,227,125, 3,010,115) |
| Sao Tome and Principe | 2,074,081 (1,083,007, 3,268,012) | NA | 376,730 (263,711, 633,288) | 50,460 (35,322, 124,903) | 1,646,892 (783,974, 2,509,809) |
| Saudi Arabia | 9,791,057,547 (6,897,092,332, 13,063,985,365) | 334,885,274 (234,419,692, 473,947,103) | 3,791,899,263 (2,768,086,462, 5,197,613,383) | 922,470,080 (645,729,056, 1,157,676,141) | 4,741,802,930 (3,248,857,122, 6,234,748,738) |
| Senegal | 94,506,669 (62,205,667, 142,088,695) | 3,151,599 (2,300,667, 4,272,812) | 31,675,606 (23,123,193, 54,852,925) | 2,259,348 (1,649,324, 3,255,211) | 57,420,115 (35,132,483, 79,707,748) |
| Serbia | 1,160,217,352 (803,912,468, 1,590,730,887) | 64,625,277 (47,176,452, 89,570,951) | 535,826,794 (391,153,560, 738,783,313) | 217,370,123 (158,680,190, 284,488,575) | 342,395,157 (206,902,266, 477,888,048) |
| Seychelles | 41,199,154 (27,020,129, 63,660,122) | 986,092 (749,430, 2,222,820) | 18,931,556 (14,387,982, 28,214,470) | 5,812,162 (4,417,243, 9,749,618) | 15,469,343 (7,465,473, 23,473,214) |
| Sierra Leone | 14,583,828 (10,364,524, 20,055,998) | 416,372 (303,952, 580,505) | 5,345,135 (3,741,594, 8,078,959) | 246,599 (172,619, 391,448) | 8,575,722 (6,146,359, 11,005,086) |
| Singapore | 2,974,820,733 (1,935,546,297, 4,122,157,689) | 56,734,282 (39,713,997, 120,050,720) | 1,411,450,511 (988,015,357, 1,898,937,402) | 258,494,942 (180,946,459, 333,758,053) | 1,248,140,999 (726,870,484, 1,769,411,514) |
| Slovakia | 1,900,562,171 (1,414,050,315, 2,409,597,653) | 97,761,575 (74,298,797, 127,604,145) | 849,878,747 (645,907,848, 1,058,061,372) | 416,201,837 (316,313,396, 528,022,386) | 536,720,013 (377,530,274, 695,909,751) |
| Slovenia | 866,857,479 (465,816,322, 1,409,135,238) | 16,584,529 (12,604,242, 24,727,561) | 294,450,072 (223,782,055, 471,635,456) | 198,223,908 (150,650,170, 276,354,136) | 357,598,970 (78,779,855, 636,418,085) |
| Solomon Islands | 3,349,007 (2,406,107, 4,583,162) | 50,447 (38,339, 95,024) | 2,113,621 (1,606,352, 2,820,936) | 123,629 (93,958, 212,039) | 1,061,310 (667,457, 1,455,163) |
| Somalia | 102,929,166 (71,741,470, 135,728,856) | 15,277,599 (10,694,319, 20,304,312) | 31,055,867 (21,739,107, 41,546,308) | 4,173,845 (2,921,692, 5,420,878) | 52,421,854 (36,386,352, 68,457,357) |
| Spain | 12,633,463,902 (8,619,525,681, 16,842,765,942) | 495,739,055 (347,017,339, 892,472,960) | 5,508,596,313 (3,856,017,419, 6,878,974,516) | 2,380,717,940 (1,666,502,558, 3,324,485,643) | 4,248,410,594 (2,749,988,365, 5,746,832,823) |
| Sri Lanka | 1,686,430,907 (1,226,018,366, 4,339,431,209) | 35,774,373 (25,042,061, 60,246,783) | 1,075,267,989 (752,687,593, 3,031,555,581) | 120,221,239 (84,154,868, 172,766,231) | 455,167,306 (364,133,845, 1,074,862,613) |
| Sudan | 229,566,996 (171,114,392, 271,776,464) | 11,475,336 (8,376,996, 14,488,152) | 147,719,742 (107,835,412, 171,634,189) | 12,759,822 (9,314,670, 16,017,246) | 57,612,096 (45,587,315, 69,636,877) |
| Suriname | 47,074,912 (31,164,084, 65,827,712) | 2,514,882 (1,735,269, 3,840,619) | 19,617,522 (13,536,090, 27,201,264) | 7,504,723 (5,178,259, 10,624,726) | 17,437,785 (10,714,466, 24,161,104) |
| Sweden | 4,092,110,283 (2,738,092,805, 6,617,764,938) | 87,077,291 (66,178,741, 209,024,464) | 1,537,094,494 (1,168,191,815, 2,940,328,579) | 1,075,205,940 (797,856,601, 1,388,812,426) | 1,392,732,558 (705,865,648, 2,079,599,469) |
| Switzerland | 4,521,889,720 (3,312,601,392, 8,810,475,825) | 62,864,835 (45,891,330, 106,205,887) | 2,007,705,763 (1,405,394,034, 3,811,251,727) | 997,392,689 (698,174,882, 1,962,814,785) | 1,453,926,433 (1,163,141,146, 2,930,203,426) |
| Tajikistan | 114,996,662 (68,135,030, 180,760,010) | 2,307,609 (1,753,783, 3,515,224) | 67,947,040 (51,639,751, 101,259,369) | 3,964,240 (3,012,823, 6,158,547) | 40,777,772 (11,728,674, 69,826,871) |
| Thailand | 9,727,898,642 (5,772,267,903, 14,137,846,613) | 115,538,210 (84,342,893, 179,285,586) | 5,784,076,539 (4,222,375,873, 7,672,124,260) | 485,720,521 (354,575,980, 712,283,179) | 3,342,563,373 (1,110,973,157, 5,574,153,588) |
| Timor-Leste | 35,688,979 (23,264,472, 56,019,253) | 1,130,144 (825,005, 1,981,095) | 24,328,724 (17,759,968, 37,932,761) | 988,568 (721,655, 1,580,157) | 9,241,542 (3,957,844, 14,525,240) |
| Togo | 31,191,411 (23,437,287, 39,828,441) | 871,634 (636,293, 1,283,829) | 10,610,503 (7,745,667, 14,010,478) | 602,910 (440,124, 936,610) | 19,106,364 (14,615,203, 23,597,524) |
| Tonga | 3,682,138 (2,372,330, 5,602,475) | NA | 1,289,107 (979,722, 1,934,550) | 628,505 (477,664, 1,053,807) | 1,764,526 (914,944, 2,614,107) |
| Trinidad and Tobago | 292,143,080 (172,978,489, 428,643,097) | 8,121,397 (5,684,978, 12,701,728) | 116,511,749 (81,558,224, 163,398,580) | 61,490,009 (43,043,006, 83,195,219) | 106,019,925 (42,692,281, 169,347,570) |
| Tunisia | 385,702,713 (289,568,183, 463,929,763) | 32,570,945 (22,799,662, 40,332,906) | 122,641,428 (85,848,999, 147,267,113) | 55,222,621 (38,655,835, 68,057,994) | 175,267,718 (142,263,687, 208,271,750) |
| Turkey | 25,692,855,211 (17,970,223,384, 34,612,836,463) | 689,029,284 (502,991,377, 937,164,581) | 12,984,387,117 (9,478,602,595, 17,436,801,863) | 4,126,639,682 (3,012,446,968, 5,429,454,206) | 7,892,799,128 (4,976,182,444, 10,809,415,813) |
| Uganda | 504,876,929 (339,646,859, 688,771,949) | 43,369,388 (32,960,735, 62,038,648) | 70,735,341 (53,758,859, 94,934,838) | 84,753,819 (64,412,902, 108,276,064) | 306,018,381 (188,514,362, 423,522,399) |
| Ukraine | 5,902,003,455 (3,603,395,194, 10,373,176,484) | 563,436,697 (428,211,890, 1,039,459,791) | 2,914,901,782 (2,215,325,354, 5,243,381,777) | 563,957,921 (411,689,282, 919,089,474) | 1,859,707,055 (548,168,668, 3,171,245,442) |
| United Arab Emirates | 3,177,105,111 (2,017,823,610, 5,026,649,881) | 115,886,734 (84,597,316, 176,621,983) | 1,442,856,916 (1,053,285,548, 2,289,136,433) | 622,869,574 (454,694,789, 995,153,647) | 995,491,887 (425,245,957, 1,565,737,818) |
| United Kingdom | 30,495,994,397 (20,304,148,466, 44,304,626,159) | 1,556,661,821 (1,136,363,130, 3,807,234,325) | 11,690,336,499 (8,533,945,645, 15,573,041,392) | 6,511,239,127 (4,753,204,563, 9,329,471,671) | 10,737,756,950 (5,880,635,128, 15,594,878,771) |
| United Republic of Tanzania | 851,446,440 (655,379,996, 1,048,022,063) | 66,464,385 (50,512,933, 83,163,125) | 241,440,876 (183,495,066, 301,605,687) | 102,374,122 (77,804,333, 124,486,803) | 441,167,057 (343,567,665, 538,766,448) |
| United States of America | 238,199,291,248 (164,982,156,936, 304,245,575,434) | 5,929,653,308 (4,150,757,316, 8,061,801,848) | 99,805,996,739 (69,864,197,717, 122,129,000,000) | 47,299,911,266 (33,109,937,886, 61,584,773,586) | 85,163,729,935 (57,857,264,017, 112,470,000,000) |
| Uruguay | 1,305,061,017 (892,136,425, 1,859,602,097) | 48,966,154 (34,276,308, 107,801,577) | 532,691,105 (372,883,773, 796,571,015) | 223,860,382 (156,702,267, 284,416,828) | 499,543,377 (328,274,077, 670,812,676) |
| Uzbekistan | 627,875,233 (445,367,427, 1,109,257,620) | 43,908,682 (33,370,598, 83,210,246) | 404,433,153 (307,369,196, 764,127,758) | 29,008,743 (22,046,645, 43,451,294) | 150,524,655 (82,580,987, 218,468,322) |
| Vanuatu | 1,944,006 (1,337,455, 2,843,705) | 32,670 (23,849, 111,096) | 1,172,259 (890,917, 1,614,956) | 91,881 (69,830, 176,122) | 647,196 (352,860, 941,531) |
| VietNam | 3,518,394,635 (2,706,439,580, 4,220,763,340) | 268,483,223 (204,047,250, 322,679,261) | 2,719,044,808 (2,066,474,054, 3,218,816,432) | 233,393,770 (177,379,265, 285,359,758) | 297,472,834 (258,539,011, 393,907,889) |
| Zambia | 735,678,923 (116,995,237, 1040096750) | 12,133,013 (8,493,109, 16,231,810) | 40,471,415 (28,329,990, 52,225,693) | 28,253,092 (19,777,164, 41,535,368) | 654,821,403 (60,394,974, 930,103,879) |
| Zimbabwe | 214,543,137 (167,673,970, 478,190,400) | 11,138,149 (8,464,993, 22,742,621) | 57,174,567 (43,452,671, 118,505,160) | 30,700,781 (23,332,593, 55,347,625) | 115,529,641 (92,423,713, 281,594,994) |

Note: GDP, Gross Domestic Product

# Table S3 Projected Economic Burden of Hematologic Malignancies in Various Countries, 2040 (Discounted to 2021 US$)

| Country | Hematologic Malignancies Economic Burden ($) | Hodgkin lymphoma Economic Burden ($) | Leukemia Economic Burden ($) | Multiple myeloma Economic Burden ($) | Non-Hodgkin lymphoma Economic Burden ($) |
| --- | --- | --- | --- | --- | --- |
| Afghanistan | 726,054,584 (551,801,484, 1,332,110,739) | 48,867,489 (37,139,292, 88,452,698) | 468,889,810 (356,356,255, 883,116,054) | 33,172,829 (25,211,350, 62,423,068) | 175,124,456 (133,094,587, 298,118,919) |
| Albania | 96,939,766 (72,888,732, 241,798,534) | 6,416,978 (4,684,394, 14,590,828) | 55,841,228 (42,439,334, 149,537,010) | 14,915,530 (11,335,803, 27,948,192) | 19,766,030 (14,429,202, 49,722,503) |
| Algeria | 749,943,631 (524,960,542, 1,141,056,958) | 65,605,680 (45,923,976, 101,672,446) | 255,470,259 (178,829,181, 387,288,513) | 117,615,606 (82,330,924, 183,152,723) | 311,252,087 (217,876,461, 468,943,276) |
| Angola | 433,848,654 (320,970,727, 801,043,654) | 20,229,002 (14,767,172, 34,848,168) | 142,040,329 (107,950,650, 266,008,893) | 39,033,342 (28,494,340, 71,241,127) | 232,545,981 (169,758,566, 428,945,467) |
| Antigua and Barbuda | 10,683,684 (7,799,089, 22,685,633) | 29 (21, 2,246) | 4,299,485 (3,138,624, 8,846,593) | 2,912,735 (2,126,297, 6,400,995) | 3,471,434 (2,534,147, 7,435,798) |
| Argentina | 2,967,838,410 (2,219,643,475, 6,884,894,896) | 101,917,791 (74,399,988, 586,611,351) | 1,281,327,522 (973,808,917, 3,037,195,911) | 489,387,017 (371,934,133, 983,584,714) | 1,095,206,080 (799,500,438, 2,277,502,920) |
| Armenia | 372,447,047 (283,059,756, 1,610,599,802) | 14,027,460 (10,660,869, 65,056,546) | 182,620,886 (138,791,873, 835,617,849) | 88,626,417 (67,356,077, 296,885,053) | 87,172,285 (66,250,937, 413,040,355) |
| Australia | 13,376,024,708 (3,745,614,485, 32,596,275,182) | 290,308,346 (220,634,343, 680,910,052) | 5,020,449,678 (636,188,007, 12,840,983,708) | 3,264,643,664 (2,252,604,128, 10,109,323,388) | 4,800,623,020 (636,188,007, 8,965,058,034) |
| Austria | 2,795,829,478 (2,675,132,698, 6,068,927,147) | 96,110,749 (812,326,775, 309,908,654) | 1,186,210,690 (818,485,376, 2,599,946,303) | 596,427,297 (411,534,835, 1,182,787,151) | 917,080,741 (632,785,712, 1,976,285,039) |
| Azerbaijan | 321,522,402 (244,357,025, 937,381,892) | 28,097,919 (21,354,418, 81,568,851) | 221,151,122 (168,074,853, 601,997,570) | 13,968,850 (10,616,326, 40,704,035) | 58,304,511 (44,311,428, 213,111,435) |
| Bahamas | 51,395,861 (35,977,103, 93,682,653) | 1,355,888 (949,122, 3,111,165) | 14,223,267 (9,956,287, 26,170,691) | 17,045,650 (11,931,955, 30,207,139) | 18,771,055 (13,139,739, 34,193,658) |
| Bahrain | 540,717,354 (378,502,148, 1,098,732,775) | 22,346,720 (15,642,704, 50,484,053) | 249,939,371 (174,957,560, 486,151,230) | 100,528,935 (70,370,255, 187,458,397) | 167,902,328 (117,531,629, 374,639,096) |
| Bangladesh | 12,451,185,892 (9,089,365,701, 25,425,898,410) | 828,935,181 (605,122,682, 1,539,929,003) | 5,544,422,048 (4,047,428,095, 11,086,892,750) | 2,011,519,448 (1,468,409,197, 3,673,867,313) | 4,066,309,215 (2,968,405,727, 9,125,209,344) |
| Barbados | 20,952,279 (15,295,164, 42,303,946) | 444,647 (324,592, 1,091,333) | 7,791,654 (5,687,907, 16,128,773) | 3,237,675 (2,363,503, 6,136,014) | 9,478,303 (6,919,161, 18,947,828) |
| Belarus | 3,466,424,336 (2,391,832,792, 11,054,485,730) | 232,315,227 (160,297,506, 756,799,272) | 1,519,769,390 (1,048,640,879, 4,167,020,263) | 487,776,326 (336,565,665, 1,446,230,696) | 1,226,563,394 (846,328,742, 4,684,435,499) |
| Belgium | 6,546,038,945 (4,873,262,176, 17,160,158,412) | 133,267,394 (101,283,219, 281,128,512) | 3,390,914,101 (2,475,367,294, 10,469,691,736) | 1,101,917,618 (837,457,390, 1,988,085,018) | 1,919,939,832 (1,459,154,273, 4,421,253,146) |
| Belize | 6,028,379 (4,400,716, 12,340,846) | 336,938 (245,965, 816,163) | 2,941,253 (2,147,115, 5,950,600) | 733,573 (535,508, 1,657,429) | 2,016,615 (1,472,129, 3,916,654) |
| Benin | 106,726,361 (74,708,452, 191,574,371) | 2,283,877 (1,598,714, 5,297,699) | 39,238,587 (27,467,011, 72,164,751) | 2,663,814 (1,864,670, 5,503,606) | 62,540,082 (43,778,057, 108,608,315) |
| Bermuda | 46,899,429 (35,643,566, 105,563,152) | 546 (415, 27,044) | 16,274,159 (12,368,361, 36,980,557) | 14,724,840 (11,190,879, 32,553,835) | 15,899,884 (12,083,912, 36,001,717) |
| Bhutan | 103,921,996 (72,745,397, 197,093,519) | 5,814,856 (4,070,399, 11,355,713) | 43,588,594 (30,512,016, 80,591,217) | 20,343,091 (14,240,164, 40,124,099) | 34,175,456 (23,922,819, 65,022,490) |
| Bolivia (Plurinational State of) | 1,799,641,351 (1,367,727,427, 2,766,614,196) | 54,570,741 (41,473,763, 91,696,729) | 831,009,877 (631,567,506, 1,217,012,393) | 211,654,422 (160,857,360, 344,786,357) | 702,406,311 (533,828,796, 1,113,118,717) |
| Bosnia and Herzegovina | 314,579,738 (239,080,601, 768,178,995) | 17,720,266 (13,467,402, 47,879,786) | 127,713,576 (97,062,318, 309,328,168) | 81,762,515 (62,139,512, 155,798,633) | 87,383,382 (66,411,370, 255,172,409) |
| Botswana | 187,320,881 (131,124,616, 525,387,854) | 5,876,782 (4,113,747, 14,672,859) | 66,421,334 (46,494,934, 166,671,993) | 33,932,243 (23,752,570, 74,259,401) | 81,090,522 (56,763,365, 269,783,602) |
| Brazil | 9,607,310,669 (6,725,117,467, 18,796,975,881) | 308,941,543 (216,259,080, 828,963,152) | 3,959,767,639 (2,771,837,347, 6,745,957,798) | 2,055,696,892 (1,438,987,824, 5,319,205,494) | 3,282,904,595 (2,298,033,216, 5,902,849,437) |
| Brunei Darussalam | 217,514,714 (165,311,183, 440,512,296) | 6,650,409 (5,054,311, 15,554,406) | 85,884,061 (65,271,886, 165,131,951) | 31,527,844 (23,961,162, 63,375,496) | 93,452,400 (71,023,824, 196,450,442) |
| Bulgaria | 2,432,821,315 (1,678,646,707, 9,746,757,331) | 124,712,783 (86,051,820, 374,102,499) | 1,284,494,161 (886,300,971, 7,016,701,240) | 380,002,926 (262,202,019, 803,871,396) | 643,611,446 (444,091,897, 1,552,082,195) |
| Burkina Faso | 134,375,861 (94,063,102, 285,158,583) | 4,023,242 (2,816,269, 9,992,665) | 45,952,355 (32,166,649, 103,209,393) | 4,252,372 (2,976,660, 8,342,737) | 80,147,892 (56,103,524, 163,613,787) |
| Burundi | 21,690,818 (15,160,490, 39,250,684) | 2,308,259 (1,592,699, 3,837,961) | 5,657,049 (3,959,934, 9,721,676) | 1,889,039 (1,322,327, 3,706,735) | 11,836,472 (8,285,531, 21,984,312) |
| Cabo Verde | 5,221,411 (3,654,988, 15,023,426) | NA | 2,295,601 (1,606,921, 4,331,038) | 558,864 (391,205, 1,676,500) | 2,366,946 (1,656,862, 9,015,882) |
| Cambodia | 810,386,239 (567,270,368, 1,270,912,703) | 16,249,774 (11,374,842, 27,754,790) | 468,356,635 (327,849,645, 700,446,299) | 40,998,073 (28,698,651, 72,408,977) | 284,781,757 (199,347,230, 470,302,636) |
| Cameroon | 357,574,390 (271,756,536, 606,926,727) | 6,624,028 (5,034,262, 14,001,385) | 95,635,936 (72,683,311, 171,915,412) | 12,335,598 (9,375,054, 23,401,127) | 242,978,827 (184,663,909, 397,608,804) |
| Canada | 11,613,592,681 (8,333,828,221, 20,918,198,753) | 253,204,041 (177,242,828, 511,025,345) | 4,483,229,173 (3,272,757,296, 8,124,508,830) | 2,327,215,655 (1,698,867,428, 4,037,994,731) | 4,549,943,812 (3,184,960,669, 8,244,669,847) |
| Central African Republic | 4,689,452 (3,282,616, 7,486,057) | 388,217 (271,752, 661,419) | 1,773,005 (1,241,103, 2,816,529) | 194,186 (135,930, 360,771) | 2,334,043 (1,633,830, 3,647,339) |
| Chad | 20,385,117 (14,881,135, 35,618,102) | 685,144 (500,155, 1,432,555) | 7,902,052 (5,768,498, 14,046,261) | 472,626 (345,017, 983,177) | 11,325,295 (8,267,465, 19,156,109) |
| Chile | 2,887,767,047 (2,108,069,945, 8,324,842,033) | 170,296,531 (124,316,468, 2,168,199,801) | 1,072,310,983 (782,787,018, 2,254,455,190) | 622,949,101 (454,752,844, 1,769,967,740) | 1,022,210,431 (746,213,615, 2,132,219,302) |
| China | 275,034,711,404 (204,796,260,668, 621,941,279,969) | 4,502,845,322 (3,422,162,445, 9,695,279,969) | 141,012,000,000 (102,939,000,000, 235,005,000,000) | 33,612,182,642 (25,545,258,808, 142,298,000,000) | 95,907,683,440 (72,889,839,415, 234,943,000,000) |
| Colombia | 5,702,136,547 (3,991,495,582, 14,769,851,358) | 196,460,490 (137,522,343, 1,201,853,974) | 2,119,368,352 (1,483,557,846, 5,105,636,737) | 1,425,417,266 (997,792,087, 3,673,832,572) | 1,960,890,439 (1,372,623,307, 4,788,528,075) |
| Comoros | 11,214,037 (7,849,826, 32,373,526) | 1,159,681 (811,776, 3,019,708) | 3,144,894 (2,201,426, 9,939,938) | 1,182,839 (827,987, 2,334,166) | 5,726,623 (4,008,636, 17,079,714) |
| Congo | 45,444,788 (34,481,844, 93,289,883) | 1,873,144 (1,367,395, 3,446,228) | 13,209,566 (10,039,270, 26,310,065) | 4,268,564 (3,244,109, 7,969,376) | 26,093,513 (19,831,070, 55,564,213) |
| Costa Rica | 2,482,020,498 (1,737,414,349, 9,114,543,986) | 76,527,445 (53,569,212, 168,932,054) | 1,138,587,898 (797,011,529, 5,090,405,950) | 417,771,380 (292,439,966, 1,273,840,055) | 849,133,775 (594,393,642, 2,581,365,927) |
| Coted' Ivoire | 2,659,337,616 (2,021,096,588, 5,892,255,642) | 140,158,807 (106,520,693, 344,555,482) | 309,032,639 (234,864,806, 772,206,710) | 559,142,532 (424,948,325, 981,595,781) | 1,651,003,637 (1,254,762,764, 3,793,897,668) |
| Croatia | 1,523,373,974 (1,112,063,001, 5,464,422,782) | 32,119,508 (23,447,241, 221,744,818) | 612,613,258 (447,207,679, 1,648,235,253) | 323,798,891 (236,373,190, 953,247,534) | 554,842,316 (405,034,891, 2,641,195,177) |
| Cyprus | 235,007,308 (171,555,335, 415,456,295) | 8,133,594 (5,937,524, 16,681,969) | 99,131,233 (72,365,800, 172,980,315) | 46,893,834 (34,232,499, 84,711,007) | 80,848,647 (59,019,512, 141,083,004) |
| Czechia | 5,207,515,855 (3,593,185,940, 10,666,870,037) | 146,687,937 (101,214,677, 257,787,907) | 2,104,147,919 (1,451,862,064, 4,451,102,066) | 1,117,002,752 (770,731,899, 2,515,858,638) | 1,839,677,247 (1,269,377,300, 3,442,121,426) |
| Democratic Republic of the Congo | 545,351,733 (398,106,765, 906,626,389) | 32,910,580 (24,024,724, 53,920,797) | 173,962,757 (126,992,812, 282,626,716) | 36,868,087 (26,913,704, 70,122,781) | 301,610,309 (220,175,525, 499,956,096) |
| Denmark | 3,559,596,686 (2,705,293,482, 11,134,630,013) | 37,289,308 (28,339,874, 97,302,261) | 1,716,916,565 (1,304,856,590, 4,374,604,395) | 799,566,272 (607,670,367, 2,982,674,461) | 1,005,824,541 (764,426,651, 3,680,048,896) |
| Dominica | 10,688,222 (7,481,756, 21,706,911) | 240,988 (168,691, 604,071) | 4,526,804 (3,168,763, 9,039,606) | 1,417,720 (992,404, 3,121,903) | 4,502,711 (3,151,898, 8,941,330) |
| Dominican Republic | 1,691,346,290 (1,220,136,698, 3,312,575,156) | 9,379,878 (6,565,914, 20,193,162) | 786,199,619 (573,925,722, 1,429,840,906) | 420,276,870 (306,802,115, 750,421,994) | 475,489,924 (332,842,947, 1,112,119,094) |
| Ecuador | 731,933,055 (507,198,629, 3,439,318,718) | 30,926,015 (23,503,771, 310,716,038) | 310,258,896 (214,078,638, 1,295,847,298) | 105,342,304 (72,686,189, 750,718,310) | 285,405,840 (196,930,030, 1,082,037,072) |
| Egypt | 15,655,041,894 (11,897,831,839, 37,594,706,764) | 134,398,764 (102,143,061, 281,995,722) | 11,487,819,138 (8,730,742,545, 28,932,994,089) | 792,593,355 (602,370,950, 1,315,106,453) | 3,240,230,637 (2,462,575,284, 7,064,610,500) |
| El Salvador | 300,270,177 (219,197,229, 884,107,354) | 11,144,886 (8,135,767, 22,159,430) | 177,040,921 (129,239,872, 468,903,241) | 24,255,128 (17,706,243, 42,952,017) | 87,829,243 (64,115,347, 350,092,666) |
| Equatorial Guinea | 24,356,819 (18,511,183, 51,894,878) | 495,867 (376,859, 1,107,696) | 7,034,010 (5,345,847, 13,900,939) | 2,981,147 (2,265,672, 6,095,208) | 13,845,795 (10,522,805, 30,791,035) |
| Estonia | 1,087,048,868 (793,545,674, 3,736,405,692) | 15,140,722 (11,052,727, 40,381,299) | 336,602,263 (245,719,652, 875,838,295) | 292,784,805 (213,732,908, 923,229,501) | 442,521,078 (323,040,387, 1,896,956,598) |
| Eswatini | 44,359,482 (33,049,652, 109,062,943) | 1,682,620 (1,278,792, 4,002,522) | 14,507,614 (10,590,559, 32,308,200) | 7,610,862 (5,555,929, 16,469,633) | 20,558,385 (15,624,373, 56,282,589) |
| Ethiopia | 10,615,894,711 (7,900,775,116, 17,236,098,231) | 790,274,583 (600,608,683, 1,319,655,346) | 5,576,828,799 (4,071,085,023, 8,781,726,315) | 619,764,838 (471,021,277, 1,098,470,986) | 3,629,026,490 (2,758,060,133, 6,036,245,584) |
| Fiji | 64,052,034 (48,679,546, 122,284,105) | 1,654,813 (1,257,658, 4,928,646) | 43,895,292 (33,360,422, 78,164,311) | 4,999,536 (3,799,647, 10,137,259) | 13,502,392 (10,261,818, 29,053,891) |
| Finland | 2,446,152,817 (1,831,990,775, 5,146,411,693) | 39,771,140 (30,226,066, 103,095,199) | 902,845,545 (659,077,248, 2,187,964,943) | 463,127,212 (351,976,681, 903,322,034) | 1,040,408,920 (790,710,779, 1,952,029,517) |
| France | 24,017,794,164 (16,812,455,916, 49,104,939,013) | 618,741,522 (433,119,066, 1,363,881,968) | 11,214,375,856 (7,850,063,099, 20,360,713,156) | 4,347,025,081 (3,042,917,557, 10,237,941,811) | 7,837,651,705 (5,486,356,194, 17,142,402,078) |
| Gabon | 102,214,624 (77,587,692, 177,286,544) | 3,180,762 (2,321,956, 6,148,215) | 26,519,608 (20,154,902, 45,557,965) | 16,449,217 (12,501,405, 30,401,210) | 56,065,038 (42,609,429, 95,179,154) |
| Gambia | 6,964,381 (4,875,066, 20,228,857) | 730,105 (511,073, 1,644,825) | 1,123,354 (786,348, 2,658,936) | 165,766 (116,036, 382,462) | 4,945,156 (3,461,609, 15,542,633) |
| Georgia | 865,575,825 (597,247,319, 6,032,965,284) | 102,126,411 (70,467,223, 744,869,800) | 368,266,477 (254,103,869, 1,511,460,317) | 153,587,859 (105,975,623, 1,855,410,615) | 241,595,079 (166,700,604, 1,921,224,552) |
| Germany | 43,517,771,647 (33,073,506,451, 151,504,940,021) | 1,086,491,123 (825,733,253, 4,228,818,923) | 21,417,199,512 (16,277,071,629, 40,905,563,739) | 7,012,695,093 (5,329,648,271, 15,643,002,611) | 14,001,385,919 (10,641,053,298, 90,727,554,748) |
| Ghana | 746,827,473 (522,779,231, 2,553,063,193) | 2,622,229 (1,835,560, 9,634,678) | 194,176,304 (135,923,413, 633,343,971) | 28,294,152 (19,805,907, 53,521,473) | 521,734,788 (365,214,351, 1,856,563,071) |
| Greece | 1,993,373,106 (1,414,203,083, 5,010,873,002) | 173,809,488 (126,880,926, 734,889,033) | 974,974,587 (682,482,211, 1,967,629,582) | 390,334,892 (273,234,425, 732,225,339) | 454,254,139 (331,605,521, 1,576,129,047) |
| Grenada | 13,875,845 (9,713,091, 27,890,745) | 131,627 (92,139, 352,846) | 3,731,952 (2,612,367, 7,622,087) | 2,563,744 (1,794,620, 5,518,335) | 7,448,522 (5,213,965, 14,397,478) |
| Guatemala | 1,014,529,559 (710,170,692, 3,872,100,292) | 30,240,340 (21,168,238, 61,667,440) | 642,187,526 (449,531,268, 2,601,257,916) | 112,514,740 (78,760,318, 198,704,290) | 229,586,954 (160,710,868, 1,010,470,646) |
| Guinea | 144,256,496 (100,979,547, 246,593,694) | 21,518,898 (15,063,228, 35,547,990) | 18,200,409 (12,740,286, 34,252,043) | 10,151,369 (7,105,959, 18,844,050) | 94,385,820 (66,070,074, 157,949,612) |
| Guinea-Bissau | 14,472,201 (10,564,706, 26,017,049) | 358,077 (261,396, 725,166) | 4,055,382 (2,960,429, 7,616,198) | 571,078 (416,887, 1,501,898) | 9,487,663 (6,925,994, 16,173,787) |
| Guyana | 198,654,393 (145,017,707, 667,114,317) | 11,619,916 (8,482,538, 24,729,297) | 81,013,242 (59,139,667, 303,719,300) | 29,490,157 (21,527,815, 153,220,412) | 76,531,078 (55,867,687, 185,445,308) |
| Haiti | 116,473,358 (81,531,350, 202,149,824) | 6,706,079 (4,694,255, 11,469,870) | 58,139,157 (40,697,410, 102,843,183) | 9,070,692 (6,349,484, 14,896,588) | 42,557,430 (29,790,201, 72,940,183) |
| Honduras | 214,281,523 (149,997,066, 348,265,532) | 5,702,102 (3,991,471, 10,246,332) | 137,380,745 (96,166,521, 218,974,961) | 28,153,776 (19,707,643, 47,769,059) | 43,044,901 (30,131,430, 71,275,180) |
| Hungary | 3,400,849,140 (2,584,645,347, 7,301,794,409) | 83,107,323 (63,161,565, 224,452,408) | 1,677,124,935 (1,274,614,951, 2,832,750,121) | 652,989,367 (496,271,919, 1,592,778,282) | 987,627,515 (750,596,912, 2,651,813,598) |
| Iceland | 290,480,002 (220,764,802, 540,804,719) | 5,782,678 (4,394,836, 14,287,493) | 111,190,472 (84,504,759, 200,807,327) | 53,758,500 (40,856,460, 101,487,958) | 119,748,351 (91,008,747, 224,221,940) |
| India | 55,962,994,127 (40,852,985,713, 97,386,683,236) | 2,798,887,149 (2,043,187,619, 4,766,522,043) | 23,159,833,736 (16,906,678,627, 38,279,665,642) | 10,650,672,612 (7,774,991,007, 17,902,519,263) | 19,353,600,630 (14,128,128,460, 36,437,976,288) |
| Indonesia | 15,618,884,048 (11,411,452,517, 22,573,397,499) | 322,238,720 (244,901,427, 467,523,268) | 9,676,885,740 (7,064,126,590, 13,860,031,130) | 927,707,791 (677,226,687, 1,375,018,808) | 4,692,051,797 (3,425,197,812, 6,870,824,293) |
| Iran (Islamic Republic of) | 641,449,796 (449,014,857, 1,152,674,719) | 20,034,717 (14,024,302, 66,591,602) | 414,028,052 (289,819,636, 681,981,774) | 59,986,856 (41,990,799, 120,813,559) | 147,400,171 (103,180,120, 283,287,785) |
| Iraq | 1,034,576,321 (724,203,425, 1,759,277,616) | 26,287,213 (18,401,049, 60,682,587) | 539,868,988 (377,908,292, 869,830,675) | 170,150,220 (119,105,154, 284,996,788) | 298,269,899 (208,788,930, 543,767,566) |
| Ireland | 3,045,040,867 (2,222,879,833, 7,454,930,810) | 122,004,256 (89,063,107, 274,608,335) | 985,500,724 (719,415,529, 2,330,922,164) | 921,488,324 (672,686,476, 1,952,511,066) | 1,016,047,563 (741,714,721, 2,896,889,245) |
| Israel | 2,566,532,117 (1,839,350,602, 6,818,957,235) | 63,051,448 (46,027,557, 113,273,443) | 855,446,087 (590,257,800, 2,288,779,558) | 617,069,089 (450,460,435, 1,594,747,422) | 1,030,965,493 (752,604,810, 2,822,156,812) |
| Italy | 17,945,696,003 (13,638,728,962, 40,878,303,323) | 920,188,126 (699,342,976, 4,089,990,463) | 6,924,622,324 (5,262,712,966, 12,674,536,242) | 3,890,780,638 (2,956,993,285, 9,257,188,706) | 6,210,104,915 (4,719,679,735, 14,856,587,912) |
| Jamaica | 96,267,758 (70,275,464, 1,079,203,106) | 1,588,857 (1,159,866, 4,916,816) | 28,133,357 (20,537,351, 191,570,415) | 21,106,992 (15,408,104, 272,510,260) | 45,438,552 (33,170,143, 610,205,616) |
| Japan | 22,717,532,455 (15,902,272,718, 40,231,115,051) | 339,352,937 (237,547,056, 2,728,442,701) | 9,186,359,701 (6,430,451,791, 14,279,464,826) | 3,194,086,933 (2,235,860,853, 5,255,440,215) | 9,997,732,884 (6,998,413,018, 17,967,767,309) |
| Jordan | 195,529,176 (142,736,298, 411,483,485) | 2,799,968 (2,043,976, 6,481,192) | 103,021,237 (75,205,503, 215,606,783) | 15,309,203 (11,175,718, 28,719,404) | 74,398,768 (54,311,101, 160,676,105) |
| Kazakhstan | 3,115,661,994 (2,367,903,116, 14,494,278,291) | 193,993,651 (147,435,175, 735,185,754) | 1,537,677,834 (1,168,635,154, 4,758,547,184) | 494,207,073 (375,597,375, 2,166,939,433) | 889,783,436 (676,235,412, 6,833,605,920) |
| Kenya | 2,630,903,136 (1,841,632,195, 4,852,586,840) | 134,229,147 (93,960,403, 261,178,191) | 716,969,852 (501,878,897, 1,335,024,380) | 562,378,657 (393,665,060, 937,737,803) | 1,217,325,480 (852,127,836, 2,318,646,466) |
| Kiribati | 3,137,928 (2,290,687, 6,536,886) | NA | 2,468,255 (1,801,826, 4,796,615) | 107,227 (78,276, 330,655) | 562,446 (410,586, 1,409,595) |
| Kuwait | 315,429,945 (230,263,860, 3,476,598,754) | 9,292,593 (6,783,593, 239,754,390) | 131,692,007 (96,135,165, 551,823,440) | 25,806,407 (18,838,677, 178,261,913) | 148,638,938 (108,506,425, 2,506,759,012) |
| Kyrgyzstan | 166,754,855 (115,060,850, 697,011,996) | 10,588,034 (7,305,743, 114,285,978) | 102,391,873 (70,650,392, 312,646,797) | 17,526,942 (12,093,590, 62,570,843) | 36,248,007 (25,011,125, 207,508,378) |
| Lao People's Democratic Republic | 893,389,833 (635,800,753, 1,381,081,064) | 18,576,772 (13,561,044, 32,857,037) | 545,794,192 (382,055,935, 821,379,053) | 46,778,369 (34,148,210, 83,985,553) | 282,240,500 (206,035,565, 442,859,421) |
| Latvia | 985,926,074 (749,303,816, 3,627,001,081) | 29,897,802 (22,722,329, 75,784,457) | 353,801,120 (268,888,851, 1,499,612,060) | 265,777,073 (201,990,575, 828,864,721) | 336,450,080 (255,702,061, 1,222,739,843) |
| Lebanon | 389,908,677 (292,588,099, 836,416,328) | 14,727,615 (11,192,988, 26,652,056) | 124,749,839 (91,067,383, 202,339,460) | 56,879,062 (43,228,087, 111,432,960) | 193,552,160 (147,099,641, 495,991,852) |
| Lesotho | 12,792,530 (8,954,771, 34,772,739) | 627,794 (439,456, 1,655,871) | 4,675,119 (3,272,583, 11,948,651) | 1,669,942 (1,168,959, 3,529,546) | 5,819,676 (4,073,773, 17,638,671) |
| Liberia | 29,420,324 (20,594,227, 74,110,297) | 544,876 (381,413, 1,191,272) | 8,748,409 (6,123,887, 24,129,519) | 726,443 (508,510, 1,675,199) | 19,400,597 (13,580,418, 47,114,308) |
| Libya | 85,342,569 (59,739,798, 194,066,028) | 7,955,659 (5,568,962, 15,537,834) | 36,653,011 (25,657,107, 83,584,806) | 8,750,859 (6,125,601, 16,352,950) | 31,983,040 (22,388,128, 78,590,438) |
| Lithuania | 2,295,174,648 (1,675,477,493, 8,159,361,962) | 71,984,303 (52,548,541, 286,626,140) | 860,470,528 (628,143,485, 2,441,349,425) | 559,416,622 (408,374,134, 1,476,288,727) | 803,303,196 (586,411,333, 3,955,097,670) |
| Luxembourg | 406,873,451 (309,223,822, 743,470,641) | 6,634,335 (5,042,095, 15,656,378) | 192,560,540 (146,346,010, 327,751,072) | 89,344,725 (67,901,991, 167,682,537) | 118,333,850 (89,933,726, 232,380,653) |
| Madagascar | 57,304,727 (41,832,451, 89,423,447) | 5,426,955 (3,961,677, 8,433,255) | 15,704,333 (11,464,163, 24,437,933) | 5,851,300 (4,271,449, 9,726,404) | 30,322,138 (22,135,161, 46,825,856) |
| Malawi | 72,134,709 (50,494,296, 119,113,957) | 2,168,055 (1,517,638, 3,458,871) | 3,999,161 (2,799,413, 6,509,122) | 3,000,081 (2,100,057, 5,144,323) | 62,967,411 (44,077,188, 104,001,641) |
| Malaysia | 16,973,789,471 (12,390,866,313, 76,944,117,039) | 256,597,763 (187,316,367, 487,221,395) | 8,015,093,461 (5,851,018,226, 32,232,327,644) | 765,799,777 (559,033,837, 1,342,756,031) | 7,936,298,470 (5,793,497,883, 42,881,811,969) |
| Maldives | 9,301,127 (6,789,822, 18,061,033) | 151,303 (110,451, 438,299) | 4,993,994 (3,645,616, 9,008,415) | 1,629,718 (1,189,694, 3,562,017) | 2,526,112 (1,844,062, 5,052,302) |
| Mali | 55,077,286 (38,554,100, 98,157,773) | 11,449,183 (8,014,428, 20,270,064) | 15,347,182 (10,743,028, 28,364,625) | 8,060 (5,642, 26,217) | 28,272,860 (19,791,002, 49,496,867) |
| Malta | 206,489,997 (155,819,541, 402,969,100) | 5,945,184 (4,518,340, 13,682,615) | 90,751,339 (68,971,018, 163,427,348) | 37,095,210 (27,079,503, 69,939,359) | 72,698,264 (55,250,680, 155,919,779) |
| Marshall Islands | 3,230,282 (2,455,014, 7,256,182) | NA | 1,836,889 (1,396,036, 3,876,032) | 215,807 (164,013, 655,893) | 1,177,586 (894,965, 2,724,232) |
| Mauritania | 161,637,043 (117,995,041, 286,481,526) | 2,183,361 (1,593,854, 4,489,654) | 48,863,631 (35,670,451, 85,290,224) | 5,204,279 (3,799,124, 11,474,341) | 105,385,771 (76,931,613, 185,227,308) |
| Mauritius | 274,527,459 (192,169,221, 1,658,336,912) | 23,322,276 (16,325,593, 122,577,370) | 84,804,091 (59,362,864, 250,261,628) | 77,859,608 (54,501,726, 769,337,182) | 88,541,485 (61,979,039, 516,160,732) |
| Mexico | 13,565,775,817 (9,496,043,072, 28,772,559,400) | 714,201,651 (499,941,156, 2,951,201,075) | 6,329,395,766 (4,430,577,036, 9,767,414,083) | 2,067,131,014 (1,446,991,710, 5,894,366,969) | 4,455,047,386 (3,118,533,170, 10,159,577,273) |
| Micronesia (Federated States of) | 1,204,390 (879,205, 2,438,110) | 4 (3, 246) | 664,816 (485,315, 1,265,857) | 81,431 (59,445, 229,921) | 458,139 (334,442, 942,087) |
| Mongolia | 247,083,780 (187,783,672, 675,061,613) | 15,447,739 (11,740,282, 32,884,879) | 136,672,074 (103,870,777, 366,565,435) | 24,149,986 (18,353,989, 48,171,294) | 70,813,980 (53,818,625, 227,440,005) |
| Montenegro | 117,012,413 (88,929,434, 224,142,583) | 8,557,508 (6,503,706, 19,787,189) | 51,436,615 (39,091,827, 96,514,641) | 24,888,051 (18,914,919, 48,338,406) | 32,130,240 (24,418,982, 59,502,347) |
| Morocco | 453,495,599 (344,656,655, 693,464,206) | 35,203,052 (26,754,320, 58,029,380) | 77,996,964 (59,277,693, 116,430,224) | 77,645,128 (59,010,298, 124,692,061) | 262,650,454 (199,614,345, 394,312,542) |
| Mozambique | 128,394,769 (97,580,025, 234,986,665) | 14,309,597 (10,875,294, 28,452,903) | 48,994,925 (37,236,143, 90,358,675) | 16,146,732 (12,271,517, 27,379,421) | 48,943,515 (37,197,071, 88,795,667) |
| Myanmar | 1,787,198,830 (1,283,557,957, 2,897,542,932) | 37,619,647 (26,333,753, 66,263,822) | 1,083,959,208 (791,290,222, 1,758,573,647) | 90,155,925 (63,109,147, 145,104,876) | 575,464,051 (402,824,836, 927,600,588) |
| Namibia | 134,021,157 (93,814,810, 244,862,403) | 7,449,934 (5,214,954, 13,834,989) | 36,049,663 (25,234,764, 62,244,290) | 19,604,050 (13,722,835, 37,034,067) | 70,917,510 (49,642,257, 131,749,057) |
| Nepal | 935,273,255 (656,382,393, 1,659,641,718) | 56,370,476 (41,150,448, 100,429,445) | 400,461,415 (280,322,991, 677,091,376) | 132,144,120 (92,500,884, 245,945,103) | 346,297,244 (242,408,071, 636,175,793) |
| Netherlands | 6,305,913,244 (4,466,579,133, 13,307,018,447) | 190,952,680 (133,666,876, 366,126,537) | 2,386,841,468 (1,670,789,027, 5,462,790,705) | 1,747,995,410 (1,276,036,650, 3,384,497,455) | 1,980,123,686 (1,386,086,580, 4,093,603,750) |
| New Zealand | 2,630,283,807 (1,920,107,180, 13,001,110,584) | 39,426,665 (28,781,465, 394,706,993) | 854,752,396 (623,969,249, 4,466,861,159) | 545,092,868 (397,917,794, 1,306,793,042) | 1,191,011,879 (869,438,672, 6,832,749,390) |
| Nicaragua | 62,274,769 (43,592,339, 128,729,908) | 3,416,349 (2,391,445, 6,308,292) | 29,630,951 (20,741,666, 64,104,619) | 8,515,621 (5,960,935, 15,687,840) | 20,711,848 (14,498,294, 42,629,157) |
| Niger | 43,404,273 (30,382,991, 100,323,567) | 1,831,583 (1,282,108, 4,267,816) | 17,731,102 (12,411,771, 44,088,209) | 927,605 (649,324, 1,893,845) | 22,913,982 (16,039,787, 50,073,697) |
| Nigeria | 4,033,288,060 (2,944,300,284, 6,955,253,714) | 1,045,775,121 (763,415,838, 1,892,334,942) | 762,844,337 (556,876,366, 1,270,657,459) | 427,584,496 (312,136,682, 728,670,429) | 1,797,084,106 (1,311,871,397, 3,063,590,884) |
| North Macedonia | 231,218,463 (175,726,032, 414,007,208) | 20,312,360 (15,437,394, 43,757,439) | 111,438,548 (84,693,296, 193,053,400) | 38,773,884 (29,468,152, 67,614,026) | 60,693,672 (46,127,191, 109,582,342) |
| Norway | 3,012,561,019 (2,108,792,713, 8,124,729,322) | 92,170,331 (64,519,232, 259,203,422) | 1,248,443,370 (873,910,359, 3,422,928,415) | 825,526,804 (577,868,763, 1,579,647,171) | 846,420,513 (592,494,359, 2,862,950,314) |
| Oman | 108,391,564 (79,125,842, 255,906,814) | 6,090,501 (4,446,066, 14,997,955) | 34,002,360 (24,821,723, 83,566,115) | 24,271,166 (17,717,951, 52,351,776) | 44,027,537 (32,140,102, 104,990,967) |
| Pakistan | 6,076,606,294 (4,618,220,784, 9,907,709,801) | 643,478,107 (489,043,362, 1,173,505,141) | 1,818,808,890 (1,382,294,757, 2,944,472,965) | 862,358,122 (655,392,173, 1,285,811,227) | 2,751,961,175 (2,091,490,493, 4,503,920,468) |
| Panama | 2,202,135,653 (1,541,494,957, 4,515,558,641) | 39,165,886 (27,416,120, 74,681,597) | 976,371,937 (683,460,356, 2,074,300,486) | 455,856,013 (319,099,209, 808,497,534) | 730,741,817 (511,519,272, 1,558,079,025) |
| Papua New Guinea | 120,109,019 (90,061,812, 198,158,455) | 1,589,736 (1,160,507, 2,998,785) | 79,407,598 (60,349,774, 131,594,907) | 4,381,471 (3,198,474, 8,197,816) | 34,730,214 (25,353,056, 55,366,948) |
| Paraguay | 531,021,461 (387,645,667, 954,382,611) | 17,389,051 (12,694,007, 30,283,202) | 265,172,924 (193,576,235, 491,727,847) | 77,995,065 (56,936,398, 133,540,634) | 170,464,421 (124,439,027, 298,830,928) |
| Peru | 1,621,512,299 (1,135,058,609, 6,171,550,699) | 46,332,188 (32,432,531, 119,285,892) | 571,969,645 (400,378,752, 2,075,614,278) | 333,629,262 (233,540,483, 985,801,122) | 669,581,204 (468,706,843, 2,990,849,407) |
| Philippines | 4,635,930,190 (3,384,229,038, 7,311,239,326) | 68,527,681 (50,025,207, 120,273,583) | 2,755,478,849 (2,011,499,560, 4,305,437,438) | 282,434,490 (206,177,178, 444,582,360) | 1,529,489,169 (1,116,527,093, 2,440,945,946) |
| Poland | 14,973,556,495 (11,379,902,937, 33,205,588,640) | 662,663,839 (503,624,518, 3,460,968,648) | 5,952,805,052 (4,524,131,840, 11,037,451,171) | 3,864,161,063 (2,936,762,408, 8,508,796,140) | 4,493,926,541 (3,415,384,171, 10,198,372,681) |
| Portugal | 2,328,405,666 (1,699,736,136, 7,433,247,583) | 49,209,199 (35,922,715, 121,302,989) | 1,081,098,276 (789,201,741, 4,215,303,278) | 417,595,357 (304,844,611, 957,063,605) | 780,502,834 (569,767,069, 2,139,577,711) |
| Puerto Rico | 771,104,898 (585,084,935, 2,619,493,514) | 15,913,124 (11,139,187, 38,263,982) | 279,453,074 (212,384,336, 906,559,550) | 169,870,812 (129,101,817, 472,848,718) | 305,867,887 (232,459,594, 1,201,821,264) |
| Qatar | 234,932,610 (164,452,827, 482,153,091) | 7,424,199 (5,196,939, 17,516,026) | 107,107,359 (74,975,151, 216,871,170) | 48,690,003 (34,083,002, 93,733,714) | 71,711,048 (50,197,734, 154,032,180) |
| Republic of Moldova | 625,410,054 (431,532,937, 1,930,252,227) | 42,484,487 (29,314,296, 138,128,101) | 233,995,930 (161,457,192, 572,791,239) | 92,549,474 (63,859,137, 275,184,241) | 256,380,163 (176,902,313, 944,148,645) |
| Romania | 8,478,329,969 (6,189,180,878, 17,358,045,976) | 436,925,156 (318,955,364, 1,824,475,645) | 3,915,559,920 (2,858,358,742, 7,198,827,839) | 1,634,118,447 (1,192,906,467, 3,588,181,425) | 2,491,726,446 (1,818,960,305, 4,746,561,067) |
| Russian Federation | 54,253,709,059 (37,977,596,340, 135,966,591,611) | 2,610,280,967 (1,827,196,677, 8,141,002,162) | 20,886,600,725 (14,620,620,507, 57,254,926,635) | 9,070,619,571 (6,349,433,699, 21,012,060,436) | 21,686,207,796 (15,180,345,457, 49,558,602,378) |
| Rwanda | 329,382,707 (230,567,895, 749,963,132) | 22,261,955 (15,583,368, 47,432,072) | 81,972,727 (57,380,909, 176,853,407) | 45,978,289 (32,184,803, 95,622,641) | 179,169,736 (125,418,815, 430,055,013) |
| Saint Lucia | 16,932,919 (12,869,019, 33,087,594) | 477,267 (362,723, 1,101,747) | 4,340,394 (3,298,699, 8,267,282) | 4,516,928 (3,432,865, 8,900,317) | 7,598,330 (5,774,731, 14,818,248) |
| Saint Vincent and the Grenadines | NA | 359,111 (251,377, 972,181) | NA | 2,140,513 (1,498,359, 4,651,238) | 7,687,285 (5,381,100, 14,592,421) |
| Samoa | 7,998,030 (5,964,270, 15,293,017) | 641,946 (468,621, 1,420,679) | 3,781,486 (2,873,929, 6,911,961) | 408,783 (310,675, 966,186) | 3,165,814 (2,311,045, 5,994,191) |
| Sao Tome and Principe | 3,957,175 (2,770,023, 8,452,066) | NA | 593,273 (415,291, 1,316,835) | 92,124 (64,487, 291,863) | 3,271,778 (2,290,245, 6,843,346) |
| Saudi Arabia | 5,921,077,683 (4,208,545,289, 11,120,554,680) | 170,416,927 (119,291,849, 359,682,825) | 2,126,363,679 (1,552,245,486, 4,232,129,571) | 607,076,253 (424,953,377, 1,008,324,265) | 3,017,220,824 (2,112,054,577, 5,520,418,019) |
| Senegal | 126,679,852 (92,476,292, 293,975,928) | 3,626,735 (2,647,517, 6,635,540) | 36,449,335 (26,608,014, 111,402,085) | 4,173,399 (3,046,582, 8,181,981) | 82,430,382 (60,174,179, 167,756,322) |
| Serbia | 1,238,403,723 (904,034,717, 2,453,123,173) | 59,660,335 (43,552,045, 117,783,372) | 545,660,349 (398,332,055, 1,102,063,536) | 286,978,630 (209,494,400, 516,315,039) | 346,104,409 (252,656,219, 716,961,227) |
| Seychelles | 45,564,899 (34,629,323, 95,090,898) | 880,333 (669,053, 2,672,397) | 20,386,932 (15,494,068, 41,101,130) | 7,321,735 (5,564,518, 16,308,732) | 16,975,900 (12,901,684, 35,008,640) |
| Sierra Leone | 19,363,897 (13,926,588, 37,847,646) | 465,956 (340,148, 868,580) | 6,487,161 (4,541,012, 15,484,146) | 481,380 (336,966, 1,068,010) | 11,929,400 (8,708,462, 20,426,909) |
| Singapore | 2,063,597,413 (1,444,518,189, 4,165,384,319) | 45,117,080 (31,581,956, 174,377,396) | 972,539,718 (680,777,802, 1,848,018,740) | 173,416,469 (121,391,528, 290,374,639) | 872,524,146 (610,766,902, 1,852,613,545) |
| Slovakia | 1,586,704,071 (1,205,895,094, 2,689,528,657) | 76,879,996 (58,428,797, 130,920,350) | 687,678,730 (522,635,835, 1,132,189,967) | 414,544,466 (315,053,794, 699,025,839) | 407,600,879 (309,776,668, 727,392,501) |
| Slovenia | 660,011,806 (501,608,972, 1,761,321,276) | 10,411,019 (7,912,374, 20,311,267) | 198,053,180 (150,520,417, 523,173,911) | 183,116,196 (139,168,309, 365,492,111) | 268,431,411 (204,007,872, 852,343,987) |
| Solomon Islands | 4,159,810 (3,161,455, 7,660,026) | 64,099 (48,715, 163,223) | 2,503,519 (1,902,674, 4,502,084) | 186,625 (141,835, 429,235) | 1,405,568 (1,068,231, 2,565,484) |
| Somalia | 223,541,664 (156,479,165, 414,438,510) | 31,318,434 (21,922,904, 59,212,810) | 64,154,098 (44,907,869, 122,811,276) | 9,851,543 (6,896,080, 16,792,202) | 118,217,589 (82,752,312, 215,622,221) |
| Spain | 8,613,276,068 (6,029,293,248, 16,509,571,646) | 324,260,903 (226,982,632, 1,057,355,690) | 3,777,315,995 (2,644,121,197, 6,314,109,730) | 1,619,061,463 (1,133,343,024, 3,390,562,475) | 2,892,637,707 (2,024,846,395, 5,747,543,751) |
| Sri Lanka | 3,866,407,314 (2,706,485,120, 23,876,509,408) | 38,658,713 (27,061,099, 109,881,870) | 2,914,266,187 (2,039,986,331, 19,466,569,343) | 134,151,472 (93,906,030, 293,214,208) | 779,330,942 (545,531,659, 4,006,843,987) |
| Sudan | 149,733,445 (109,305,415, 224,314,007) | 6,390,025 (4,664,718, 10,841,836) | 90,083,712 (65,761,110, 129,595,615) | 10,740,550 (7,840,601, 17,646,036) | 42,519,158 (31,038,986, 66,230,520) |
| Suriname | 40,664,258 (28,058,338, 78,290,535) | 2,147,283 (1,481,625, 4,374,411) | 16,563,624 (11,428,901, 31,960,068) | 6,681,861 (4,610,484, 12,708,913) | 15,271,490 (10,537,328, 29,247,144) |
| Sweden | 2,738,406,178 (2,263,439,351, 7,130,866,016) | 49,081,284 (37,301,775, 238,709,289) | 914,161,244 (694,762,546, 3,219,487,310) | 837,171,550 (818,501,035, 1,460,669,331) | 937,992,100 (712,873,996, 2,212,000,086) |
| Switzerland | 3,161,913,408 (2,214,288,573, 11,759,906,441) | 31,639,582 (23,096,895, 85,797,684) | 1,425,375,426 (997,762,798, 5,123,614,390) | 701,311,330 (490,917,931, 2,623,597,880) | 1,003,587,070 (702,510,949, 3,926,896,487) |
| Tajikistan | 163,317,317 (124,121,161, 419,957,715) | 3,141,363 (2,387,436, 7,055,819) | 95,926,744 (72,904,325, 225,715,002) | 6,780,411 (5,153,113, 16,014,819) | 57,468,799 (43,676,287, 171,172,075) |
| Thailand | 13,371,205,287 (9,760,979,859, 30,146,296,935) | 130,531,186 (95,287,766, 324,978,349) | 7,730,490,009 (5,643,257,706, 14,520,067,823) | 718,719,016 (524,664,882, 1,655,351,310) | 4,791,465,076 (3,497,769,505, 13,645,899,453) |
| Timor-Leste | 38,285,009 (27,948,057, 95,087,211) | 1,267,115 (924,994, 3,389,511) | 25,255,023 (18,436,167, 63,244,262) | 1,118,181 (816,272, 2,417,577) | 10,644,690 (7,770,624, 26,035,861) |
| Togo | 47,030,577 (34,332,321, 79,837,108) | 1,062,272 (775,458, 2,134,790) | 14,984,078 (10,938,377, 27,503,860) | 1,278,456 (933,273, 2,749,992) | 29,705,772 (21,685,213, 47,448,467) |
| Tonga | 6,253,289 (4,752,499, 12,700,056) | NA | 2,099,603 (1,595,699, 4,165,955) | 1,184,905 (900,528, 2,636,666) | 2,968,780 (2,256,273, 5,897,415) |
| Trinidad and Tobago | 169,493,445 (118,645,411, 374,221,856) | 4,352,944 (3,047,061, 9,569,228) | 63,080,335 (44,156,235, 128,579,229) | 39,970,688 (27,979,481, 74,408,416) | 62,089,478 (43,462,635, 161,664,984) |
| Tunisia | 327,998,850 (229,599,195, 499,065,641) | 23,452,822 (16,416,975, 37,196,652) | 95,368,678 (66,758,075, 144,714,972) | 54,817,447 (38,372,213, 86,773,488) | 154,359,903 (108,051,932, 230,380,529) |
| Turkey | 37,567,862,599 (27,424,539,697, 73,547,145,387) | 793,399,418 (579,181,575, 1,562,020,714) | 18,043,081,173 (13,171,449,256, 35,078,127,493) | 7,230,088,563 (5,277,964,651, 13,622,151,373) | 11,501,293,445 (8,395,944,215, 23,284,845,807) |
| Uganda | 645,391,823 (490,497,785, 1,293,694,531) | 44,363,316 (33,716,120, 97,369,430) | 78,482,466 (59,646,674, 151,440,350) | 122,622,509 (93,193,107, 214,794,577) | 399,923,531 (303,941,884, 830,090,174) |
| Ukraine | 8,985,256,726 (6,803,226,142, 28,183,356,515) | 881,381,174 (669,849,692, 3,020,434,443) | 4,358,072,288 (3,312,134,939, 14,131,487,641) | 852,299,019 (622,178,284, 2,377,013,097) | 2,893,504,245 (2,199,063,227, 8,654,421,334) |
| United Arab Emirates | 1,204,627,726 (879,378,240, 2,979,484,565) | 41,415,479 (30,233,300, 96,471,328) | 492,851,656 (359,781,709, 1,236,212,393) | 283,412,292 (206,890,973, 709,050,298) | 386,948,299 (282,472,259, 937,750,546) |
| United Kingdom | 23,085,491,075 (16,852,408,485, 53,076,301,682) | 1,341,785,890 (979,503,700, 6,958,815,867) | 8,559,843,426 (6,248,685,701, 16,393,923,451) | 5,195,519,381 (3,792,729,148, 11,466,527,054) | 7,988,342,378 (5,831,489,936, 18,257,035,310) |
| United Republic of Tanzania | 1,516,477,068 (1,152,522,572, 2,457,992,207) | 96,698,681 (73,490,998, 162,427,106) | 385,034,584 (292,626,284, 646,562,411) | 227,986,511 (173,269,748, 356,671,156) | 806,757,292 (613,135,542, 1,292,331,534) |
| United States of America | 208,686,327,548 (146,080,429,284, 370,010,663,277) | 4,603,569,015 (3,222,498,310, 9,135,675,007) | 83,997,949,627 (58,798,564,739, 135,952,000,000) | 45,999,038,767 (32,199,327,137, 84,848,988,270) | 74,085,770,139 (51,860,039,098, 140,074,000,000) |
| Uruguay | 1,293,994,709 (905,796,296, 2,758,779,555) | 43,449,744 (30,414,821, 185,770,479) | 505,201,452 (353,641,016, 1,198,577,666) | 243,262,931 (170,284,052, 408,118,460) | 502,080,583 (351,456,408, 966,312,950) |
| Uzbekistan | 647,143,976 (491,829,421, 2,023,421,292) | 43,717,237 (33,225,100, 155,513,883) | 404,519,822 (307,435,065, 1,422,516,272) | 52,901,272 (40,204,967, 118,893,823) | 146,005,645 (110,964,290, 326,497,314) |
| Vanuatu | 2,497,733 (1,870,005, 5,028,070) | 54,781 (39,990, 349,252) | 1,419,157 (1,078,560, 2,599,502) | 136,168 (103,487, 352,843) | 887,627 (647,968, 1,726,473) |
| VietNam | 7,611,542,175 (6,184,162,451, 10,311,596,575) | 517,778,034 (393,511,306, 791,941,653) | 5,801,505,526 (4,409,144,200, 8,628,627,607) | 637,437,211 (484,452,280, 1,000,705,658) | 7,759,282,454 (5,897,054,665, 11,890,321,657) |
| Zambia | 155,704,812 (108,993,369, 300,988,278) | 8,589,632 (6,012,742, 16,331,560) | 31,904,622 (22,333,235, 56,605,422) | 27,714,582 (19,400,208, 62,947,677) | 87,495,977 (61,247,184, 165,103,619) |
| Zimbabwe | 239,291,715 (181,861,704, 1,095,444,140) | 12,193,829 (9,267,310, 49,008,471) | 59,100,671 (44,916,510, 238,877,480) | 30,239,572 (22,982,075, 99,106,946) | 137,757,643 (104,695,809, 708,451,243) |

Note: GDP, Gross Domestic Product

# Table S4 Projected Economic Burden of Hematologic Malignancies in Various Countries, 2050 (Discounted to 2021 US$)

| Country | Hematologic Malignancies Economic Burden ($) | Hodgkin lymphoma Economic Burden ($) | Leukemia Economic Burden ($) | Multiple myeloma Economic Burden ($) | Non-Hodgkin lymphoma Economic Burden ($) |
| --- | --- | --- | --- | --- | --- |
| Afghanistan | 1,133,121,864 (861,172,617, 2,843,071,954) | 67,853,340 (51,568,539, 167,469,903) | 694,475,669 (527,801,509, 1,819,377,638) | 61,969,703 (47,096,974, 158,629,297) | 308,823,152 (234,705,595, 697,595,117) |
| Albania | 98,741,092 (74,299,651, 359,203,456) | 5,389,150 (3,934,079, 17,261,932) | 53,140,595 (40,386,852, 218,026,041) | 20,814,522 (15,819,037, 51,901,442) | 19,396,826 (14,159,683, 72,014,041) |
| Algeria | 467,905,557 (327,533,890, 906,935,075) | 34,289,544 (24,002,681, 68,078,869) | 147,517,022 (103,261,915, 284,737,362) | 83,754,463 (58,628,124, 167,289,690) | 202,344,528 (141,641,170, 386,829,154) |
| Andorra | 11,157,881 (8,032,134, 32,693,788) | 7,665 (5,365, 74,665) | 111,457,901 (84,708,005, 288,065,514) | 34,360,137 (25,082,900, 84,506,716) | 207,840,630 (151,723,660, 527,397,416) |
| Angola | 367,487,540 (271,609,641, 931,524,569) | 13,828,873 (10,095,077, 31,554,922) | 2,722,812 (1,987,652, 7,444,212) | 1,943,088 (1,418,454, 5,715,083) | 2,258,204 (1,648,489, 6,495,614) |
| Antigua and Barbuda | 6,924,108 (5,054,599, 19,655,583) | 4 (3, 674) | 717,208,337 (545,078,336, 2,500,614,420) | 357,882,804 (271,990,931, 1,007,485,607) | 687,855,474 (502,134,496, 2,044,767,169) |
| Argentina | 1,817,170,028 (1,358,786,855, 6,086,141,929) | 54,223,414 (39,583,092, 533,274,733) | 231,980,964 (176,305,532, 1,769,387,507) | 184,117,320 (139,929,163, 934,596,734) | 118,779,677 (90,272,554, 943,029,282) |
| Armenia | 554,121,903 (421,132,646, 3,797,474,360) | 19,243,942 (14,625,396, 150,460,838) | 4,371,568,251 (661,035,696, 16,913,096,496) | 3,206,254,741 (2,212,315,771, 15,853,548,419) | 4,497,591,980 (661,035,696, 11,634,098,106) |
| Australia | 12,302,660,241 (3,707,093,566, 45,169,864,207) | 227,245,269 (172,706,404, 769,121,186) | 776,845,081 (536,023,106, 2,459,094,339) | 472,138,861 (325,775,814, 1,314,989,946) | 598,938,158 (413,267,329, 1,855,804,433) |
| Austria | 1,906,015,356 (2,120,293,537, 5,921,825,054) | 58,093,256 (845,227,288, 291,936,336) | 126,750,828 (96,330,630, 532,133,187) | 9,505,643 (7,224,289, 40,853,141) | 35,305,906 (26,832,488, 205,690,237) |
| Azerbaijan | 187,716,777 (142,664,751, 851,346,028) | 16,154,400 (12,277,344, 72,669,463) | 7,349,973 (5,144,981, 17,893,794) | 10,232,949 (7,163,064, 23,699,678) | 10,410,834 (7,287,584, 25,108,229) |
| Bahamas | 28,697,583 (20,088,308, 68,868,808) | 703,827 (492,679, 2,167,107) | 196,921,181 (137,844,827, 527,480,458) | 89,255,095 (62,478,566, 221,072,099) | 143,904,738 (100,733,317, 456,538,639) |
| Bahrain | 444,661,276 (311,262,893, 1,251,118,035) | 14,580,262 (10,206,183, 46,026,838) | 10,926,232,122 (7,976,149,449, 30,863,717,598) | 6,121,926,632 (4,469,006,441, 15,283,867,781) | 9,362,830,722 (6,834,866,427, 30,070,614,396) |
| Bangladesh | 27,836,815,511 (20,320,875,323, 79,861,399,644) | 1,425,826,035 (1,040,853,006, 3,643,199,869) | 4,000,527 (2,920,385, 11,405,120) | 1,952,317 (1,425,192, 4,857,948) | 5,160,458 (3,767,134, 14,168,685) |
| Barbados | 11,336,691 (8,275,784, 31,163,667) | 223,388 (163,073, 731,914) | 1,905,927,409 (1,315,089,912, 7,945,383,139) | 648,447,643 (447,428,873, 2,974,162,848) | 1,802,512,159 (1,243,733,390, 11,295,998,230) |
| Belarus | 4,661,383,455 (3,216,354,584, 23,790,566,055) | 304,496,244 (210,102,409, 1,575,021,838) | 3,271,900,909 (2,388,487,664, 15,883,529,262) | 920,870,167 (699,861,327, 2,243,981,883) | 1,647,461,869 (1,252,071,020, 5,562,319,969) |
| Belgium | 5,927,981,905 (4,407,109,221, 23,950,287,441) | 87,748,960 (66,689,210, 260,456,327) | 1,851,146 (1,351,336, 5,084,991) | 512,879 (374,401, 1,565,108) | 1,378,703 (1,006,453, 3,555,716) |
| Belize | 3,935,992 (2,873,274, 10,851,102) | 193,264 (141,083, 645,286) | 53,067,881 (37,147,517, 133,196,160) | 4,405,304 (3,083,713, 12,228,403) | 90,297,888 (63,208,521, 210,498,368) |
| Benin | 150,312,493 (105,218,745, 364,347,282) | 2,541,420 (1,778,994, 8,424,350) | 12,646,419 (9,611,278, 39,087,364) | 13,330,050 (10,130,838, 39,165,250) | 11,679,212 (8,876,201, 36,000,126) |
| Bermuda | 37,655,713 (28,618,342, 114,258,820) | 33 (25, 6,079) | 63,438,933 (44,407,253, 156,719,954) | 43,029,028 (30,120,320, 112,979,165) | 55,192,340 (38,634,638, 140,862,222) |
| Bhutan | 169,287,244 (118,501,070, 430,205,993) | 7,626,943 (5,338,860, 19,644,652) | 1,273,748,796 (968,049,085, 2,346,437,277) | 417,518,604 (317,314,139, 883,924,415) | 1,207,038,217 (917,349,045, 2,484,476,588) |
| Bolivia (Plurinational State of) | 2,976,753,270 (2,262,332,485, 5,887,080,171) | 78,447,653 (59,620,216, 172,241,891) | 123,486,345 (93,849,622, 442,220,138) | 129,473,874 (98,400,144, 335,987,046) | 90,319,276 (68,642,649, 404,961,533) |
| Bosnia and Herzegovina | 362,123,335 (275,213,735, 1,258,721,136) | 18,843,841 (14,321,319, 75,552,420) | 72,609,484 (50,826,639, 265,720,408) | 33,767,856 (23,637,499, 104,513,782) | 103,414,510 (72,390,157, 519,935,850) |
| Botswana | 214,791,405 (150,353,984, 907,909,049) | 4,999,556 (3,499,689, 17,739,010) | 2,617,362,187 (1,832,153,531, 5,987,203,449) | 1,857,148,980 (1,300,004,286, 7,254,755,185) | 2,545,245,599 (1,781,671,919, 6,213,832,536) |
| Brazil | 7,207,438,075 (5,045,206,653, 20,220,066,482) | 187,681,309 (131,376,917, 764,275,312) | 54,952,989 (41,764,272, 143,118,009) | 21,330,364 (16,211,076, 57,477,830) | 57,941,190 (44,035,304, 168,497,918) |
| Brunei Darussalam | 138,302,054 (105,109,561, 382,084,955) | 4,077,512 (3,098,909, 12,991,199) | 1,650,320,460 (1,138,721,117, 15,375,704,860) | 592,759,179 (409,003,833, 1,784,005,099) | 744,043,565 (513,390,060, 2,649,498,192) |
| Bulgaria | 3,117,197,408 (2,150,866,211, 20,414,894,282) | 130,074,204 (89,751,201, 605,686,131) | 65,343,324 (45,740,327, 213,985,827) | 8,851,335 (6,195,935, 23,217,885) | 125,460,705 (87,822,493, 363,362,581) |
| Burkina Faso | 205,174,080 (143,621,856, 620,658,830) | 5,518,716 (3,863,101, 20,092,538) | 6,389,521 (4,472,664, 14,642,551) | 2,631,557 (1,842,090, 7,099,540) | 15,323,672 (10,726,571, 39,117,673) |
| Burundi | 26,696,290 (18,663,888, 65,989,568) | 2,351,541 (1,622,563, 5,129,804) | 1,679,041 (1,175,329, 4,238,094) | 773,267 (541,287, 3,069,646) | 1,946,991 (1,362,893, 11,711,375) |
| Cabo Verde | 4,399,299 (3,079,509, 19,019,120) | NA | 821,438,904 (575,007,232, 1,559,435,925) | 97,165,687 (68,015,981, 227,681,923) | 568,195,217 (397,736,652, 1,235,526,936) |
| Cambodia | 1,512,505,993 (1,058,754,195, 3,080,232,905) | 25,706,186 (17,994,330, 57,588,120) | 103,273,266 (78,487,682, 252,316,809) | 19,321,533 (14,684,365, 49,223,396) | 307,943,327 (234,036,928, 661,703,336) |
| Cameroon | 436,871,539 (332,022,369, 982,113,629) | 6,333,413 (4,813,394, 18,870,087) | 3,169,143,773 (2,313,474,954, 7,856,723,606) | 1,805,684,161 (1,318,149,437, 4,192,039,403) | 3,199,992,676 (2,239,994,873, 7,878,025,831) |
| Canada | 8,327,246,547 (5,978,317,420, 20,362,728,321) | 152,425,937 (106,698,156, 435,939,481) | 1,474,613 (1,032,229, 3,014,897) | 162,426 (113,698, 397,521) | 1,976,607 (1,383,625, 3,954,373) |
| Central African Republic | 3,940,270 (2,758,189, 8,090,527) | 326,624 (228,637, 723,736) | 6,433,137 (4,696,190, 15,410,098) | 479,522 (350,051, 1,345,012) | 9,879,594 (7,212,104, 21,985,297) |
| Chad | 17,289,702 (12,621,483, 40,191,630) | 497,449 (363,138, 1,451,223) | 765,862,437 (559,079,579, 2,303,157,853) | 528,429,006 (385,753,174, 2,317,159,501) | 844,493,307 (616,480,114, 2,552,450,461) |
| Chile | 2,303,078,580 (1,681,247,363, 10,933,216,585) | 164,293,829 (119,934,496, 3,760,448,770) | 192,199,000,000 (140,305,000,000, 421,440,000,000) | 70,616,405,457 (53,668,468,147, 493,542,000,000) | 152,543,000,000 (115,933,000,000, 560,880,000,000) |
| China | 421,645,562,182 (314,684,707,258, 1,495,354,203,764) | 6,287,156,725 (4,778,239,111, 19,492,203,764) | 1,834,631,084 (1,284,241,759, 6,532,204,493) | 2,230,628,905 (1,561,440,233, 8,699,873,290) | 2,205,574,391 (1,543,902,074, 7,966,515,128) |
| Colombia | 6,458,974,268 (4,521,281,987, 25,176,476,634) | 188,139,888 (131,697,921, 1,977,883,723) | 3,867,714 (2,707,400, 18,813,408) | 1,625,035 (1,137,524, 4,274,212) | 7,364,414 (5,155,090, 33,701,235) |
| Comoros | 14,333,171 (10,033,219, 62,398,179) | 1,476,008 (1,033,205, 5,609,324) | 9,049,581 (6,877,682, 25,140,080) | 3,374,402 (2,564,546, 8,422,639) | 20,656,582 (15,699,003, 62,897,366) |
| Congo | 34,275,632 (26,013,628, 99,383,529) | 1,195,066 (872,398, 2,923,443) | 1,948,872,029 (1,364,210,421, 14,526,384,800) | 810,408,094 (567,285,666, 3,943,613,263) | 1,570,842,808 (1,099,589,965, 7,577,561,334) |
| Costa Rica | 4,440,647,314 (3,108,453,120, 26,392,290,536) | 110,524,384 (77,367,069, 344,731,139) | 702,350,876 (533,786,665, 2,622,679,712) | 1,883,824,275 (1,431,706,449, 4,411,340,483) | 4,553,067,233 (3,460,331,097, 15,391,675,505) |
| Coted' Ivoire | 7,438,747,419 (5,653,448,038, 23,518,716,589) | 299,505,035 (227,623,827, 1,093,020,889) | 506,985,956 (370,099,748, 2,078,544,396) | 317,290,465 (231,622,039, 1,442,690,916) | 509,394,945 (371,858,310, 4,137,367,303) |
| Croatia | 1,357,557,403 (991,016,905, 7,947,997,050) | 23,886,038 (17,436,808, 289,394,435) | 57,990,899 (42,333,356, 133,673,297) | 30,392,252 (22,186,344, 72,849,045) | 48,738,841 (35,579,354, 112,184,354) |
| Cyprus | 141,886,901 (103,577,438, 331,769,279) | 4,764,909 (3,478,384, 13,062,583) | 1,845,458,684 (1,273,366,492, 5,571,879,412) | 1,255,216,035 (866,099,064, 4,124,685,511) | 1,818,667,314 (1,254,880,447, 4,712,807,190) |
| Czechia | 5,025,235,036 (3,467,412,175, 14,656,148,974) | 105,893,003 (73,066,172, 246,776,861) | 358,307,634 (261,564,573, 765,436,743) | 98,778,607 (72,108,383, 257,496,298) | 735,182,763 (536,683,417, 1,614,883,349) |
| Democratic Republic of the Congo | 1,255,083,767 (916,211,150, 2,772,823,618) | 62,814,764 (45,854,778, 135,007,227) | 1,468,475,674 (1,116,041,512, 5,618,757,980) | 703,742,229 (534,844,094, 4,231,705,579) | 804,421,877 (611,360,626, 4,745,095,340) |
| Denmark | 2,997,665,668 (2,278,225,907, 14,675,790,903) | 21,025,889 (15,979,675, 80,232,004) | 5,352,327 (3,746,629, 14,299,967) | 1,630,860 (1,141,602, 4,757,173) | 5,360,898 (3,752,629, 14,176,113) |
| Djibouti | 14,582,574 (11,082,756, 34,780,127) | 778,912 (591,973, 2,037,356) | 976,897,742 (713,135,352, 2,390,722,632) | 699,729,486 (510,802,525, 1,682,921,777) | 591,732,273 (414,212,591, 2,030,718,453) |
| Dominica | 12,597,166 (8,818,016, 34,081,363) | 253,081 (177,157, 848,110) | 200,689,206 (138,475,552, 1,408,135,900) | 92,705,874 (63,967,053, 1,144,982,263) | 225,741,801 (155,761,843, 1,385,767,214) |
| Dominican Republic | 2,277,632,141 (1,644,641,315, 6,131,760,205) | 9,272,641 (6,490,848, 27,397,343) | 16,618,568,550 (12,630,112,098, 62,497,214,458) | 1,527,364,554 (1,160,797,061, 3,328,583,986) | 4,897,190,934 (3,721,865,110, 15,425,191,854) |
| Ecuador | 542,323,259 (375,826,095, 4,351,710,833) | 23,186,378 (17,621,648, 412,825,456) | 171,255,883 (125,016,794, 688,004,462) | 33,061,756 (24,135,082, 77,267,912) | 97,907,599 (71,472,547, 624,601,976) |
| Egypt | 23,195,319,236 (17,628,442,620, 81,703,436,025) | 152,195,198 (115,668,351, 452,445,727) | 3,571,897 (2,714,642, 9,679,720) | 1,420,105 (1,079,280, 3,896,801) | 7,810,221 (5,935,768, 24,911,120) |
| El Salvador | 312,911,327 (228,425,269, 1,418,795,152) | 10,686,090 (7,800,846, 28,920,801) | 335,133,840 (244,647,703, 1,307,480,791) | 404,519,252 (295,299,054, 1,983,058,183) | 559,201,898 (408,217,385, 4,031,293,946) |
| Equatorial Guinea | 12,989,842 (9,872,280, 39,060,164) | 187,620 (142,591, 572,522) | 12,536,932 (9,151,961, 40,180,060) | 6,663,222 (4,864,152, 20,079,123) | 17,476,890 (13,282,436, 73,003,766) |
| Estonia | 1,309,841,123 (956,184,019, 7,364,341,305) | 10,986,133 (8,019,877, 42,508,385) | 17,830,210,805 (13,016,053,888, 36,374,302,011) | 2,707,782,529 (2,057,914,722, 6,478,518,752) | 12,439,101,745 (9,453,717,326, 27,369,116,866) |
| Eswatini | 37,915,376 (28,239,681, 137,439,754) | 1,238,332 (941,133, 4,176,806) | 61,557,556 (46,783,742, 146,911,167) | 6,871,794 (5,222,563, 18,657,330) | 18,098,709 (13,755,019, 54,655,829) |
| Ethiopia | 35,236,805,568 (26,245,065,908, 75,216,851,454) | 2,259,710,489 (1,717,379,972, 4,994,913,825) | 780,496,097 (569,762,151, 2,821,062,846) | 382,804,040 (290,931,071, 1,039,204,594) | 902,098,876 (685,595,146, 2,333,293,933) |
| Fiji | 88,356,589 (67,151,008, 228,173,980) | 1,828,531 (1,389,683, 7,949,654) | 9,298,149,525 (6,508,704,668, 23,311,191,136) | 3,224,264,253 (2,256,984,977, 11,245,984,135) | 6,123,660,215 (4,286,562,151, 19,483,286,098) |
| Finland | 2,088,535,060 (1,563,871,762, 6,281,767,091) | 23,136,046 (17,583,395, 88,205,718) | 21,703,633 (16,494,761, 48,779,897) | 16,556,010 (12,582,567, 40,491,625) | 52,209,656 (39,679,339, 116,314,728) |
| France | 19,060,270,785 (13,342,189,550, 55,353,819,901) | 414,196,792 (289,937,754, 1,313,358,532) | 1,137,070 (795,949, 3,865,926) | 161,368 (112,957, 496,187) | 4,768,500 (3,337,950, 23,162,297) |
| Gabon | 92,967,864 (70,580,620, 211,942,601) | 2,498,565 (1,823,953, 6,356,351) | 475,544,756 (328,125,882, 3,182,542,801) | 269,807,692 (186,167,307, 5,810,730,632) | 337,966,619 (233,196,967, 4,764,304,285) |
| Gambia | 6,774,121 (4,741,885, 29,778,005) | 707,184 (495,028, 2,253,595) | 18,697,364,475 (14,209,997,001, 49,911,718,752) | 5,343,718,339 (4,061,225,938, 17,357,956,485) | 11,293,918,767 (8,583,378,263, 125,779,000,000) |
| Georgia | 1,241,215,903 (856,438,973, 15,713,793,039) | 157,896,835 (108,948,816, 1,956,215,321) | 237,874,103 (166,511,872, 1,223,023,107) | 52,734,872 (36,914,410, 133,655,388) | 668,805,633 (468,163,943, 3,836,550,099) |
| Germany | 36,099,634,415 (27,435,722,156, 197,921,864,517) | 764,632,834 (581,120,954, 4,873,189,280) | 641,934,356 (449,354,049, 1,824,752,157) | 292,654,046 (204,857,832, 757,387,538) | 306,035,533 (223,405,939, 1,705,648,755) |
| Ghana | 962,897,948 (674,028,563, 5,213,492,424) | 3,483,340 (2,438,338, 20,263,831) | 3,854,267 (2,697,987, 10,574,614) | 2,940,373 (2,058,261, 8,461,205) | 7,672,122 (5,370,485, 19,918,561) |
| Greece | 1,360,560,188 (965,171,285, 5,119,462,859) | 119,936,253 (87,553,465, 831,674,409) | 682,782,958 (477,948,071, 4,568,983,527) | 192,191,995 (134,534,397, 450,765,746) | 288,335,381 (201,834,767, 2,109,632,337) |
| Grenada | 14,581,228 (10,206,860, 39,351,051) | 114,467 (80,127, 396,672) | 30,852,544 (21,596,781, 79,072,439) | 22,917,645 (16,042,352, 56,880,694) | 185,645,157 (129,951,610, 408,060,089) |
| Guatemala | 1,198,537,005 (838,975,904, 7,227,464,343) | 35,226,671 (24,658,670, 98,082,733) | 5,601,954 (4,089,426, 14,241,282) | 1,061,339 (774,778, 3,685,975) | 14,699,067 (10,730,319, 32,839,192) |
| Guinea | 275,672,864 (192,971,005, 621,920,678) | 36,257,518 (25,380,263, 77,907,456) | 108,806,069 (79,428,430, 651,135,536) | 50,710,487 (37,018,655, 422,999,708) | 120,613,373 (88,047,763, 419,973,676) |
| Guinea-Bissau | 21,776,087 (15,896,544, 51,878,298) | 413,727 (302,021, 1,111,849) | 48,481,167 (33,936,817, 116,148,579) | 8,648,134 (6,053,694, 18,429,849) | 37,081,744 (25,957,221, 85,059,601) |
| Guyana | 296,273,008 (216,279,296, 1,540,638,596) | 16,143,078 (11,784,447, 46,529,676) | 155,766,716 (109,036,701, 321,088,824) | 34,638,767 (24,247,137, 76,908,036) | 49,403,642 (34,582,550, 107,001,615) |
| Haiti | 99,774,343 (69,842,040, 232,216,794) | 5,563,299 (3,894,309, 12,578,765) | 1,398,778,538 (1,063,071,689, 3,118,195,028) | 705,608,927 (536,262,785, 2,554,466,320) | 798,905,895 (607,168,480, 3,267,205,955) |
| Honduras | 245,891,750 (172,124,225, 519,422,731) | 6,082,624 (4,257,837, 14,424,256) | 136,126,831 (103,456,391, 322,651,551) | 60,918,249 (46,297,869, 151,660,595) | 158,430,685 (120,407,320, 394,809,785) |
| Hungary | 2,963,217,137 (2,252,045,024, 9,181,968,980) | 59,923,777 (45,542,070, 242,101,677) | 32,048,354,682 (23,395,298,918, 69,787,213,418) | 19,028,742,217 (13,890,981,818, 42,516,275,455) | 27,568,797,182 (20,125,221,943, 71,782,710,354) |
| Iceland | 361,449,963 (274,701,972, 888,750,667) | 5,974,198 (4,540,391, 19,628,736) | 9,860,874,222 (7,198,438,182, 17,644,247,279) | 1,250,420,464 (912,806,938, 2,339,087,841) | 5,142,270,099 (3,753,857,172, 9,469,429,510) |
| India | 81,951,399,094 (59,824,521,338, 191,618,408,070) | 3,305,505,013 (2,413,018,659, 7,532,208,843) | 143,942,019 (100,759,413, 312,218,405) | 23,513,729 (16,459,610, 66,987,968) | 51,296,870 (35,907,809, 137,528,893) |
| Indonesia | 16,553,675,039 (12,093,186,085, 29,996,615,768) | 300,110,254 (228,083,793, 543,851,138) | 287,821,445 (201,475,012, 600,047,434) | 130,516,892 (91,361,824, 288,995,676) | 163,486,108 (114,440,275, 406,701,582) |
| Iran (Islamic Republic of) | 224,367,324 (157,057,127, 546,575,423) | 5,614,706 (3,930,295, 29,840,157) | 826,859,819 (603,607,668, 2,870,834,018) | 983,276,629 (717,791,939, 2,950,399,452) | 844,686,531 (616,621,168, 3,697,628,743) |
| Iraq | 592,402,907 (414,682,035, 1,331,643,715) | 10,578,463 (7,404,924, 35,899,025) | 570,147,800 (393,401,982, 2,331,849,624) | 524,613,457 (382,967,823, 2,038,801,686) | 758,577,944 (553,761,899, 3,169,359,116) |
| Ireland | 2,756,176,979 (2,012,009,195, 9,841,822,293) | 101,354,000 (73,988,420, 322,960,080) | 4,532,959,183 (3,445,048,979, 11,388,419,980) | 2,981,354,407 (2,265,829,349, 10,560,729,663) | 4,309,699,265 (3,275,371,441, 15,339,254,866) |
| Israel | 1,895,873,604 (1,361,181,819, 7,641,025,734) | 42,534,404 (31,050,115, 101,015,308) | 22,254,227 (16,245,586, 268,834,478) | 17,470,859 (12,753,727, 402,320,023) | 41,111,445 (30,011,355, 1,044,486,617) |
| Italy | 12,490,373,179 (9,492,683,615, 42,390,219,144) | 666,360,324 (506,433,846, 5,101,814,635) | 5,844,404,036 (4,091,082,825, 11,714,500,342) | 2,491,826,271 (1,744,278,390, 5,386,317,472) | 6,809,721,577 (4,766,805,104, 16,722,559,726) |
| Jamaica | 82,002,760 (59,862,015, 1,721,079,013) | 1,166,229 (851,347, 5,437,896) | 73,073,122 (53,343,379, 219,266,814) | 11,640,427 (8,497,511, 29,732,646) | 54,550,451 (39,821,829, 171,966,961) |
| Japan | 15,399,760,278 (10,779,832,195, 37,489,858,496) | 253,808,394 (177,665,876, 3,666,480,956) | 1,576,694,624 (1,198,287,914, 7,628,737,204) | 686,580,309 (521,801,035, 5,014,194,961) | 1,065,571,300 (809,834,188, 14,272,732,331) |
| Jordan | 140,977,435 (102,913,527, 426,693,455) | 1,713,435 (1,250,808, 5,727,034) | 1,427,125,596 (998,987,917, 3,648,660,011) | 1,504,305,271 (1,053,013,690, 3,307,932,322) | 2,719,264,290 (1,903,485,003, 7,160,602,925) |
| Kazakhstan | 3,550,796,708 (2,698,605,498, 28,278,493,162) | 221,950,475 (168,682,361, 1,362,828,666) | 4,835,532 (3,529,938, 12,395,589) | 214,045 (156,253, 880,174) | 1,289,289 (941,181, 4,354,044) |
| Kenya | 5,894,849,773 (4,126,394,841, 14,779,088,023) | 244,154,616 (170,908,231, 661,892,765) | 56,484,933 (41,234,001, 393,839,236) | 9,949,292 (7,262,983, 118,041,126) | 94,751,308 (69,168,455, 2,831,521,864) |
| Kiribati | 6,338,865 (4,627,372, 17,629,848) | NA | 172,317,607 (118,899,149, 814,426,667) | 42,005,952 (28,984,107, 232,134,562) | 63,403,573 (43,748,466, 618,308,600) |
| Kuwait | 166,368,030 (121,448,662, 3,574,902,851) | 5,182,497 (3,783,223, 231,500,625) | 1,101,463,614 (771,024,530, 2,100,245,973) | 124,332,378 (90,762,636, 293,928,067) | 625,335,088 (456,494,614, 1,257,782,543) |
| Kyrgyzstan | 296,193,592 (204,373,579, 2,015,605,292) | 18,466,460 (12,741,857, 350,735,464) | 311,622,782 (236,833,314, 2,171,842,068) | 323,028,611 (245,501,745, 1,574,152,527) | 341,613,268 (259,626,084, 1,989,951,558) |
| Lao People's Democratic Republic | 1,884,519,716 (1,342,655,485, 3,729,414,120) | 33,388,637 (24,373,705, 77,457,537) | 93,868,053 (68,523,679, 199,004,407) | 47,362,444 (35,995,458, 128,373,452) | 169,070,384 (128,493,492, 653,039,968) |
| Latvia | 999,025,032 (759,259,024, 5,820,044,657) | 22,760,371 (17,297,882, 84,098,504) | 4,020,738 (2,814,516, 15,204,524) | 1,493,193 (1,045,235, 4,400,276) | 4,987,321 (3,491,125, 23,576,835) |
| Lebanon | 319,892,645 (240,302,368, 1,003,688,268) | 9,591,763 (7,289,740, 23,270,441) | 15,266,184 (10,686,329, 63,813,831) | 1,648,601 (1,154,021, 5,096,478) | 37,912,091 (26,538,464, 134,538,193) |
| Lesotho | 10,993,210 (7,695,247, 45,090,149) | 491,958 (344,370, 1,908,513) | 9,599,553 (6,719,687, 32,109,547) | 2,533,404 (1,773,383, 6,447,645) | 8,458,728 (5,921,110, 30,756,376) |
| Liberia | 55,584,475 (38,909,132, 205,739,225) | 757,599 (530,319, 2,290,724) | 893,155,801 (652,003,735, 3,892,355,999) | 777,722,950 (567,737,754, 3,028,804,446) | 1,032,534,595 (753,750,254, 8,531,115,945) |
| Libya | 22,880,595 (16,016,417, 75,425,371) | 2,288,910 (1,602,237, 6,111,804) | 141,952,066 (107,883,570, 314,301,776) | 70,824,829 (53,826,870, 176,266,792) | 81,375,815 (61,845,619, 217,874,085) |
| Lithuania | 2,770,516,747 (2,022,477,225, 15,886,973,875) | 67,103,401 (48,985,483, 434,697,485) | 13,203,362 (9,638,454, 26,385,609) | 5,996,927 (4,377,756, 13,017,798) | 28,329,446 (20,680,496, 56,149,935) |
| Luxembourg | 298,537,193 (226,888,267, 722,169,980) | 4,384,483 (3,332,207, 13,727,327) | 3,033,085 (2,123,160, 6,389,286) | 2,915,198 (2,040,639, 6,529,501) | 52,360,534 (36,652,373, 113,404,404) |
| Madagascar | 51,636,703 (37,694,793, 103,745,677) | 4,106,968 (2,998,086, 8,192,335) | 13,743,596,588 (10,032,825,510, 94,121,092,579) | 980,107,409 (715,478,408, 2,309,844,748) | 16,455,502,434 (12,012,516,777, 154,609,000,000) |
| Malawi | 59,756,767 (41,829,737, 129,293,096) | 1,447,949 (1,013,565, 2,969,906) | 3,271,831 (2,388,437, 7,748,157) | 1,497,028 (1,092,831, 4,356,281) | 1,792,122 (1,308,249, 4,789,846) |
| Malaysia | 31,482,711,570 (22,982,379,447, 251,831,365,710) | 303,505,140 (221,558,752, 791,428,383) | 17,054,633 (11,938,243, 42,783,134) | 8,304 (5,813, 35,342) | 36,893,565 (25,825,495, 86,194,974) |
| Maldives | 6,639,475 (4,846,817, 17,206,617) | 78,494 (57,301, 312,333) | 94,198,524 (71,590,878, 225,117,540) | 42,028,264 (30,680,632, 104,458,987) | 69,706,641 (52,977,047, 208,385,117) |
| Mali | 66,442,473 (46,509,731, 158,725,357) | 12,485,971 (8,740,180, 29,711,907) | 3,108,187 (2,362,222, 8,753,459) | 433,945 (329,798, 1,771,162) | 2,114,767 (1,607,223, 6,563,593) |
| Malta | 210,295,516 (158,563,745, 551,439,791) | 4,362,088 (3,315,187, 13,478,147) | 102,739,640 (74,999,937, 237,479,743) | 12,846,059 (9,377,623, 38,031,562) | 258,906,856 (189,002,005, 606,638,000) |
| Marshall Islands | 5,656,899 (4,299,244, 17,088,260) | NA | 87,969,636 (61,578,745, 398,171,288) | 126,629,668 (88,640,767, 2,129,573,047) | 115,030,425 (80,521,297, 1,147,528,509) |
| Mauritania | 377,912,925 (275,876,435, 891,513,470) | 3,420,370 (2,496,870, 9,364,165) | 5,117,521,367 (3,582,264,957, 10,135,952,290) | 2,449,351,200 (1,714,545,840, 10,802,073,528) | 4,380,127,188 (3,066,089,031, 14,637,463,107) |
| Mauritius | 365,528,579 (255,870,005, 3,985,239,720) | 35,898,850 (25,129,195, 309,966,876) | 680,130 (496,495, 1,696,151) | 101,571 (74,147, 390,248) | 529,829 (386,775, 1,441,512) |
| Mexico | 12,575,289,706 (8,802,702,794, 39,888,546,825) | 628,289,951 (439,802,966, 4,313,057,900) | 192,836,438 (146,555,693, 776,706,089) | 49,419,154 (37,558,557, 132,556,351) | 102,632,776 (78,000,909, 513,592,538) |
| Micronesia (Federated States of) | 1,311,531 (957,418, 3,528,018) | NA | 57,571,336 (43,754,215, 144,188,981) | 42,562,646 (32,347,611, 110,299,042) | 39,302,899 (29,870,203, 96,442,963) |
| Mongolia | 363,058,192 (275,924,226, 1,476,785,579) | 18,169,825 (13,809,067, 53,930,602) | 56,317,632 (42,801,400, 105,996,312) | 75,018,329 (57,013,930, 156,241,628) | 212,471,119 (161,478,050, 404,512,268) |
| Montenegro | 148,174,693 (112,612,766, 379,010,135) | 8,737,811 (6,640,737, 28,079,150) | 55,413,649 (42,114,374, 140,146,674) | 21,858,736 (16,612,639, 48,737,935) | 64,827,908 (49,269,210, 159,093,753) |
| Morocco | 366,891,048 (278,837,197, 716,677,503) | 23,083,969 (17,543,817, 49,927,295) | 1,483,198,463 (1,082,734,878, 3,160,537,822) | 168,867,043 (118,206,930, 351,997,932) | 897,247,151 (628,073,006, 1,892,938,098) |
| Mozambique | 156,454,489 (118,905,412, 388,062,307) | 14,354,196 (10,909,189, 40,083,945) | 43,136,321 (30,195,425, 99,098,227) | 24,203,830 (16,942,681, 61,311,918) | 93,502,046 (65,451,433, 234,566,460) |
| Myanmar | 2,597,719,917 (1,862,899,896, 5,519,777,852) | 48,407,261 (33,885,082, 114,304,001) | 690,861,881 (483,603,317, 1,557,526,545) | 311,792,259 (218,254,582, 792,839,868) | 673,469,806 (471,428,864, 1,695,566,710) |
| Namibia | 168,757,673 (118,130,371, 414,601,492) | 7,915,475 (5,540,833, 19,624,887) | 1,690,257,250 (1,183,180,075, 5,725,296,220) | 1,403,487,966 (1,024,546,216, 3,786,172,117) | 1,404,653,822 (983,257,675, 4,142,579,112) |
| Nepal | 1,757,501,727 (1,232,692,542, 4,241,615,034) | 81,377,780 (59,405,780, 195,681,910) | 893,070,023 (651,941,117, 7,980,371,814) | 533,918,042 (389,760,170, 1,889,344,521) | 1,418,895,836 (1,035,793,960, 14,288,020,084) |
| Netherlands | 4,616,336,434 (3,273,540,144, 13,965,641,894) | 117,937,396 (82,556,177, 311,594,445) | 19,856,815 (13,899,771, 61,404,397) | 10,556,274 (7,389,392, 25,867,625) | 17,848,277 (12,493,794, 51,029,494) |
| New Zealand | 2,880,378,424 (2,102,676,249, 24,770,994,463) | 34,494,524 (25,181,002, 613,258,044) | 23,186,743 (16,230,720, 86,105,184) | 1,518,668 (1,063,068, 4,157,455) | 31,358,581 (21,951,007, 98,384,038) |
| Nicaragua | 51,374,268 (35,961,988, 145,901,859) | 3,112,902 (2,179,032, 7,600,344) | 797,453,752 (582,141,239, 1,766,898,810) | 564,836,803 (412,330,866, 1,286,285,658) | 2,129,600,672 (1,554,608,491, 4,887,529,750) |
| Niger | 58,323,479 (40,826,435, 196,239,179) | 2,259,487 (1,581,641, 7,592,502) | 106,767,255 (81,143,113, 245,070,773) | 53,913,022 (40,973,897, 122,776,509) | 63,457,371 (48,227,602, 152,757,949) |
| Nigeria | 4,612,232,209 (3,366,929,513, 10,733,736,153) | 1,120,340,983 (817,848,917, 2,793,021,935) | 1,032,711,782 (722,898,248, 4,186,420,397) | 640,787,666 (448,551,366, 1,705,231,335) | 604,492,429 (423,144,700, 3,338,065,945) |
| North Macedonia | 244,573,476 (185,875,842, 582,243,134) | 20,435,827 (15,531,229, 61,637,902) | 9,156,856 (6,684,505, 31,718,218) | 8,932,846 (6,520,977, 26,608,819) | 12,976,057 (9,472,521, 43,315,836) |
| Norway | 2,343,325,486 (1,640,327,841, 9,495,889,522) | 65,333,610 (45,733,527, 266,171,845) | 1,799,835,376 (1,367,874,886, 3,835,062,311) | 1,186,245,486 (901,546,569, 2,244,841,278) | 3,144,421,709 (2,389,760,499, 6,811,909,469) |
| Oman | 32,819,177 (23,957,999, 107,507,770) | 1,753,418 (1,279,995, 5,864,899) | 1,130,897,345 (791,628,142, 3,438,337,748) | 702,860,357 (492,002,250, 1,662,553,929) | 1,025,026,804 (717,518,763, 3,080,209,143) |
| Pakistan | 6,776,696,706 (5,150,289,497, 14,518,347,870) | 646,194,135 (491,107,543, 1,626,534,812) | 110,880,110 (84,268,883, 242,585,279) | 6,718,673 (4,904,631, 16,661,292) | 52,001,290 (37,960,942, 106,856,847) |
| Panama | 2,901,053,959 (2,030,737,771, 8,288,080,141) | 42,269,453 (29,588,617, 106,979,321) | 253,701,456 (185,202,063, 641,792,498) | 97,055,744 (70,850,693, 218,192,088) | 184,653,355 (134,796,949, 432,557,770) |
| Papua New Guinea | 171,761,071 (128,711,985, 371,530,276) | 2,160,998 (1,577,529, 5,426,858) | 369,153,161 (258,407,212, 2,150,365,770) | 373,749,518 (261,624,663, 1,715,577,306) | 515,420,064 (360,794,045, 3,817,996,426) |
| Paraguay | 552,793,943 (403,539,579, 1,332,181,809) | 17,383,388 (12,689,874, 39,639,454) | 3,112,259,662 (2,271,949,553, 6,290,900,743) | 404,744,638 (295,463,585, 821,513,234) | 1,865,335,532 (1,361,694,938, 3,875,785,290) |
| Peru | 1,299,934,497 (909,954,148, 7,835,821,210) | 41,611,754 (29,128,228, 151,881,708) | 5,156,085,016 (3,918,624,612, 13,083,416,187) | 4,456,177,820 (3,386,695,143, 14,234,838,738) | 4,035,678,813 (3,067,115,898, 13,393,708,426) |
| Philippines | 5,457,993,641 (3,984,335,358, 11,165,949,436) | 75,653,810 (55,227,281, 177,750,168) | 817,478,803 (596,759,526, 5,237,058,167) | 285,301,688 (208,270,232, 962,092,170) | 496,836,296 (362,690,496, 2,088,774,324) |
| Poland | 14,245,757,654 (10,826,775,817, 46,102,409,568) | 597,816,005 (454,340,164, 5,390,446,217) | 206,457,455 (156,907,666, 1,051,663,479) | 154,936,859 (117,752,013, 660,944,568) | 240,150,148 (182,514,113, 1,515,214,508) |
| Portugal | 1,626,829,139 (1,187,585,272, 8,388,313,563) | 27,212,352 (19,865,017, 100,388,901) | 30,190,803 (21,133,562, 84,923,282) | 18,425,909 (12,898,136, 47,770,934) | 22,741,062 (15,918,743, 67,672,655) |
| Puerto Rico | 609,946,535 (463,055,242, 3,257,168,575) | 8,402,073 (5,881,451, 29,346,020) | 334,684,065 (230,932,005, 1,217,512,630) | 195,382,712 (134,814,071, 885,726,043) | 414,372,113 (285,916,758, 2,448,878,849) |
| Qatar | 73,752,057 (51,626,440, 208,360,656) | 2,394,283 (1,675,998, 7,993,785) | 3,989,615,681 (2,912,419,447, 9,982,826,733) | 2,284,144,741 (1,667,425,661, 7,183,452,808) | 2,513,683,347 (1,834,988,843, 6,628,321,234) |
| Republic of Moldova | 999,215,569 (689,458,743, 4,826,507,757) | 54,776,679 (37,795,908, 274,390,235) | 21,522,505,931 (15,065,754,152, 89,617,267,335) | 11,606,918,685 (8,124,843,080, 38,344,465,278) | 26,224,120,399 (18,356,884,279, 87,831,374,250) |
| Romania | 9,224,327,397 (6,733,759,000, 26,728,488,264) | 436,883,628 (318,925,049, 2,933,887,489) | 143,432,355 (100,402,649, 448,105,396) | 106,942,143 (74,859,500, 314,578,799) | 345,260,212 (241,682,148, 1,237,304,208) |
| Russian Federation | 61,612,010,571 (43,128,407,400, 226,843,921,109) | 2,258,465,556 (1,580,925,889, 11,050,814,246) | 3,410,397 (2,591,902, 8,565,315) | 4,759,224 (3,617,010, 12,470,549) | 6,938,166 (5,273,006, 18,204,330) |
| Rwanda | 629,999,834 (440,999,884, 2,104,646,056) | 34,365,124 (24,055,587, 104,657,653) | 6,037,487 (4,226,241, 15,676,177) | 2,748,854 (1,924,198, 7,919,043) | 8,719,994 (6,103,996, 21,934,619) |
| Saint Lucia | 15,430,958 (11,727,528, 40,225,799) | 323,170 (245,610, 985,604) | 5,327,965 (4,049,253, 12,784,343) | 585,906 (445,289, 1,841,518) | 4,651,378 (3,395,506, 11,626,181) |
| Saint Vincent and the Grenadines | 17,848,888 (12,494,221, 46,802,534) | 342,552 (239,787, 1,272,696) | 901,503 (631,052, 2,627,007) | 160,996 (112,697, 674,313) | 5,969,708 (4,178,795, 16,824,322) |
| Samoa | 11,494,059 (8,568,079, 29,014,418) | 928,809 (678,031, 2,762,377) | 1,120,958,114 (818,299,424, 3,121,205,873) | 376,474,930 (263,532,451, 821,208,593) | 1,763,130,612 (1,234,191,428, 4,391,901,167) |
| Sao Tome and Principe | 7,032,207 (4,922,545, 20,125,683) | NA | 40,722,754 (29,727,610, 194,323,546) | 6,801,752 (4,965,279, 17,813,550) | 111,343,269 (81,280,586, 319,317,715) |
| Saudi Arabia | 3,340,105,014 (2,371,702,253, 8,573,361,833) | 79,541,358 (55,678,951, 239,046,200) | 522,828,103 (381,664,515, 1,483,777,217) | 381,885,044 (278,776,082, 921,901,241) | 322,159,716 (235,176,593, 946,724,804) |
| Senegal | 162,696,642 (118,768,549, 540,773,930) | 3,828,867 (2,795,073, 9,319,119) | 21,662,960 (16,463,850, 58,317,655) | 8,845,614 (6,722,667, 26,128,472) | 18,270,278 (13,885,411, 50,557,532) |
| Serbia | 1,278,514,166 (933,315,341, 3,493,258,069) | 51,641,304 (37,698,152, 140,854,807) | 7,620,090 (5,334,063, 26,729,148) | 792,034 (554,424, 2,380,329) | 15,824,796 (11,552,101, 35,739,959) |
| Seychelles | 49,489,427 (37,611,965, 138,048,650) | 710,574 (540,037, 3,044,990) | 656,281,312 (459,396,918, 1,734,499,097) | 114,571,623 (80,200,136, 248,539,364) | 591,842,635 (414,289,844, 1,792,401,534) |
| Sierra Leone | 24,714,714 (17,789,377, 66,027,236) | 477,793 (348,789, 1,177,800) | 549,192,109 (417,386,003, 1,188,176,738) | 419,313,609 (318,678,343, 931,473,675) | 302,551,658 (229,939,260, 729,494,177) |
| Singapore | 1,397,009,461 (977,906,623, 3,985,984,610) | 34,313,892 (24,019,724, 210,544,614) | 129,506,423 (98,424,881, 515,381,904) | 173,210,538 (131,640,009, 478,929,559) | 197,113,244 (149,806,066, 980,432,473) |
| Slovakia | 1,329,134,148 (1,010,141,953, 2,977,884,276) | 58,076,773 (44,138,347, 128,739,686) | 2,827,309 (2,148,755, 6,757,096) | 241,381 (183,449, 734,449) | 1,771,874 (1,346,624, 4,265,804) |
| Slovenia | 506,066,482 (384,610,527, 1,990,685,929) | 6,236,278 (4,739,571, 15,941,993) | 128,133,821 (89,693,675, 339,890,972) | 21,674,518 (15,172,163, 48,119,918) | 253,132,974 (177,193,082, 630,776,487) |
| Solomon Islands | 4,920,135 (3,739,303, 12,033,812) | 79,572 (60,475, 276,463) | 2,502,627,593 (1,751,839,315, 5,562,084,652) | 1,062,007,085 (743,404,960, 3,166,139,604) | 1,879,272,683 (1,315,490,878, 5,299,216,157) |
| Somalia | 461,104,483 (322,773,138, 1,171,016,618) | 58,163,170 (40,714,219, 152,229,240) | 8,083,491,459 (5,658,444,022, 96,299,960,964) | 140,886,354 (98,620,447, 442,470,450) | 1,277,055,184 (893,938,629, 11,479,600,391) |
| South Africa | 13,955,726,445 (10,606,352,098, 41,256,042,476) | 536,952,127 (408,083,616, 1,882,470,202) | 52,851,465 (38,581,570, 94,776,751) | 8,673,274 (6,331,490, 18,546,045) | 29,823,005 (21,770,793, 59,831,132) |
| Spain | 5,643,454,143 (3,950,417,900, 15,058,056,649) | 199,546,782 (139,682,747, 1,030,616,236) | 13,389,597 (9,238,822, 35,216,270) | 5,613,826 (3,873,540, 14,175,730) | 12,820,774 (8,846,334, 33,281,997) |
| Sri Lanka | 9,540,660,653 (6,678,462,458, 108,391,955,380) | 39,227,656 (27,459,359, 169,923,575) | 556,653,500 (423,056,660, 3,050,796,652) | 671,992,574 (839,679,640, 1,562,014,861) | 660,920,233 (502,299,377, 2,298,558,643) |
| Sudan | 94,656,087 (69,098,944, 180,650,312) | 3,308,343 (2,415,091, 7,496,384) | 977,607,366 (684,325,156, 5,722,087,889) | 471,440,585 (330,008,409, 2,845,121,331) | 664,533,752 (465,173,627, 4,243,137,159) |
| Suriname | 33,602,641 (23,185,822, 87,519,698) | 1,778,445 (1,227,127, 4,845,701) | 131,735,352 (100,118,868, 456,555,699) | 11,492,080 (8,733,981, 38,976,561) | 78,758,822 (59,856,705, 363,353,980) |
| Sweden | 1,916,794,292 (1,785,728,945, 7,130,808,596) | 27,227,984 (20,693,268, 219,438,440) | 9,888,917,039 (7,218,909,438, 25,610,503,982) | 1,028,848,639 (751,059,507, 3,483,673,424) | 6,302,746,964 (4,601,005,284, 27,550,172,668) |
| Switzerland | 2,128,648,146 (1,490,505,695, 12,871,139,188) | 15,066,442 (10,998,503, 60,792,809) | 25,345,927 (18,502,527, 94,305,616) | 1,157,154 (844,722, 3,340,200) | 11,774,676 (8,595,513, 41,975,680) |
| Tajikistan | 226,153,997 (171,877,038, 872,084,129) | 4,167,742 (3,167,484, 13,197,889) | 20,277,911 (14,802,875, 50,598,356) | 2,315,946 (1,690,640, 6,738,308) | 43,674,379 (31,882,297, 89,923,319) |
| Thailand | 17,355,212,603 (12,669,305,200, 57,143,661,610) | 134,699,961 (98,330,971, 499,311,536) | 3,288,166 (2,499,006, 8,598,904) | 2,128,407 (1,617,589, 6,270,731) | 4,809,550 (3,655,258, 12,694,886) |
| Timor-Leste | 39,643,842 (28,940,004, 144,900,787) | 1,366,085 (997,242, 5,279,291) | 32,627,023 (22,838,916, 93,067,235) | 25,943,762 (18,160,633, 65,311,271) | 34,373,425 (24,061,397, 133,954,533) |
| Togo | 67,459,391 (49,245,355, 150,488,822) | 1,191,155 (869,543, 3,228,838) | 71,847,949 (50,293,564, 138,013,230) | 53,335,877 (37,335,114, 108,427,980) | 129,271,641 (90,490,149, 243,396,493) |
| Tonga | 10,226,122 (7,771,853, 27,564,553) | NA | 24,132,586,991 (17,616,788,504, 65,449,578,825) | 12,535,687,274 (9,151,051,710, 32,763,999,015) | 15,931,094,686 (11,629,699,121, 45,871,110,313) |
| Trinidad and Tobago | 95,177,649 (66,624,354, 299,075,063) | 2,233,440 (1,563,408, 6,742,025) | 82,032,220 (62,344,487, 220,363,649) | 167,952,725 (127,644,071, 397,360,900) | 496,095,212 (377,032,361, 1,483,252,185) |
| Tunisia | 270,385,602 (189,269,921, 522,183,823) | 15,930,135 (11,151,094, 32,346,120) | 6,275,806,177 (4,769,612,694, 31,969,035,579) | 1,319,492,539 (963,229,554, 5,685,203,966) | 4,296,499,978 (3,265,339,983, 20,012,242,023) |
| Turkey | 53,450,792,232 (39,019,078,330, 146,417,274,491) | 851,423,281 (621,538,995, 2,332,586,338) | 163,442,819 (119,313,258, 605,908,987) | 143,100,582 (104,463,425, 519,165,625) | 149,331,360 (109,011,893, 528,028,052) |
| Uganda | 787,856,334 (598,770,814, 2,233,880,118) | 41,776,176 (31,749,894, 132,903,383) | 6,144,445,200 (4,485,444,996, 16,366,580,530) | 4,123,478,732 (3,010,139,474, 13,233,521,516) | 5,853,186,097 (4,272,825,851, 19,777,747,514) |
| Ukraine | 13,220,542,124 (10,008,027,237, 64,994,963,515) | 1,328,743,430 (1,009,845,006, 7,328,481,947) | 588,242,947 (447,064,640, 1,310,598,380) | 482,239,208 (366,501,798, 970,310,592) | 1,402,304,936 (1,065,751,751, 2,935,268,064) |
| United Arab Emirates | 469,930,477 (343,049,248, 1,701,952,568) | 14,055,716 (10,260,673, 48,849,905) | 69,146,413,995 (48,402,489,797, 147,224,000,000) | 46,553,877,944 (32,587,714,561, 118,509,000,000) | 63,910,085,163 (44,737,059,614, 167,490,000,000) |
| United Kingdom | 17,226,660,305 (12,575,462,022, 59,050,943,679) | 1,105,550,276 (807,051,701, 9,673,094,119) | 468,436,279 (327,905,395, 1,657,743,477) | 268,798,854 (188,159,198, 591,631,038) | 487,351,402 (341,145,981, 1,305,794,040) |
| United Republic of Tanzania | 2,603,430,264 (1,978,607,000, 5,508,008,030) | 130,643,174 (99,288,812, 291,830,994) | 396,069,924 (301,013,142, 2,228,558,693) | 95,691,366 (72,725,438, 302,054,428) | 135,611,662 (103,064,863, 440,561,333) |
| United States of America | 183,117,222,486 (128,182,055,740, 443,025,752,810) | 3,506,845,384 (2,454,791,768, 9,802,752,810) | 1,649,691 (1,253,765, 3,986,262) | 176,993 (134,515, 603,582) | 1,169,295 (853,586, 3,014,361) |
| Uruguay | 1,260,990,891 (882,693,623, 3,809,204,904) | 36,404,356 (25,483,049, 254,036,349) | 11,899,626,756 (9,043,716,334, 22,336,444,293) | 1,659,017,218 (1,260,853,086, 3,343,315,516) | 17,753,364,708 (13,492,557,178, 34,772,935,794) |
| Uzbekistan | 668,252,227 (507,871,692, 3,206,844,621) | 40,879,275 (31,068,249, 235,670,167) | 23,882,920 (16,718,044, 57,079,086) | 24,818,181 (17,372,726, 81,864,586) | 72,410,169 (50,687,118, 189,645,925) |
| Vanuatu | 3,083,502 (2,305,757, 8,513,038) | 87,523 (63,891, 908,833) | 59,763,915 (45,420,575, 396,364,512) | 28,528,922 (21,681,981, 148,422,671) | 189,010,630 (143,648,079, 1,656,286,903) |
| Venezuela (Bolivarian Republic of) | 4,887,033,160 (3,420,923,212, 17,090,598,578) | 414,360,141 (290,052,099, 1,867,776,950) | 694,475,669 (527,801,509, 1,819,377,638) | 61,969,703 (47,096,974, 158,629,297) | 1,402,304,936 (1,065,751,751, 2,935,268,064) |
| VietNam | 32,254,121,318 (24,513,132,201, 62,292,066,952) | 942,112,636 (716,005,603, 1,839,371,349) | 53,140,595 (40,386,852, 218,026,041) | 20,814,522 (15,819,037, 51,901,442) | 63,910,085,163 (44,737,059,614, 167,490,000,000) |
| Yemen | 6,476,201,324 (4,533,340,927, 18,848,463,786) | 407,929,038 (285,550,327, 1,480,756,038) | 147,517,022 (103,261,915, 284,737,362) | 83,754,463 (58,628,124, 167,289,690) | 487,351,402 (341,145,981, 1,305,794,040) |
| Zambia | 126,705,342 (88,693,739, 343,276,835) | 5,594,073 (3,915,851, 14,687,238) | 111,457,901 (84,708,005, 288,065,514) | 34,360,137 (25,082,900, 84,506,716) | 135,611,662 (103,064,863, 440,561,333) |
| Zimbabwe | 291,431,217 (221,487,725, 2,295,929,391) | 14,127,750 (10,737,090, 94,855,305) | 2,722,812 (1,987,652, 7,444,212) | 1,943,088 (1,418,454, 5,715,083) | 1,169,295 (853,586, 3,014,361) |

Note: GDP, Gross Domestic Product

# Table S5 Economic burden of four subtypes of cancer and their percentage of GDP in various countries in 2021

| Country | Non-hodgkin lymphoma | | multiple myeloma | | Leukemia | | Hodgkin Lymphoma | |
| --- | --- | --- | --- | --- | --- | --- | --- | --- |
|  | Economic Burden ($) | Proportion of Economic Burden to National GDP | Economic Burden ($) | Proportion of Economic Burden to National GDP | Economic Burden ($) | Proportion of Economic Burden to National GDP | Economic Burden ($) | Proportion of Economic Burden to National GDP |
| Afghanistan | 27,026,228 (17,500,007, 45,801,963) | 2.5E-05 (1.6E-05, 4.2E-05) | 2,573,585 (1,025,676, 5,833,711) | 2.3E-06 (9.3E-07, 5.3E-06) | 129,083,256 (75,374,192, 186,921,319) | 1.2E-04 (6.8E-05, 1.7E-04) | 13,758,632 (8,874,676, 21,879,235) | 1.2E-05 (8.1E-06, 2.0E-05) |
| Albania | 23,287,839 (17,666,476, 30,388,242) | 2.6E-05 (2.0E-05, 3.4E-05) | 10,845,493 (7,088,711, 15,032,403) | 1.2E-05 (8.0E-06, 1.7E-05) | 68,337,077 (47,504,551, 99,411,140) | 7.7E-05 (5.4E-05, 1.1E-04) | 8,163,663 (5,404,154, 13,003,147) | 9.3E-06 (6.1E-06, 1.5E-05) |
| Algeria | 560,822,056 (445,083,937, 710,465,127) | 6.9E-05 (5.5E-05, 8.8E-05) | 172,525,336 (111,604,166, 238,218,229) | 2.1E-05 (1.4E-05, 2.9E-05) | 620,782,521 (456,139,305, 798,928,227) | 7.7E-05 (5.6E-05, 9.9E-05) | 189,967,168 (123,020,762, 273,232,753) | 2.3E-05 (1.5E-05, 3.4E-05) |
| Andorra | 38,732,951 (26,198,657, 52,776,647) | 1.5E-02 (1.0E-02, 2.1E-02) | 10,571,996 (6,937,737, 14,996,179) | 4.1E-03 (2.7E-03, 5.8E-03) | 31,715,072 (20,641,124, 44,543,255) | 1.2E-02 (8.0E-03, 1.7E-02) | 1,845,684 (1,064,308, 2,800,499) | 7.2E-04 (4.1E-04, 1.1E-03) |
| Angola | 151,810,253 (100,373,293, 216,430,784) | 1.0E-04 (6.8E-05, 1.5E-04) | 15,332,420 (9,293,339, 22,608,866) | 1.0E-05 (6.3E-06, 1.5E-05) | 178,159,976 (112,625,845, 253,164,916) | 1.2E-04 (7.6E-05, 1.7E-04) | 23,759,473 (14,888,521, 37,218,616) | 1.6E-05 (1.0E-05, 2.5E-05) |
| Antigua and Barbuda | 7,666,935 (7,239,772, 8,200,094) | 2.4E-03 (2.3E-03, 2.6E-03) | 5,652,149 (5,184,450, 6,176,809) | 1.8E-03 (1.6E-03, 1.9E-03) | 9,143,733 (8,593,851, 9,708,720) | 2.9E-03 (2.7E-03, 3.0E-03) | 242,197 (218,002, 271,588) | 7.5E-05 (6.8E-05, 8.5E-05) |
| Argentina | 2,709,468,025 (2,492,861,109, 2,947,271,697) | 3.9E-03 (3.6E-03, 4.3E-03) | 1,029,617,359 (954,268,131, 1,105,275,502) | 1.5E-03 (1.4E-03, 1.6E-03) | 3,658,897,225 (3,433,196,996, 3,942,800,405) | 5.3E-03 (5.0E-03, 5.7E-03) | 331,021,416 (277,835,894, 392,614,868) | 4.8E-04 (4.0E-04, 5.7E-04) |
| Armenia | 51,167,803 (42,094,923, 60,513,515) | 1.3E-05 (1.0E-05, 1.5E-05) | 26,374,359 (21,355,205, 31,376,259) | 6.6E-06 (5.3E-06, 7.8E-06) | 120,414,887 (106,820,856, 136,030,851) | 3.0E-05 (2.7E-05, 3.4E-05) | 8,181,266 (6,654,763, 9,754,656) | 2.0E-06 (1.7E-06, 2.4E-06) |
| Australia | 8,004,272,205 (7,241,379,404, 8,651,334,555) | 3.5E-03 (3.2E-03, 3.8E-03) | 4,974,665,589 (4,356,792,440, 5,611,987,977) | 2.2E-03 (1.9E-03, 2.4E-03) | 8,711,997,520 (7,994,221,577, 9,384,772,188) | 3.8E-03 (3.5E-03, 4.1E-03) | 499,334,402 (408,122,405, 616,010,908) | 2.2E-04 (1.8E-04, 2.7E-04) |
| Austria | 3,304,594,069 (3,002,418,590, 3,552,784,574) | 9.1E-03 (8.3E-03, 9.8E-03) | 1,884,134,450 (1,678,839,739, 2,064,953,975) | 5.2E-03 (4.6E-03, 5.7E-03) | 3,883,623,215 (3,527,805,006, 4,198,598,581) | 1.1E-02 (9.7E-03, 1.2E-02) | 290,867,791 (245,751,581, 341,259,733) | 8.0E-04 (6.8E-04, 9.4E-04) |
| Azerbaijan | 138,174,626 (100,204,531, 188,900,596) | 4.5E-03 (3.3E-03, 6.1E-03) | 28,886,427 (18,190,205, 43,062,126) | 9.4E-04 (5.9E-04, 1.4E-03) | 554,943,418 (388,501,051, 784,777,122) | 1.8E-02 (1.3E-02, 2.6E-02) | 80,131,517 (48,063,346, 125,019,373) | 2.6E-03 (1.6E-03, 4.1E-03) |
| Bahamas | 52,482,923 (41,810,629, 66,046,864) | 4.7E-03 (3.7E-03, 5.9E-03) | 43,642,918 (34,841,237, 54,564,335) | 3.9E-03 (3.1E-03, 4.9E-03) | 44,656,861 (35,574,507, 55,706,478) | 4.0E-03 (3.2E-03, 5.0E-03) | 4,627,472 (3,638,657, 5,897,122) | 4.1E-04 (3.2E-04, 5.2E-04) |
| Bahrain | 161,442,124 (128,905,003, 199,446,994) | 1.3E-02 (1.0E-02, 1.6E-02) | 66,311,795 (37,550,292, 98,493,402) | 5.2E-03 (3.0E-03, 7.7E-03) | 261,720,216 (187,904,214, 354,215,993) | 2.1E-02 (1.5E-02, 2.8E-02) | 39,559,985 (24,441,430, 60,412,716) | 3.1E-03 (1.9E-03, 4.8E-03) |
| Bangladesh | 728,971,265 (531,240,219, 1,058,872,680) | 2.6E-05 (1.9E-05, 3.7E-05) | 206,976,615 (121,172,435, 354,749,873) | 7.3E-06 (4.3E-06, 1.3E-05) | 1,395,505,108 (984,468,235, 1,878,814,446) | 4.9E-05 (3.5E-05, 6.6E-05) | 240,752,803 (132,684,998, 502,073,607) | 8.5E-06 (4.7E-06, 1.8E-05) |
| Barbados | 35,624,313 (28,115,945, 44,712,745) | 4.5E-03 (3.6E-03, 5.7E-03) | 11,913,614 (8,929,447, 15,087,414) | 1.5E-03 (1.1E-03, 1.9E-03) | 28,572,622 (22,220,201, 35,507,907) | 3.6E-03 (2.8E-03, 4.5E-03) | 1,929,976 (1,484,738, 2,453,296) | 2.4E-04 (1.9E-04, 3.1E-04) |
| Belarus | 774,109,113 (629,933,209, 950,057,216) | 6.1E-03 (5.0E-03, 7.5E-03) | 412,573,865 (327,932,449, 508,368,009) | 3.3E-03 (2.6E-03, 4.0E-03) | 1,111,111,344 (911,603,323, 1,354,230,533) | 8.8E-03 (7.2E-03, 1.1E-02) | 144,697,598 (111,398,278, 192,184,112) | 1.1E-03 (8.8E-04, 1.5E-03) |
| Belgium | 4,278,669,272 (3,843,808,271, 4,733,490,902) | 9.5E-03 (8.5E-03, 1.0E-02) | 2,899,298,811 (2,562,239,367, 3,181,841,022) | 6.4E-03 (5.7E-03, 7.0E-03) | 5,453,805,325 (4,885,504,578, 5,965,933,274) | 1.2E-02 (1.1E-02, 1.3E-02) | 355,255,326 (288,944,882, 432,553,952) | 7.9E-04 (6.4E-04, 9.6E-04) |
| Belize | 2,859,854 (2,545,741, 3,233,705) | 6.2E-04 (5.5E-04, 7.0E-04) | 796,232 (692,875, 908,743) | 1.7E-04 (1.5E-04, 2.0E-04) | 5,324,956 (4,776,845, 5,957,804) | 1.2E-03 (1.0E-03, 1.3E-03) | 818,733 (694,998, 956,983) | 1.8E-04 (1.5E-04, 2.1E-04) |
| Benin | 23,791,158 (16,137,887, 30,389,244) | 2.6E-06 (1.7E-06, 3.3E-06) | 284,137 (84,198, 469,523) | 3.0E-08 (9.0E-09, 5.0E-08) | 22,153,338 (10,323,784, 32,651,024) | 2.4E-06 (1.1E-06, 3.5E-06) | 1,164,801 (726,701, 1,915,010) | 1.2E-07 (7.8E-08, 2.1E-07) |
| Bhutan | 8,182,000 (5,350,816, 13,973,075) | 4.8E-05 (3.2E-05, 8.2E-05) | 2,621,797 (1,311,507, 4,790,001) | 1.5E-05 (7.7E-06, 2.8E-05) | 13,198,290 (8,396,470, 20,381,437) | 7.8E-05 (5.0E-05, 1.2E-04) | 2,340,094 (1,148,640, 5,266,015) | 1.4E-05 (6.8E-06, 3.1E-05) |
| Bolivia (Plurinational State of) | 179,952,390 (131,999,636, 243,163,214) | 3.8E-03 (2.8E-03, 5.1E-03) | 39,900,919 (27,206,732, 59,661,539) | 8.4E-04 (5.7E-04, 1.3E-03) | 302,905,361 (210,801,355, 407,167,817) | 6.4E-03 (4.4E-03, 8.5E-03) | 21,133,855 (11,761,128, 33,827,555) | 4.4E-04 (2.5E-04, 7.1E-04) |
| Bosnia and Herzegovina | 105,680,167 (76,278,266, 133,726,972) | 3.1E-03 (2.2E-03, 3.9E-03) | 54,971,222 (36,906,625, 73,142,202) | 1.6E-03 (1.1E-03, 2.1E-03) | 163,747,882 (120,504,628, 207,149,360) | 4.8E-03 (3.5E-03, 6.0E-03) | 16,501,530 (10,928,000, 26,653,687) | 4.8E-04 (3.2E-04, 7.8E-04) |
| Botswana | 56,395,522 (41,020,994, 78,354,116) | 3.0E-04 (2.2E-04, 4.2E-04) | 19,741,939 (13,235,367, 29,153,353) | 1.1E-04 (7.1E-05, 1.6E-04) | 51,214,556 (34,081,888, 73,494,493) | 2.7E-04 (1.8E-04, 3.9E-04) | 7,772,159 (4,301,576, 13,279,095) | 4.1E-05 (2.3E-05, 7.1E-05) |
| Brazil | 5,328,563,295 (5,077,889,105, 5,526,737,046) | 4.4E-03 (4.2E-03, 4.5E-03) | 2,917,504,902 (2,757,252,130, 3,035,888,855) | 2.4E-03 (2.3E-03, 2.5E-03) | 8,108,534,910 (7,761,509,649, 8,427,413,890) | 6.6E-03 (6.4E-03, 6.9E-03) | 667,508,882 (619,832,848, 714,505,981) | 5.5E-04 (5.1E-04, 5.9E-04) |
| Brunei Darussalam | 160,718,758 (135,802,852, 185,890,991) | 8.5E-03 (7.1E-03, 9.8E-03) | 42,853,401 (30,737,378, 56,562,672) | 2.3E-03 (1.6E-03, 3.0E-03) | 158,198,586 (120,841,457, 189,215,498) | 8.3E-03 (6.4E-03, 1.0E-02) | 13,686,788 (8,982,089, 19,078,665) | 7.2E-04 (4.7E-04, 1.0E-03) |
| Bulgaria | 687,946,322 (595,504,705, 789,272,264) | 6.6E-03 (5.7E-03, 7.5E-03) | 291,178,015 (224,857,087, 375,687,698) | 2.8E-03 (2.1E-03, 3.6E-03) | 1,091,526,270 (886,779,558, 1,320,114,112) | 1.0E-02 (8.5E-03, 1.3E-02) | 126,821,793 (98,179,557, 162,540,665) | 1.2E-03 (9.4E-04, 1.6E-03) |
| Burkina Faso | 24,641,757 (17,464,346, 31,781,630) | 2.6E-06 (1.8E-06, 3.3E-06) | 292,816 (96,287, 498,474) | 3.0E-08 (1.0E-08, 5.2E-08) | 22,233,510 (10,587,136, 31,796,864) | 2.3E-06 (1.1E-06, 3.3E-06) | 1,242,719 (811,725, 1,964,480) | 1.3E-07 (8.4E-08, 2.0E-07) |
| Burundi | 4,983,272 (3,361,634, 7,565,308) | 2.7E-06 (1.8E-06, 4.1E-06) | 386,836 (176,158, 758,855) | 2.1E-07 (9.6E-08, 4.1E-07) | 4,199,266 (2,167,721, 6,737,824) | 2.3E-06 (1.2E-06, 3.7E-06) | 1,649,143 (842,619, 2,594,881) | 9.0E-07 (4.6E-07, 1.4E-06) |
| Cabo Verde | 2,771,954 (1,456,709, 3,654,244) | 1.6E-05 (8.3E-06, 2.1E-05) | 76,608 (27,721, 128,400) | 4.4E-07 (1.6E-07, 7.3E-07) | 3,302,362 (2,425,879, 4,240,245) | 1.9E-05 (1.4E-05, 2.4E-05) | 9,357 (4,920, 13,753) | 5.3E-08 (2.8E-08, 7.8E-08) |
| Cambodia | 55,391,025 (40,308,104, 81,836,520) | 4.3E-07 (3.1E-07, 6.3E-07) | 5,336,827 (3,017,207, 10,778,683) | 4.1E-08 (2.3E-08, 8.3E-08) | 132,642,500 (93,952,194, 178,455,833) | 1.0E-06 (7.2E-07, 1.4E-06) | 5,558,724 (2,823,369, 12,261,955) | 4.3E-08 (2.2E-08, 9.4E-08) |
| Cameroon | 112,006,248 (79,866,240, 148,121,300) | 4.8E-06 (3.4E-06, 6.4E-06) | 1,646,626 (481,087, 2,709,574) | 7.1E-08 (2.1E-08, 1.2E-07) | 75,518,131 (35,223,074, 109,196,050) | 3.2E-06 (1.5E-06, 4.7E-06) | 4,786,568 (2,927,780, 7,735,957) | 2.1E-07 (1.3E-07, 3.3E-07) |
| Canada | 13,204,666,981 (11,828,103,454, 14,558,227,687) | 5.9E-03 (5.3E-03, 6.5E-03) | 7,044,967,079 (6,394,376,353, 7,681,221,671) | 3.1E-03 (2.9E-03, 3.4E-03) | 12,314,545,063 (11,304,498,495, 13,229,065,675) | 5.5E-03 (5.0E-03, 5.9E-03) | 719,154,715 (584,629,594, 875,440,107) | 3.2E-04 (2.6E-04, 3.9E-04) |
| Chad | 11,897,463 (7,857,136, 16,569,357) | 1.4E-06 (9.3E-07, 2.0E-06) | 107,912 (36,396, 180,813) | 1.3E-08 (4.3E-09, 2.1E-08) | 12,408,375 (6,557,183, 18,987,859) | 1.5E-06 (7.7E-07, 2.2E-06) | 768,449 (498,873, 1,284,295) | 9.1E-08 (5.9E-08, 1.5E-07) |
| Chile | 1,688,876,420 (1,541,630,149, 1,831,069,423) | 8.5E-06 (7.7E-06, 9.2E-06) | 1,140,788,977 (1,053,633,021, 1,240,937,023) | 5.7E-06 (5.3E-06, 6.2E-06) | 2,070,850,380 (1,936,489,164, 2,207,466,714) | 1.0E-05 (9.7E-06, 1.1E-05) | 189,036,431 (158,740,491, 226,514,038) | 9.5E-07 (8.0E-07, 1.1E-06) |
| China | 43,886,092,927 (34,269,885,643, 53,325,949,687) | 4.4E-04 (3.5E-04, 5.4E-04) | 11,627,330,948 (7,342,499,486, 15,382,499,545) | 1.2E-04 (7.4E-05, 1.6E-04) | 75,780,064,277 (55,423,490,248, 94,041,218,618) | 7.6E-04 (5.6E-04, 9.5E-04) | 2,549,481,815 (1,591,032,938, 3,457,121,228) | 2.6E-05 (1.6E-05, 3.5E-05) |
| Colombia | 1,384,780,940 (1,168,577,342, 1,658,053,032) | 1.5E-06 (1.3E-06, 1.8E-06) | 560,058,702 (463,647,336, 679,731,555) | 6.2E-07 (5.1E-07, 7.5E-07) | 2,253,879,271 (1,892,356,132, 2,657,693,265) | 2.5E-06 (2.1E-06, 2.9E-06) | 175,846,119 (143,241,829, 218,326,361) | 1.9E-07 (1.6E-07, 2.4E-07) |
| Comoros | 2,579,627 (1,776,410, 3,549,117) | 6.1E-06 (4.2E-06, 8.4E-06) | 398,239 (210,695, 591,810) | 9.4E-07 (5.0E-07, 1.4E-06) | 1,722,846 (1,111,830, 2,516,786) | 4.1E-06 (2.6E-06, 5.9E-06) | 595,113 (343,122, 898,800) | 1.4E-06 (8.1E-07, 2.1E-06) |
| Congo | 27,347,965 (20,212,646, 37,508,731) | 6.2E-06 (4.5E-06, 8.4E-06) | 3,180,756 (1,960,697, 4,806,757) | 7.2E-07 (4.4E-07, 1.1E-06) | 21,730,252 (14,052,063, 30,342,235) | 4.9E-06 (3.2E-06, 6.8E-06) | 3,432,309 (2,240,381, 5,221,499) | 7.7E-07 (5.0E-07, 1.2E-06) |
| Costa Rica | 256,377,611 (228,212,511, 287,023,207) | 6.9E-06 (6.1E-06, 7.7E-06) | 122,066,041 (106,941,669, 137,416,357) | 3.3E-06 (2.9E-06, 3.7E-06) | 365,859,335 (329,028,871, 400,010,273) | 9.8E-06 (8.8E-06, 1.1E-05) | 36,547,337 (30,073,090, 44,248,766) | 9.8E-07 (8.1E-07, 1.2E-06) |
| Coted' Ivoire | 168,132,321 (123,510,834, 230,142,341) | 4.5E-06 (3.3E-06, 6.1E-06) | 21,551,661 (13,472,006, 31,208,177) | 5.7E-07 (3.6E-07, 8.3E-07) | 59,544,526 (32,471,820, 93,981,256) | 1.6E-06 (8.6E-07, 2.5E-06) | 25,974,730 (14,645,076, 39,122,949) | 6.9E-07 (3.9E-07, 1.0E-06) |
| Croatia | 1,079,079,758 (922,821,254, 1,250,390,086) | 2.0E-02 (1.7E-02, 2.3E-02) | 657,282,739 (561,873,241, 778,945,340) | 1.2E-02 (1.0E-02, 1.4E-02) | 1,337,925,226 (1,125,550,384, 1,582,966,769) | 2.5E-02 (2.1E-02, 2.9E-02) | 70,922,260 (58,597,658, 85,088,309) | 1.3E-03 (1.1E-03, 1.6E-03) |
| Cyprus | 261,239,172 (213,015,328, 314,447,461) | 1.1E-02 (8.9E-03, 1.3E-02) | 144,158,676 (92,495,447, 193,544,013) | 6.0E-03 (3.9E-03, 8.1E-03) | 316,486,479 (220,073,952, 395,855,874) | 1.3E-02 (9.2E-03, 1.7E-02) | 25,032,882 (16,617,284, 38,916,738) | 1.0E-03 (7.0E-04, 1.6E-03) |
| Czechia | 3,209,063,105 (2,652,018,906, 3,849,562,777) | 6.2E-04 (5.1E-04, 7.4E-04) | 1,974,382,602 (1,661,266,840, 2,278,246,685) | 3.8E-04 (3.2E-04, 4.4E-04) | 4,396,183,287 (3,762,380,452, 5,067,541,420) | 8.5E-04 (7.2E-04, 9.8E-04) | 322,181,050 (249,074,928, 407,772,536) | 6.2E-05 (4.8E-05, 7.9E-05) |
| Denmark | 2,616,988,373 (2,371,210,367, 2,862,427,409) | 1.2E-03 (1.1E-03, 1.3E-03) | 1,860,045,492 (1,684,832,053, 2,054,059,020) | 8.4E-04 (7.6E-04, 9.3E-04) | 3,612,270,256 (3,302,928,287, 3,909,834,201) | 1.6E-03 (1.5E-03, 1.8E-03) | 139,996,660 (114,373,830, 169,000,179) | 6.3E-05 (5.2E-05, 7.6E-05) |
| Djibouti | 8,434,139 (5,521,403, 12,540,139) | 1.5E-05 (1.0E-05, 2.3E-05) | 1,541,916 (846,828, 2,406,371) | 2.8E-06 (1.5E-06, 4.4E-06) | 5,852,612 (3,219,723, 9,978,829) | 1.1E-05 (5.8E-06, 1.8E-05) | 2,014,625 (1,107,191, 3,300,745) | 3.6E-06 (2.0E-06, 6.0E-06) |
| Dominica | 3,428,035 (2,693,385, 4,552,723) | 3.1E-03 (2.4E-03, 4.1E-03) | 1,050,136 (727,281, 1,455,267) | 9.5E-04 (6.5E-04, 1.3E-03) | 3,067,026 (2,269,736, 3,875,520) | 2.8E-03 (2.0E-03, 3.5E-03) | 234,086 (146,164, 360,911) | 2.1E-04 (1.3E-04, 3.2E-04) |
| Dominican Republic | 247,943,110 (188,383,808, 316,079,345) | 9.0E-05 (6.8E-05, 1.1E-04) | 127,448,660 (89,069,877, 176,603,576) | 4.6E-05 (3.2E-05, 6.4E-05) | 467,311,049 (350,374,426, 605,415,243) | 1.7E-04 (1.3E-04, 2.2E-04) | 7,696,863 (3,886,884, 17,219,462) | 2.8E-06 (1.4E-06, 6.3E-06) |
| Ecuador | 372,446,934 (295,094,368, 467,215,504) | 3.5E-03 (2.8E-03, 4.4E-03) | 102,291,414 (80,686,657, 129,718,308) | 9.5E-04 (7.5E-04, 1.2E-03) | 624,356,759 (517,049,519, 742,425,655) | 5.8E-03 (4.8E-03, 6.9E-03) | 45,624,964 (35,188,738, 58,581,456) | 4.3E-04 (3.3E-04, 5.5E-04) |
| Egypt | 943,033,388 (681,530,844, 1,829,421,424) | 1.3E-04 (9.3E-05, 2.5E-04) | 147,525,343 (95,668,572, 265,625,748) | 2.0E-05 (1.3E-05, 3.6E-05) | 4,330,817,194 (2,895,158,226, 5,539,343,521) | 5.9E-04 (3.9E-04, 7.5E-04) | 77,790,319 (38,860,083, 187,571,165) | 1.1E-05 (5.3E-06, 2.6E-05) |
| El Salvador | 54,596,158 (41,826,228, 68,394,168) | 2.1E-03 (1.6E-03, 2.6E-03) | 10,717,036 (7,126,572, 14,279,932) | 4.1E-04 (2.7E-04, 5.4E-04) | 163,674,041 (118,168,216, 202,838,427) | 6.2E-03 (4.5E-03, 7.7E-03) | 9,945,369 (6,365,281, 13,469,300) | 3.8E-04 (2.4E-04, 5.1E-04) |
| Equatorial Guinea | 19,760,967 (11,915,812, 30,276,593) | 4.1E-06 (2.5E-06, 6.3E-06) | 2,799,767 (1,532,037, 4,527,910) | 5.8E-07 (3.2E-07, 9.4E-07) | 17,553,312 (8,554,014, 29,357,210) | 3.6E-06 (1.8E-06, 6.1E-06) | 2,160,049 (1,245,983, 3,611,497) | 4.5E-07 (2.6E-07, 7.5E-07) |
| Estonia | 440,080,231 (377,710,951, 503,753,998) | 1.7E-02 (1.5E-02, 2.0E-02) | 284,684,778 (244,749,184, 326,581,089) | 1.1E-02 (9.5E-03, 1.3E-02) | 467,064,890 (397,367,478, 529,620,267) | 1.8E-02 (1.5E-02, 2.1E-02) | 31,612,370 (23,690,091, 39,863,781) | 1.2E-03 (9.2E-04, 1.5E-03) |
| Eswatini | 19,125,356 (11,307,387, 27,918,603) | 4.1E-04 (2.4E-04, 6.0E-04) | 4,161,036 (2,655,923, 6,447,616) | 8.9E-05 (5.7E-05, 1.4E-04) | 13,462,840 (8,497,617, 20,164,420) | 2.9E-04 (1.8E-04, 4.3E-04) | 2,252,138 (1,364,833, 3,563,936) | 4.8E-05 (2.9E-05, 7.6E-05) |
| Ethiopia | 194,779,393 (144,950,556, 248,537,765) | 9.2E-05 (6.9E-05, 1.2E-04) | 12,658,578 (6,479,332, 22,453,526) | 6.0E-06 (3.1E-06, 1.1E-05) | 432,029,506 (301,511,543, 616,910,594) | 2.0E-04 (1.4E-04, 2.9E-04) | 78,512,428 (46,633,252, 127,509,405) | 3.7E-05 (2.2E-05, 6.0E-05) |
| Finland | 2,481,763,278 (2,220,866,527, 2,725,720,246) | 1.1E-02 (9.6E-03, 1.2E-02) | 1,418,355,015 (1,268,965,033, 1,561,048,507) | 6.2E-03 (5.5E-03, 6.8E-03) | 1,941,338,362 (1,774,917,473, 2,097,548,186) | 8.4E-03 (7.7E-03, 9.1E-03) | 129,369,870 (107,853,562, 151,596,772) | 5.6E-04 (4.7E-04, 6.6E-04) |
| France | 23,295,500,105 (20,842,202,732, 25,892,777,969) | 1.0E-02 (9.1E-03, 1.1E-02) | 14,881,191,804 (12,803,476,395, 16,852,758,873) | 6.5E-03 (5.6E-03, 7.4E-03) | 27,300,803,421 (24,386,523,900, 29,996,597,857) | 1.2E-02 (1.1E-02, 1.3E-02) | 1,530,801,439 (1,254,419,361, 1,878,221,615) | 6.7E-04 (5.5E-04, 8.2E-04) |
| Gabon | 43,601,144 (30,521,597, 60,289,529) | 7.6E-06 (5.3E-06, 1.1E-05) | 8,768,000 (4,824,718, 13,034,993) | 1.5E-06 (8.4E-07, 2.3E-06) | 29,764,806 (17,263,246, 44,468,923) | 5.2E-06 (3.0E-06, 7.8E-06) | 3,885,585 (2,471,592, 6,068,833) | 6.8E-07 (4.3E-07, 1.1E-06) |
| Gambia | 3,381,824 (2,422,077, 4,488,646) | 5.2E-05 (3.7E-05, 6.9E-05) | 49,917 (29,092, 74,218) | 7.6E-07 (4.4E-07, 1.1E-06) | 941,948 (592,758, 1,425,056) | 1.4E-05 (9.1E-06, 2.2E-05) | 573,088 (363,840, 982,717) | 8.8E-06 (5.6E-06, 1.5E-05) |
| Georgia | 135,260,738 (112,531,224, 161,840,103) | 2.6E-03 (2.2E-03, 3.1E-03) | 66,437,092 (57,049,136, 76,999,870) | 1.3E-03 (1.1E-03, 1.5E-03) | 233,002,863 (208,863,993, 258,839,728) | 4.5E-03 (4.1E-03, 5.0E-03) | 45,141,760 (36,830,506, 55,021,334) | 8.8E-04 (7.1E-04, 1.1E-03) |
| Germany | 39,794,667,133 (35,358,091,166, 43,632,532,958) | 1.2E-02 (1.1E-02, 1.4E-02) | 24,254,548,585 (21,784,046,136, 26,304,643,884) | 7.5E-03 (6.8E-03, 8.2E-03) | 46,345,635,784 (42,301,045,113, 49,781,718,221) | 1.4E-02 (1.3E-02, 1.5E-02) | 2,630,060,510 (2,180,395,611, 3,158,793,857) | 8.2E-04 (6.8E-04, 9.8E-04) |
| Ghana | 145,134,562 (107,789,875, 183,971,194) | 8.3E-04 (6.2E-04, 1.1E-03) | 3,257,395 (900,797, 6,458,601) | 1.9E-05 (5.2E-06, 3.7E-05) | 119,142,452 (78,120,596, 173,828,562) | 6.8E-04 (4.5E-04, 1.0E-03) | 1,182,193 (589,883, 1,771,919) | 6.8E-06 (3.4E-06, 1.0E-05) |
| Greece | 1,597,550,070 (1,451,933,130, 1,720,513,293) | 8.8E-03 (8.0E-03, 9.5E-03) | 1,381,153,358 (1,261,549,560, 1,465,359,114) | 7.6E-03 (7.0E-03, 8.1E-03) | 3,486,773,680 (3,234,478,992, 3,716,066,256) | 1.9E-02 (1.8E-02, 2.1E-02) | 453,358,137 (407,891,684, 498,761,823) | 2.5E-03 (2.3E-03, 2.8E-03) |
| Grenada | 6,322,066 (5,469,859, 7,284,346) | 2.8E-03 (2.4E-03, 3.2E-03) | 1,761,802 (1,520,539, 2,014,473) | 7.8E-04 (6.8E-04, 9.0E-04) | 2,922,222 (2,550,529, 3,313,518) | 1.3E-03 (1.1E-03, 1.5E-03) | 181,448 (147,752, 220,654) | 8.1E-05 (6.6E-05, 9.8E-05) |
| Guatemala | 89,434,624 (76,550,578, 103,469,102) | 1.6E-04 (1.4E-04, 1.9E-04) | 21,068,981 (17,952,965, 24,699,508) | 3.9E-05 (3.3E-05, 4.5E-05) | 420,342,912 (361,725,464, 489,592,297) | 7.7E-04 (6.6E-04, 9.0E-04) | 15,480,500 (12,909,307, 18,049,043) | 2.8E-05 (2.4E-05, 3.3E-05) |
| Guinea | 19,459,865 (13,398,132, 30,227,669) | 2.6E-07 (1.8E-07, 4.1E-07) | 754,621 (410,174, 1,158,201) | 1.0E-08 (5.5E-09, 1.6E-08) | 6,768,089 (3,334,637, 12,412,217) | 9.1E-08 (4.5E-08, 1.7E-07) | 5,813,853 (3,457,832, 9,175,725) | 7.8E-08 (4.6E-08, 1.2E-07) |
| Guyana | 28,442,460 (21,734,652, 36,245,900) | 1.6E-05 (1.2E-05, 2.0E-05) | 7,796,916 (5,911,895, 10,142,184) | 4.3E-06 (3.3E-06, 5.6E-06) | 40,583,340 (31,010,307, 52,185,777) | 2.3E-05 (1.7E-05, 2.9E-05) | 5,321,497 (4,069,521, 6,874,128) | 3.0E-06 (2.3E-06, 3.8E-06) |
| Honduras | 17,650,066 (11,917,676, 27,163,666) | 7.8E-05 (5.3E-05, 1.2E-04) | 9,302,789 (6,030,552, 13,892,274) | 4.1E-05 (2.7E-05, 6.1E-05) | 77,597,800 (52,420,068, 116,612,663) | 3.4E-04 (2.3E-04, 5.2E-04) | 3,266,561 (1,982,497, 4,961,129) | 1.4E-05 (8.8E-06, 2.2E-05) |
| Hungary | 2,316,384,792 (1,957,349,803, 2,686,006,280) | 5.5E-05 (4.7E-05, 6.4E-05) | 1,195,462,879 (1,023,589,876, 1,382,852,844) | 2.8E-05 (2.4E-05, 3.3E-05) | 3,403,635,370 (2,847,421,722, 4,074,431,074) | 8.1E-05 (6.8E-05, 9.7E-05) | 186,139,306 (147,893,931, 237,088,406) | 4.4E-06 (3.5E-06, 5.6E-06) |
| Iceland | 95,514,201 (84,319,998, 107,663,051) | 3.6E-05 (3.2E-05, 4.0E-05) | 64,258,801 (56,294,601, 72,057,266) | 2.4E-05 (2.1E-05, 2.7E-05) | 99,250,582 (88,998,394, 109,863,583) | 3.7E-05 (3.3E-05, 4.1E-05) | 6,101,254 (4,972,659, 7,437,900) | 2.3E-06 (1.9E-06, 2.8E-06) |
| India | 7,539,371,734 (6,647,711,863, 8,796,196,641) | 5.0E-05 (4.4E-05, 5.9E-05) | 2,569,359,213 (2,026,098,054, 3,413,849,057) | 1.7E-05 (1.3E-05, 2.3E-05) | 10,919,655,939 (8,737,215,737, 13,185,362,006) | 7.3E-05 (5.8E-05, 8.8E-05) | 1,852,114,835 (1,279,827,756, 2,729,097,222) | 1.2E-05 (8.5E-06, 1.8E-05) |
| Indonesia | 3,409,973,736 (2,534,349,276, 4,847,677,638) | 3.1E-07 (2.3E-07, 4.4E-07) | 458,966,680 (300,611,040, 842,589,447) | 4.1E-08 (2.7E-08, 7.6E-08) | 8,184,292,320 (6,599,374,765, 10,419,578,879) | 7.4E-07 (5.9E-07, 9.4E-07) | 331,082,935 (191,540,427, 717,693,740) | 3.0E-08 (1.7E-08, 6.5E-08) |
| Iran (Islamic Republic of) | 969,147,719 (782,653,210, 1,081,301,056) | 6.4E-08 (5.1E-08, 7.1E-08) | 327,578,189 (208,503,900, 389,204,740) | 2.1E-08 (1.4E-08, 2.6E-08) | 2,981,832,104 (1,978,984,163, 3,486,725,230) | 2.0E-07 (1.3E-07, 2.3E-07) | 188,944,891 (96,370,894, 233,117,769) | 1.2E-08 (6.3E-09, 1.5E-08) |
| Iraq | 540,406,878 (397,231,392, 717,010,512) | 2.7E-06 (2.0E-06, 3.6E-06) | 132,502,578 (85,495,871, 189,555,871) | 6.7E-07 (4.3E-07, 9.6E-07) | 1,191,382,695 (891,434,545, 1,552,804,220) | 6.0E-06 (4.5E-06, 7.8E-06) | 104,174,490 (50,712,396, 155,914,855) | 5.3E-07 (2.6E-07, 7.9E-07) |
| Ireland | 1,949,924,784 (1,727,880,979, 2,157,385,025) | 4.5E-03 (4.0E-03, 5.0E-03) | 1,195,713,850 (1,057,579,344, 1,342,786,030) | 2.8E-03 (2.4E-03, 3.1E-03) | 1,696,566,789 (1,550,348,202, 1,855,658,928) | 3.9E-03 (3.6E-03, 4.3E-03) | 174,287,319 (143,200,051, 207,968,193) | 4.0E-04 (3.3E-04, 4.8E-04) |
| Israel | 2,028,079,466 (1,789,561,854, 2,208,459,996) | 1.4E-03 (1.2E-03, 1.5E-03) | 940,704,522 (840,270,819, 1,028,851,130) | 6.3E-04 (5.6E-04, 6.9E-04) | 1,978,459,507 (1,818,294,952, 2,130,630,370) | 1.3E-03 (1.2E-03, 1.4E-03) | 127,024,552 (105,244,146, 149,112,758) | 8.5E-05 (7.1E-05, 1.0E-04) |
| Italy | 22,572,230,862 (20,281,349,633, 24,320,320,519) | 1.3E-02 (1.2E-02, 1.4E-02) | 14,261,082,704 (12,749,726,497, 15,329,208,352) | 8.4E-03 (7.5E-03, 9.0E-03) | 25,552,103,695 (23,327,455,964, 27,110,907,580) | 1.5E-02 (1.4E-02, 1.6E-02) | 2,198,323,428 (1,999,615,807, 2,421,052,805) | 1.3E-03 (1.2E-03, 1.4E-03) |
| Jamaica | 47,538,194 (36,426,802, 61,433,342) | 6.5E-05 (4.9E-05, 8.3E-05) | 25,570,621 (19,412,203, 33,001,006) | 3.5E-05 (2.6E-05, 4.5E-05) | 37,113,083 (28,433,223, 48,239,215) | 5.0E-05 (3.9E-05, 6.6E-05) | 2,409,234 (1,740,602, 3,285,550) | 3.3E-06 (2.4E-06, 4.5E-06) |
| Japan | 44,998,339,248 (39,657,197,262, 48,563,052,200) | 8.3E-05 (7.3E-05, 8.9E-05) | 15,786,265,844 (13,690,315,533, 17,104,926,589) | 2.9E-05 (2.5E-05, 3.1E-05) | 34,926,244,496 (31,835,582,720, 36,854,209,000) | 6.4E-05 (5.9E-05, 6.8E-05) | 878,241,080 (806,042,162, 932,818,888) | 1.6E-06 (1.5E-06, 1.7E-06) |
| Jordan | 104,512,818 (80,843,391, 131,795,798) | 3.4E-03 (2.6E-03, 4.3E-03) | 17,219,423 (10,662,157, 25,323,904) | 5.6E-04 (3.5E-04, 8.2E-04) | 163,266,059 (117,760,009, 214,575,619) | 5.3E-03 (3.8E-03, 7.0E-03) | 6,708,758 (4,114,656, 9,708,311) | 2.2E-04 (1.3E-04, 3.1E-04) |
| Kazakhstan | 576,476,330 (489,562,115, 673,173,371) | 3.9E-05 (3.3E-05, 4.5E-05) | 232,047,963 (193,859,113, 273,790,436) | 1.6E-05 (1.3E-05, 1.8E-05) | 1,331,986,412 (1,196,466,093, 1,485,385,120) | 8.9E-05 (8.0E-05, 9.9E-05) | 145,894,450 (121,034,355, 173,503,050) | 9.8E-06 (8.1E-06, 1.2E-05) |
| Kenya | 139,504,284 (111,361,787, 171,153,327) | 1.5E-05 (1.2E-05, 1.8E-05) | 34,176,710 (22,129,054, 47,994,108) | 3.6E-06 (2.4E-06, 5.1E-06) | 131,218,133 (91,636,109, 178,128,213) | 1.4E-05 (9.8E-06, 1.9E-05) | 32,039,758 (18,864,120, 49,617,933) | 3.4E-06 (2.0E-06, 5.3E-06) |
| Kiribati | 88,534 (48,050, 121,613) | 2.6E-04 (1.4E-04, 3.6E-04) | 5,528 (2,940, 8,229) | 1.6E-05 (8.7E-06, 2.4E-05) | 595,024 (327,674, 896,168) | 1.8E-03 (9.7E-04, 2.7E-03) | 9,783 (3,660, 16,361) | 2.9E-05 (1.1E-05, 4.9E-05) |
| Kuwait | 534,093,342 (448,595,343, 632,198,653) | 1.4E-02 (1.2E-02, 1.6E-02) | 116,762,253 (96,673,577, 138,544,702) | 3.0E-03 (2.5E-03, 3.6E-03) | 557,746,400 (456,695,173, 673,577,890) | 1.4E-02 (1.2E-02, 1.7E-02) | 40,767,357 (32,102,670, 49,989,129) | 1.1E-03 (8.3E-04, 1.3E-03) |
| Kyrgyzstan | 10,696,316 (8,422,764, 13,416,814) | 5.9E-05 (4.6E-05, 7.3E-05) | 2,315,802 (1,784,866, 2,915,058) | 1.3E-05 (9.8E-06, 1.6E-05) | 33,650,629 (27,740,378, 39,962,909) | 1.8E-04 (1.5E-04, 2.2E-04) | 3,118,774 (2,294,436, 4,096,553) | 1.7E-05 (1.3E-05, 2.2E-05) |
| Lao People's Democratic Republic | 44,699,530 (31,458,843, 68,394,719) | 3.4E-07 (2.4E-07, 5.2E-07) | 4,172,078 (2,226,939, 8,102,020) | 3.2E-08 (1.7E-08, 6.2E-08) | 118,291,023 (83,370,768, 164,000,404) | 9.0E-07 (6.3E-07, 1.2E-06) | 5,015,722 (2,618,672, 11,546,426) | 3.8E-08 (2.0E-08, 8.8E-08) |
| Latvia | 486,845,096 (415,222,335, 561,859,276) | 1.7E-02 (1.5E-02, 2.0E-02) | 326,623,032 (278,614,370, 384,402,165) | 1.2E-02 (1.0E-02, 1.4E-02) | 602,569,771 (516,442,072, 696,224,391) | 2.2E-02 (1.8E-02, 2.5E-02) | 52,333,166 (39,081,399, 67,304,672) | 1.9E-03 (1.4E-03, 2.4E-03) |
| Lebanon | 142,300,190 (110,184,178, 179,175,138) | 3.6E-06 (2.8E-06, 4.5E-06) | 48,512,790 (33,843,794, 66,562,048) | 1.2E-06 (8.5E-07, 1.7E-06) | 121,898,865 (96,353,726, 157,176,938) | 3.0E-06 (2.4E-06, 3.9E-06) | 19,006,406 (11,460,716, 25,269,564) | 4.8E-07 (2.9E-07, 6.3E-07) |
| Lesotho | 5,073,741 (3,430,433, 6,905,587) | 2.5E-04 (1.7E-04, 3.5E-04) | 939,895 (587,632, 1,437,173) | 4.7E-05 (2.9E-05, 7.2E-05) | 4,280,157 (3,035,584, 5,786,426) | 2.1E-04 (1.5E-04, 2.9E-04) | 733,623 (475,520, 1,202,837) | 3.7E-05 (2.4E-05, 6.0E-05) |
| Liberia | 3,805,198 (2,560,489, 5,119,048) | 1.2E-03 (7.9E-04, 1.6E-03) | 38,431 (10,496, 80,277) | 1.2E-05 (3.2E-06, 2.5E-05) | 2,784,591 (1,280,032, 4,215,553) | 8.6E-04 (3.9E-04, 1.3E-03) | 205,369 (124,735, 324,577) | 6.3E-05 (3.8E-05, 1.0E-04) |
| Libya | 314,485,143 (235,325,513, 414,697,567) | 3.8E-03 (2.8E-03, 5.0E-03) | 67,638,325 (44,223,451, 97,062,686) | 8.2E-04 (5.3E-04, 1.2E-03) | 362,714,480 (261,044,500, 501,179,887) | 4.4E-03 (3.2E-03, 6.1E-03) | 78,031,359 (47,086,564, 113,836,485) | 9.4E-04 (5.7E-04, 1.4E-03) |
| Lithuania | 762,151,110 (664,070,426, 864,550,147) | 1.7E-02 (1.4E-02, 1.9E-02) | 562,905,087 (492,077,109, 638,851,030) | 1.2E-02 (1.1E-02, 1.4E-02) | 1,090,638,249 (944,834,126, 1,228,745,902) | 2.4E-02 (2.0E-02, 2.7E-02) | 90,514,190 (72,890,389, 109,375,272) | 2.0E-03 (1.6E-03, 2.4E-03) |
| Luxembourg | 306,782,823 (274,097,309, 338,117,892) | 4.8E-03 (4.3E-03, 5.3E-03) | 191,106,630 (169,174,162, 210,729,894) | 3.0E-03 (2.7E-03, 3.3E-03) | 420,416,346 (377,825,287, 465,538,061) | 6.6E-03 (5.9E-03, 7.3E-03) | 17,566,826 (15,198,960, 20,204,834) | 2.8E-04 (2.4E-04, 3.2E-04) |
| Madagascar | 21,597,492 (15,506,411, 28,560,846) | 1.0E-06 (7.2E-07, 1.3E-06) | 2,125,354 (1,064,888, 3,127,099) | 9.9E-08 (5.0E-08, 1.5E-07) | 17,718,636 (11,472,975, 24,627,131) | 8.2E-07 (5.3E-07, 1.1E-06) | 6,725,522 (3,908,655, 10,203,282) | 3.1E-07 (1.8E-07, 4.7E-07) |
| Malawi | 55,880,785 (35,753,567, 84,114,428) | 7.4E-06 (4.7E-06, 1.1E-05) | 1,083,462 (646,713, 1,569,950) | 1.4E-07 (8.5E-08, 2.1E-07) | 5,542,680 (2,668,850, 9,925,880) | 7.3E-07 (3.5E-07, 1.3E-06) | 3,266,716 (1,772,292, 4,931,635) | 4.3E-07 (2.3E-07, 6.5E-07) |
| Malaysia | 1,788,853,679 (1,437,700,995, 2,410,336,373) | 1.3E-03 (1.0E-03, 1.7E-03) | 372,451,238 (295,240,545, 536,551,184) | 2.7E-04 (2.1E-04, 3.9E-04) | 3,037,265,785 (2,485,723,587, 3,644,559,051) | 2.2E-03 (1.8E-03, 2.6E-03) | 168,426,562 (96,865,496, 212,098,755) | 1.2E-04 (7.0E-05, 1.5E-04) |
| Maldives | 4,341,204 (3,411,166, 5,281,307) | 5.3E-05 (4.2E-05, 6.5E-05) | 1,127,411 (785,825, 1,587,917) | 1.4E-05 (9.7E-06, 2.0E-05) | 9,515,863 (6,766,219, 12,868,200) | 1.2E-04 (8.3E-05, 1.6E-04) | 604,048 (376,241, 1,077,904) | 7.4E-06 (4.6E-06, 1.3E-05) |
| Mali | 12,891,510 (8,998,026, 19,073,899) | 2.3E-06 (1.6E-06, 3.5E-06) | 167 (73, 252) | 3.0E-11 (1.3E-11, 4.6E-11) | 12,935,906 (7,249,560, 22,426,019) | 2.3E-06 (1.3E-06, 4.1E-06) | 6,724,325 (4,124,263, 10,350,290) | 1.2E-06 (7.5E-07, 1.9E-06) |
| Malta | 133,503,466 (117,574,599, 149,325,113) | 9.8E-03 (8.6E-03, 1.1E-02) | 61,016,850 (52,317,716, 69,720,813) | 4.5E-03 (3.8E-03, 5.1E-03) | 127,834,244 (113,170,051, 143,908,707) | 9.4E-03 (8.3E-03, 1.1E-02) | 12,654,850 (10,560,089, 15,522,429) | 9.3E-04 (7.8E-04, 1.1E-03) |
| Marshall Islands | 294,337 (206,517, 409,289) | 1.3E-03 (9.3E-04, 1.8E-03) | 24,875 (11,799, 45,108) | 1.1E-04 (5.3E-05, 2.0E-04) | 591,622 (382,668, 834,125) | 2.7E-03 (1.7E-03, 3.8E-03) | 13,945 (7,149, 20,204) | 6.3E-05 (3.2E-05, 9.1E-05) |
| Mauritania | 13,074,903 (9,539,331, 17,366,164) | 1.6E-04 (1.2E-04, 2.2E-04) | 321,732 (97,644, 551,001) | 4.0E-06 (1.2E-06, 6.8E-06) | 10,545,913 (5,281,439, 16,430,063) | 1.3E-04 (6.6E-05, 2.0E-04) | 658,459 (413,185, 1,120,595) | 8.2E-06 (5.1E-06, 1.4E-05) |
| Mauritius | 61,485,010 (56,390,490, 64,982,910) | 1.4E-04 (1.2E-04, 1.4E-04) | 37,645,708 (34,390,780, 39,807,724) | 8.3E-05 (7.6E-05, 8.8E-05) | 78,605,378 (72,962,371, 82,687,416) | 1.7E-04 (1.6E-04, 1.8E-04) | 9,926,317 (8,906,170, 10,895,898) | 2.2E-05 (2.0E-05, 2.4E-05) |
| Mexico | 3,812,458,214 (3,356,765,853, 4,253,303,077) | 1.6E-04 (1.4E-04, 1.8E-04) | 1,446,794,823 (1,273,280,401, 1,632,999,994) | 6.2E-05 (5.5E-05, 7.0E-05) | 8,213,105,287 (7,482,940,491, 8,974,072,470) | 3.5E-04 (3.2E-04, 3.9E-04) | 772,766,564 (686,951,220, 862,419,129) | 3.3E-05 (2.9E-05, 3.7E-05) |
| Micronesia (Federated States of) | 256,081 (181,311, 345,214) | 1.0E-03 (7.1E-04, 1.3E-03) | 24,767 (12,139, 41,095) | 9.6E-05 (4.7E-05, 1.6E-04) | 491,459 (324,640, 674,190) | 1.9E-03 (1.3E-03, 2.6E-03) | 9,891 (5,380, 14,698) | 3.8E-05 (2.1E-05, 5.7E-05) |
| Mongolia | 26,404,414 (20,235,637, 35,235,088) | 9.7E-07 (7.5E-07, 1.3E-06) | 4,133,086 (2,908,022, 5,689,069) | 1.5E-07 (1.1E-07, 2.1E-07) | 60,770,490 (44,815,645, 77,793,252) | 2.2E-06 (1.7E-06, 2.9E-06) | 8,272,697 (5,040,265, 12,056,336) | 3.1E-07 (1.9E-07, 4.5E-07) |
| Montenegro | 25,624,183 (20,360,619, 31,472,426) | 8.6E-03 (6.9E-03, 1.1E-02) | 12,598,135 (8,816,312, 16,667,848) | 4.2E-03 (3.0E-03, 5.6E-03) | 47,380,169 (37,976,859, 60,026,466) | 1.6E-02 (1.3E-02, 2.0E-02) | 8,219,159 (5,520,038, 11,730,455) | 2.8E-03 (1.9E-03, 4.0E-03) |
| Morocco | 313,528,799 (235,177,851, 403,905,908) | 2.7E-04 (2.0E-04, 3.4E-04) | 66,434,467 (39,723,680, 104,412,255) | 5.7E-05 (3.4E-05, 8.9E-05) | 122,102,982 (84,957,666, 164,494,810) | 1.0E-04 (7.2E-05, 1.4E-04) | 66,007,043 (40,817,450, 101,691,501) | 5.6E-05 (3.5E-05, 8.7E-05) |
| Mozambique | 9,947,876 (7,048,826, 13,062,192) | 1.0E-05 (7.2E-06, 1.3E-05) | 2,681,558 (1,357,814, 4,338,446) | 2.7E-06 (1.4E-06, 4.4E-06) | 31,122,186 (17,558,714, 55,704,503) | 3.2E-05 (1.8E-05, 5.7E-05) | 8,659,369 (4,182,943, 13,800,974) | 8.8E-06 (4.3E-06, 1.4E-05) |
| Myanmar | 208,758,398 (151,357,372, 298,322,996) | 2.6E-06 (1.9E-06, 3.8E-06) | 22,500,170 (13,726,356, 42,947,801) | 2.9E-07 (1.7E-07, 5.4E-07) | 523,195,536 (386,641,438, 695,169,214) | 6.6E-06 (4.9E-06, 8.8E-06) | 20,267,812 (11,005,682, 45,599,343) | 2.6E-07 (1.4E-07, 5.8E-07) |
| Namibia | 34,659,941 (24,792,140, 49,410,466) | 2.5E-04 (1.8E-04, 3.6E-04) | 6,322,911 (4,252,426, 9,141,138) | 4.6E-05 (3.1E-05, 6.6E-05) | 21,079,887 (13,982,776, 30,925,648) | 1.5E-04 (1.0E-04, 2.2E-04) | 5,745,357 (3,315,919, 8,707,728) | 4.2E-05 (2.4E-05, 6.3E-05) |
| Nauru | 623,823 (404,117, 859,287) | 4.5E-03 (2.9E-03, 6.2E-03) | 55,261 (24,083, 106,861) | 4.0E-04 (1.7E-04, 7.7E-04) | 1,210,342 (782,467, 1,802,859) | 8.7E-03 (5.6E-03, 1.3E-02) | 21,468 (10,064, 32,850) | 1.5E-04 (7.2E-05, 2.4E-04) |
| Nepal | 70,580,868 (51,122,830, 113,896,452) | 2.9E-05 (2.1E-05, 4.8E-05) | 16,856,823 (8,519,773, 28,471,759) | 7.0E-06 (3.6E-06, 1.2E-05) | 117,536,608 (85,656,335, 163,530,652) | 4.9E-05 (3.6E-05, 6.8E-05) | 21,259,219 (11,409,225, 45,319,152) | 8.9E-06 (4.8E-06, 1.9E-05) |
| Netherlands | 6,354,617,566 (5,675,498,887, 7,021,664,421) | 8.2E-03 (7.3E-03, 9.1E-03) | 5,381,750,456 (4,819,007,620, 5,817,672,808) | 7.0E-03 (6.2E-03, 7.5E-03) | 6,832,406,588 (6,286,234,303, 7,356,141,200) | 8.8E-03 (8.1E-03, 9.5E-03) | 569,329,889 (481,612,106, 670,066,900) | 7.4E-04 (6.2E-04, 8.7E-04) |
| New Zealand | 1,424,022,932 (1,278,039,581, 1,551,655,407) | 5.3E-03 (4.7E-03, 5.8E-03) | 880,551,714 (777,791,024, 963,341,770) | 3.3E-03 (2.9E-03, 3.6E-03) | 1,296,515,139 (1,211,655,495, 1,370,798,859) | 4.8E-03 (4.5E-03, 5.1E-03) | 63,675,603 (53,516,193, 75,355,660) | 2.4E-04 (2.0E-04, 2.8E-04) |
| Nicaragua | 18,350,747 (15,233,529, 22,362,213) | 9.7E-05 (8.0E-05, 1.2E-04) | 3,467,478 (2,430,743, 4,491,470) | 1.8E-05 (1.3E-05, 2.4E-05) | 51,221,124 (35,565,826, 63,012,064) | 2.7E-04 (1.9E-04, 3.3E-04) | 2,858,220 (1,899,614, 4,109,650) | 1.5E-05 (1.0E-05, 2.2E-05) |
| Niger | 9,625,676 (5,859,205, 14,327,206) | 1.3E-06 (7.7E-07, 1.9E-06) | 88,955 (25,750, 170,328) | 1.2E-08 (3.4E-09, 2.2E-08) | 11,260,941 (4,960,483, 18,038,398) | 1.5E-06 (6.5E-07, 2.4E-06) | 700,954 (402,938, 1,222,299) | 9.2E-08 (5.3E-08, 1.6E-07) |
| Nigeria | 994,538,820 (633,605,997, 1,327,308,541) | 1.4E-05 (8.6E-06, 1.8E-05) | 90,054,270 (30,809,154, 142,861,075) | 1.2E-06 (4.2E-07, 1.9E-06) | 699,878,018 (366,052,680, 973,972,155) | 9.5E-06 (5.0E-06, 1.3E-05) | 688,626,619 (204,486,136, 1,145,137,857) | 9.4E-06 (2.8E-06, 1.6E-05) |
| North Macedonia | 66,236,177 (51,022,327, 82,284,858) | 1.4E-04 (1.1E-04, 1.8E-04) | 29,938,645 (19,382,918, 39,704,901) | 6.4E-05 (4.1E-05, 8.5E-05) | 133,758,084 (94,549,197, 169,567,730) | 2.9E-04 (2.0E-04, 3.6E-04) | 22,806,231 (15,165,443, 37,100,767) | 4.9E-05 (3.2E-05, 7.9E-05) |
| Norway | 2,484,258,522 (2,285,202,252, 2,670,416,240) | 5.7E-04 (5.3E-04, 6.2E-04) | 2,207,251,227 (1,996,244,896, 2,360,285,992) | 5.1E-04 (4.6E-04, 5.5E-04) | 2,700,407,056 (2,492,676,702, 2,859,862,632) | 6.2E-04 (5.8E-04, 6.6E-04) | 208,624,623 (186,195,034, 232,854,047) | 4.8E-05 (4.3E-05, 5.4E-05) |
| Oman | 306,463,846 (234,549,001, 388,193,109) | 8.8E-03 (6.8E-03, 1.1E-02) | 65,457,728 (42,696,268, 97,397,337) | 1.9E-03 (1.2E-03, 2.8E-03) | 305,494,796 (228,944,353, 398,171,934) | 8.8E-03 (6.6E-03, 1.1E-02) | 56,931,498 (26,840,684, 84,782,687) | 1.6E-03 (7.7E-04, 2.4E-03) |
| Pakistan | 1,542,330,606 (1,193,230,238, 1,956,057,196) | 4.2E-05 (3.3E-05, 5.3E-05) | 239,822,832 (167,929,638, 346,674,303) | 6.6E-06 (4.6E-06, 9.5E-06) | 1,522,542,844 (1,128,415,231, 2,092,154,214) | 4.2E-05 (3.1E-05, 5.7E-05) | 561,194,246 (377,568,103, 808,467,041) | 1.5E-05 (1.0E-05, 2.2E-05) |
| Panama | 367,764,192 (298,112,595, 439,450,217) | 5.5E-03 (4.5E-03, 6.6E-03) | 185,388,082 (146,115,783, 226,413,621) | 2.8E-03 (2.2E-03, 3.4E-03) | 688,138,129 (572,302,508, 815,371,165) | 1.0E-02 (8.6E-03, 1.2E-02) | 31,928,622 (25,658,803, 38,499,045) | 4.8E-04 (3.9E-04, 5.8E-04) |
| Papua New Guinea | 12,490,254 (8,315,170, 17,883,384) | 2.0E-04 (1.3E-04, 2.8E-04) | 925,884 (354,486, 1,705,683) | 1.5E-05 (5.6E-06, 2.7E-05) | 39,772,630 (23,491,002, 55,772,559) | 6.3E-04 (3.7E-04, 8.8E-04) | 808,142 (409,096, 1,257,032) | 1.3E-05 (6.5E-06, 2.0E-05) |
| Paraguay | 106,610,144 (81,031,483, 138,233,328) | 5.0E-07 (3.8E-07, 6.4E-07) | 34,581,433 (23,009,107, 48,925,140) | 1.6E-07 (1.1E-07, 2.3E-07) | 223,445,626 (162,931,494, 287,695,201) | 1.0E-06 (7.6E-07, 1.3E-06) | 14,346,192 (9,266,249, 21,044,049) | 6.7E-08 (4.3E-08, 9.8E-08) |
| Peru | 927,132,346 (693,086,383, 1,189,556,356) | 1.7E-03 (1.3E-03, 2.2E-03) | 225,709,069 (157,249,206, 325,909,777) | 4.1E-04 (2.8E-04, 5.9E-04) | 1,214,966,072 (763,876,658, 1,607,981,813) | 2.2E-03 (1.4E-03, 2.9E-03) | 49,428,095 (30,188,824, 69,836,382) | 8.9E-05 (5.5E-05, 1.3E-04) |
| Philippines | 835,043,523 (702,974,211, 1,021,887,203) | 4.5E-05 (3.8E-05, 5.5E-05) | 99,050,983 (73,277,299, 129,030,205) | 5.3E-06 (4.0E-06, 7.0E-06) | 1,914,813,166 (1,540,443,941, 2,247,428,297) | 1.0E-04 (8.3E-05, 1.2E-04) | 48,207,200 (30,588,961, 62,595,037) | 2.6E-06 (1.6E-06, 3.4E-06) |
| Poland | 7,756,099,662 (7,085,845,343, 8,395,134,277) | 3.4E-03 (3.1E-03, 3.7E-03) | 5,589,579,919 (5,105,932,538, 6,104,076,829) | 2.5E-03 (2.3E-03, 2.7E-03) | 10,710,290,694 (9,766,031,974, 11,673,194,091) | 4.7E-03 (4.3E-03, 5.2E-03) | 900,017,092 (827,824,706, 974,573,691) | 4.0E-04 (3.7E-04, 4.3E-04) |
| Portugal | 2,773,826,891 (2,536,983,579, 3,032,562,590) | 1.4E-02 (1.3E-02, 1.5E-02) | 1,664,197,748 (1,478,646,880, 1,830,856,681) | 8.4E-03 (7.5E-03, 9.3E-03) | 2,762,172,260 (2,476,837,016, 2,987,561,672) | 1.4E-02 (1.3E-02, 1.5E-02) | 179,226,803 (147,589,266, 219,976,723) | 9.1E-04 (7.5E-04, 1.1E-03) |
| Puerto Rico | 593,828,527 (495,479,765, 704,039,642) | 6.4E-02 (5.3E-02, 7.6E-02) | 361,829,015 (300,902,183, 427,589,824) | 3.9E-02 (3.2E-02, 4.6E-02) | 573,557,916 (473,608,777, 673,022,641) | 6.2E-02 (5.1E-02, 7.3E-02) | 53,962,271 (42,299,073, 66,878,476) | 5.8E-03 (4.6E-03, 7.2E-03) |
| Qatar | 419,479,100 (301,562,635, 555,867,085) | 6.4E-04 (4.6E-04, 8.4E-04) | 110,016,207 (66,186,642, 181,225,420) | 1.7E-04 (1.0E-04, 2.8E-04) | 706,133,440 (474,533,058, 976,560,970) | 1.1E-03 (7.2E-04, 1.5E-03) | 56,982,778 (30,229,954, 88,109,342) | 8.7E-05 (4.6E-05, 1.3E-04) |
| Republic of Korea | 8,811,877,248 (6,056,692,883, 10,282,289,988) | 4.6E-06 (3.2E-06, 5.4E-06) | 3,836,129,096 (2,256,904,143, 5,066,518,143) | 2.0E-06 (1.2E-06, 2.6E-06) | 8,754,249,969 (5,620,269,215, 10,620,766,224) | 4.6E-06 (2.9E-06, 5.5E-06) | 204,165,277 (117,310,954, 285,574,125) | 1.1E-07 (6.1E-08, 1.5E-07) |
| Republic of Moldova | 119,557,818 (105,154,495, 136,928,174) | 8.6E-03 (7.6E-03, 9.9E-03) | 36,553,857 (32,651,965, 41,069,871) | 2.6E-03 (2.4E-03, 3.0E-03) | 122,702,040 (109,952,439, 138,329,400) | 8.9E-03 (7.9E-03, 1.0E-02) | 27,696,881 (23,432,608, 32,381,812) | 2.0E-03 (1.7E-03, 2.3E-03) |
| Romania | 3,234,191,848 (2,787,489,891, 3,671,820,606) | 3.6E-03 (3.1E-03, 4.1E-03) | 1,605,104,101 (1,377,438,515, 1,809,759,085) | 1.8E-03 (1.5E-03, 2.0E-03) | 4,900,961,497 (4,219,712,402, 5,538,699,637) | 5.5E-03 (4.7E-03, 6.2E-03) | 471,457,487 (361,315,442, 597,722,553) | 5.3E-04 (4.1E-04, 6.7E-04) |
| Russian Federation | 17,908,231,039 (16,520,349,578, 19,414,612,405) | 1.3E-04 (1.2E-04, 1.4E-04) | 9,045,999,933 (8,325,919,892, 9,781,612,343) | 6.7E-05 (6.1E-05, 7.2E-05) | 24,358,577,216 (22,663,598,408, 26,176,573,540) | 1.8E-04 (1.7E-04, 1.9E-04) | 3,281,086,926 (3,020,088,809, 3,561,304,355) | 2.4E-05 (2.2E-05, 2.6E-05) |
| Rwanda | 31,389,589 (22,522,203, 43,296,364) | 3.2E-06 (2.3E-06, 4.4E-06) | 4,047,292 (2,291,059, 6,079,459) | 4.1E-07 (2.3E-07, 6.2E-07) | 21,495,763 (12,534,142, 33,775,796) | 2.2E-06 (1.3E-06, 3.4E-06) | 7,177,819 (3,995,278, 11,944,426) | 7.3E-07 (4.1E-07, 1.2E-06) |
| Saint Kitts and Nevis | 7,159,122 (5,828,085, 8,665,768) | 3.5E-03 (2.9E-03, 4.3E-03) | 3,888,503 (3,089,066, 4,747,993) | 1.9E-03 (1.5E-03, 2.3E-03) | 6,116,135 (5,087,777, 7,210,758) | 3.0E-03 (2.5E-03, 3.5E-03) | 132,796 (108,367, 164,992) | 6.5E-05 (5.3E-05, 8.1E-05) |
| Saint Lucia | 9,575,706 (7,969,954, 11,544,644) | 2.0E-03 (1.7E-03, 2.4E-03) | 5,213,529 (4,288,583, 6,327,927) | 1.1E-03 (9.1E-04, 1.3E-03) | 6,601,430 (5,414,212, 7,942,050) | 1.4E-03 (1.1E-03, 1.7E-03) | 1,035,788 (841,573, 1,258,086) | 2.2E-04 (1.8E-04, 2.7E-04) |
| Saint Vincent and the Grenadines | 6,092,349 (5,328,399, 6,993,430) | 2.6E-03 (2.3E-03, 3.0E-03) | 1,353,895 (1,181,556, 1,546,961) | 5.8E-04 (5.1E-04, 6.6E-04) | 4,250,697 (3,695,159, 4,898,829) | 1.8E-03 (1.6E-03, 2.1E-03) | 474,416 (405,801, 553,345) | 2.0E-04 (1.7E-04, 2.4E-04) |
| Samoa | 1,135,213 (893,393, 1,555,448) | 5.8E-04 (4.5E-04, 7.9E-04) | 111,328 (74,491, 178,544) | 5.6E-05 (3.8E-05, 9.1E-05) | 1,643,637 (1,226,223, 2,285,865) | 8.3E-04 (6.2E-04, 1.2E-03) | 289,052 (168,893, 566,396) | 1.5E-04 (8.6E-05, 2.9E-04) |
| Samoa | 1,135,213 (893,393, 1,555,448) | 5.8E-04 (4.5E-04, 7.9E-04) | 111,328 (74,491, 178,544) | 5.6E-05 (3.8E-05, 9.1E-05) | 1,643,637 (1,226,223, 2,285,865) | 8.3E-04 (6.2E-04, 1.2E-03) | 289,052 (168,893, 566,396) | 1.5E-04 (8.6E-05, 2.9E-04) |
| San Marino | 16,242,653 (10,777,902, 23,316,276) | 1.2E-02 (8.2E-03, 1.8E-02) | 4,869,854 (3,019,009, 7,321,275) | 3.7E-03 (2.3E-03, 5.6E-03) | 14,432,934 (9,459,908, 20,425,405) | 1.1E-02 (7.2E-03, 1.6E-02) | 1,687,116 (964,413, 2,647,685) | 1.3E-03 (7.4E-04, 2.0E-03) |
| Sao Tome and Principe | 654,733 (455,689, 905,285) | 1.6E-04 (1.1E-04, 2.2E-04) | 14,275 (4,362, 23,663) | 3.4E-06 (1.1E-06, 5.7E-06) | 260,969 (147,205, 450,017) | 6.3E-05 (3.5E-05, 1.1E-04) | 1,915 (950, 2,878) | 4.6E-07 (2.3E-07, 6.9E-07) |
| Saudi Arabia | 5,321,855,260 (3,900,344,279, 6,898,757,704) | 1.6E-03 (1.2E-03, 2.1E-03) | 879,595,551 (576,485,804, 1,210,564,617) | 2.7E-04 (1.8E-04, 3.7E-04) | 5,399,527,733 (3,801,502,883, 8,369,956,684) | 1.7E-03 (1.2E-03, 2.6E-03) | 615,985,794 (146,756,668, 1,091,715,411) | 1.9E-04 (4.5E-05, 3.4E-04) |
| Senegal | 30,429,164 (22,229,579, 40,051,858) | 2.1E-06 (1.6E-06, 2.8E-06) | 587,377 (185,079, 999,904) | 4.1E-08 (1.3E-08, 7.0E-08) | 25,841,280 (12,087,732, 42,140,379) | 1.8E-06 (8.5E-07, 3.0E-06) | 2,094,305 (1,337,446, 3,437,084) | 1.5E-07 (9.4E-08, 2.4E-07) |
| Serbia | 518,123,474 (411,727,056, 615,928,068) | 9.8E-05 (7.8E-05, 1.2E-04) | 297,476,697 (207,396,224, 391,930,849) | 5.6E-05 (3.9E-05, 7.4E-05) | 772,836,855 (575,193,957, 952,915,081) | 1.5E-04 (1.1E-04, 1.8E-04) | 89,184,723 (58,868,356, 133,063,516) | 1.7E-05 (1.1E-05, 2.5E-05) |
| Seychelles | 14,544,004 (12,073,601, 16,903,947) | 6.4E-04 (5.3E-04, 7.4E-04) | 4,226,993 (3,153,866, 5,519,657) | 1.9E-04 (1.4E-04, 2.4E-04) | 16,232,484 (13,117,941, 19,583,048) | 7.2E-04 (5.8E-04, 8.6E-04) | 1,210,841 (869,589, 1,746,964) | 5.3E-05 (3.8E-05, 7.7E-05) |
| Sierra Leone | 5,254,756 (3,794,938, 7,078,531) | 5.0E-07 (3.6E-07, 6.8E-07) | 58,293 (16,732, 103,457) | 5.6E-09 (1.6E-09, 9.9E-09) | 4,674,102 (2,214,766, 6,884,150) | 4.5E-07 (2.1E-07, 6.6E-07) | 302,930 (190,378, 486,841) | 2.9E-08 (1.8E-08, 4.7E-08) |
| Singapore | 2,100,374,393 (1,903,432,715, 2,280,767,061) | 4.1E-03 (3.8E-03, 4.5E-03) | 482,429,905 (435,861,143, 531,330,788) | 9.5E-04 (8.6E-04, 1.0E-03) | 2,027,100,625 (1,875,648,513, 2,177,232,109) | 4.0E-03 (3.7E-03, 4.3E-03) | 78,176,889 (64,651,418, 93,759,473) | 1.5E-04 (1.3E-04, 1.8E-04) |
| Slovakia | 1,002,876,129 (758,083,806, 1,205,837,391) | 1.1E-02 (8.3E-03, 1.3E-02) | 703,709,468 (477,565,360, 955,565,422) | 7.7E-03 (5.3E-03, 1.1E-02) | 1,359,430,324 (971,720,665, 1,745,720,690) | 1.5E-02 (1.1E-02, 1.9E-02) | 144,709,776 (93,800,000, 219,862,943) | 1.6E-03 (1.0E-03, 2.4E-03) |
| Slovenia | 807,779,263 (690,596,714, 943,611,444) | 1.8E-02 (1.5E-02, 2.1E-02) | 409,912,296 (351,150,495, 478,792,203) | 9.1E-03 (7.8E-03, 1.1E-02) | 688,748,647 (581,070,188, 800,262,190) | 1.5E-02 (1.3E-02, 1.8E-02) | 32,998,930 (24,701,323, 43,211,980) | 7.3E-04 (5.5E-04, 9.6E-04) |
| Solomon Islands | 601,702 (423,165, 831,601) | 5.7E-05 (4.0E-05, 7.8E-05) | 39,815 (18,295, 73,126) | 3.7E-06 (1.7E-06, 6.9E-06) | 1,548,105 (940,698, 2,128,657) | 1.5E-04 (8.8E-05, 2.0E-04) | 34,420 (18,616, 52,491) | 3.2E-06 (1.7E-06, 4.9E-06) |
| Somalia | 16,911,190 (11,333,151, 23,605,195) | 1.7E-03 (1.1E-03, 2.3E-03) | 687,108 (271,022, 1,489,715) | 6.8E-05 (2.7E-05, 1.5E-04) | 14,991,799 (9,427,240, 21,895,091) | 1.5E-03 (9.3E-04, 2.2E-03) | 6,760,071 (3,925,453, 10,620,843) | 6.6E-04 (3.9E-04, 1.0E-03) |
| Sri Lanka | 322,998,738 (216,177,025, 449,858,891) | 2.5E-05 (1.7E-05, 3.5E-05) | 131,009,715 (78,534,567, 194,342,748) | 1.0E-05 (6.0E-06, 1.5E-05) | 534,568,854 (355,970,340, 745,547,442) | 4.1E-05 (2.7E-05, 5.7E-05) | 35,857,335 (21,874,945, 62,108,042) | 2.8E-06 (1.7E-06, 4.8E-06) |
| Sudan | 54,348,051 (37,198,458, 81,105,997) | 2.6E-03 (1.8E-03, 3.9E-03) | 7,278,879 (4,234,275, 11,647,895) | 3.5E-04 (2.0E-04, 5.5E-04) | 198,969,798 (120,295,190, 284,395,855) | 9.5E-03 (5.7E-03, 1.4E-02) | 15,602,813 (8,878,154, 25,798,142) | 7.4E-04 (4.2E-04, 1.2E-03) |
| Suriname | 18,967,327 (14,200,078, 23,816,849) | 1.3E-03 (9.6E-04, 1.6E-03) | 7,709,004 (5,263,803, 10,588,572) | 5.2E-04 (3.6E-04, 7.2E-04) | 20,935,711 (15,642,681, 26,581,662) | 1.4E-03 (1.1E-03, 1.8E-03) | 2,793,320 (1,650,876, 3,872,318) | 1.9E-04 (1.1E-04, 2.6E-04) |
| Sweden | 3,856,164,365 (3,367,092,408, 4,316,201,129) | 6.6E-04 (5.8E-04, 7.4E-04) | 2,718,976,390 (2,389,011,704, 3,071,085,522) | 4.7E-04 (4.1E-04, 5.3E-04) | 4,006,153,321 (3,548,047,058, 4,469,835,731) | 6.9E-04 (6.1E-04, 7.7E-04) | 190,507,896 (158,406,697, 228,189,366) | 3.3E-05 (2.7E-05, 3.9E-05) |
| Switzerland | 3,485,829,366 (3,110,759,864, 3,852,629,681) | 4.7E-03 (4.2E-03, 5.2E-03) | 2,578,126,369 (2,258,171,792, 2,895,320,858) | 3.5E-03 (3.0E-03, 3.9E-03) | 4,159,113,544 (3,716,924,398, 4,589,778,611) | 5.6E-03 (5.0E-03, 6.2E-03) | 160,789,020 (130,615,587, 195,829,962) | 2.2E-04 (1.8E-04, 2.6E-04) |
| Syrian Arab Republic | 12,454,212 (9,039,987, 17,200,529) | 2.1E-05 (1.5E-05, 2.9E-05) | 2,359,471 (1,423,369, 3,511,984) | 4.0E-06 (2.4E-06, 5.9E-06) | 42,709,341 (30,858,265, 58,703,189) | 7.2E-05 (5.2E-05, 9.9E-05) | 162,228 (81,682, 232,825) | 2.7E-07 (1.4E-07, 3.9E-07) |
| Tajikistan | 27,091,372 (20,481,287, 35,169,305) | 8.0E-02 (6.0E-02, 1.0E-01) | 1,741,772 (1,149,803, 2,546,628) | 5.1E-03 (3.4E-03, 7.5E-03) | 50,408,842 (31,983,216, 80,246,419) | 1.5E-01 (9.4E-02, 2.4E-01) | 1,613,210 (1,001,974, 2,560,472) | 4.7E-03 (2.9E-03, 7.5E-03) |
| Thailand | 2,783,011,265 (2,108,792,039, 3,867,197,073) | 2.7E-04 (2.0E-04, 3.7E-04) | 494,523,185 (305,355,741, 932,664,652) | 4.7E-05 (2.9E-05, 9.0E-05) | 4,594,136,632 (2,733,506,232, 6,072,584,600) | 4.4E-04 (2.6E-04, 5.8E-04) | 103,166,204 (62,993,658, 208,566,380) | 9.9E-06 (6.0E-06, 2.0E-05) |
| Timor-Leste | 6,138,111 (4,619,519, 8,755,831) | 4.0E-03 (3.0E-03, 5.7E-03) | 515,643 (309,820, 1,016,606) | 3.4E-04 (2.0E-04, 6.7E-04) | 22,003,327 (16,283,251, 28,338,967) | 1.4E-02 (1.1E-02, 1.9E-02) | 875,665 (439,984, 2,063,608) | 5.7E-04 (2.9E-04, 1.4E-03) |
| Togo | 9,569,377 (6,718,584, 13,013,860) | 2.2E-06 (1.5E-06, 2.9E-06) | 140,603 (37,998, 248,794) | 3.2E-08 (8.5E-09, 5.6E-08) | 7,160,033 (3,209,800, 10,705,427) | 1.6E-06 (7.2E-07, 2.4E-06) | 564,265 (362,602, 847,162) | 1.3E-07 (8.2E-08, 1.9E-07) |
| Tonga | 957,589 (713,626, 1,305,933) | 9.5E-04 (7.1E-04, 1.3E-03) | 234,582 (158,774, 334,407) | 2.3E-04 (1.6E-04, 3.3E-04) | 748,170 (509,736, 1,033,742) | 7.4E-04 (5.1E-04, 1.0E-03) | 11,471 (6,175, 17,769) | 1.1E-05 (6.1E-06, 1.8E-05) |
| Trinidad and Tobago | 197,141,948 (150,577,168, 255,328,481) | 1.3E-03 (1.0E-03, 1.7E-03) | 123,172,278 (92,468,340, 159,375,903) | 8.3E-04 (6.2E-04, 1.1E-03) | 208,184,876 (160,179,829, 264,583,349) | 1.4E-03 (1.1E-03, 1.8E-03) | 15,625,434 (11,766,136, 20,214,535) | 1.1E-04 (7.9E-05, 1.4E-04) |
| Tunisia | 207,533,376 (147,733,897, 283,525,591) | 2.2E-03 (1.6E-03, 3.1E-03) | 62,028,217 (39,191,541, 90,180,784) | 6.7E-04 (4.2E-04, 9.7E-04) | 154,622,158 (108,065,109, 211,184,343) | 1.7E-03 (1.2E-03, 2.3E-03) | 43,573,852 (27,541,120, 67,966,566) | 4.7E-04 (3.0E-04, 7.3E-04) |
| Turkey | 5,797,830,005 (4,671,809,591, 7,331,717,876) | 2.9E-03 (2.3E-03, 3.6E-03) | 2,726,199,666 (1,986,666,666, 3,692,107,911) | 1.4E-03 (9.9E-04, 1.8E-03) | 9,501,846,728 (7,007,160,535, 11,728,587,040) | 4.7E-03 (3.5E-03, 5.8E-03) | 601,964,042 (414,966,252, 1,018,805,230) | 3.0E-04 (2.1E-04, 5.1E-04) |
| Tuvalu | 65,651 (50,832, 85,824) | 1.0E-03 (7.8E-04, 1.3E-03) | 7,303 (3,881, 10,920) | 1.1E-04 (6.0E-05, 1.7E-04) | 129,976 (88,420, 167,949) | 2.0E-03 (1.4E-03, 2.6E-03) | 2,650 (1,505, 3,835) | 4.1E-05 (2.3E-05, 5.9E-05) |
| Uganda | 160,736,917 (115,594,321, 224,841,218) | 1.2E-06 (8.7E-07, 1.7E-06) | 20,586,190 (13,703,950, 32,684,990) | 1.5E-07 (1.0E-07, 2.5E-07) | 58,072,597 (30,897,589, 91,784,846) | 4.4E-07 (2.3E-07, 6.9E-07) | 34,161,235 (19,497,407, 51,321,025) | 2.6E-07 (1.5E-07, 3.9E-07) |
| Ukraine | 1,640,437,772 (1,206,355,837, 2,134,559,300) | 6.3E-04 (4.6E-04, 8.2E-04) | 658,388,150 (473,692,299, 858,308,571) | 2.5E-04 (1.8E-04, 3.3E-04) | 2,453,245,364 (1,820,459,634, 3,170,089,315) | 9.4E-04 (7.0E-04, 1.2E-03) | 420,330,427 (302,174,062, 582,517,663) | 1.6E-04 (1.2E-04, 2.2E-04) |
| United Arab Emirates | 1,235,107,509 (955,379,829, 1,547,355,993) | 8.2E-04 (6.3E-04, 1.0E-03) | 596,609,580 (406,238,324, 865,261,597) | 4.0E-04 (2.7E-04, 5.7E-04) | 2,153,007,560 (1,494,312,602, 2,921,343,193) | 1.4E-03 (9.9E-04, 1.9E-03) | 219,255,430 (133,060,213, 361,876,392) | 1.5E-04 (8.8E-05, 2.4E-04) |
| United Kingdom | 23,694,658,419 (22,126,301,575, 24,750,915,441) | 1.1E-02 (1.0E-02, 1.1E-02) | 15,178,567,925 (14,132,462,144, 15,919,162,943) | 7.0E-03 (6.5E-03, 7.3E-03) | 23,129,408,939 (21,742,808,029, 24,035,832,945) | 1.1E-02 (1.0E-02, 1.1E-02) | 2,129,324,313 (2,030,178,350, 2,233,732,409) | 9.8E-04 (9.3E-04, 1.0E-03) |
| United Republic of Tanzania | 181,876,035 (135,461,037, 250,756,937) | 1.3E-06 (1.0E-06, 1.9E-06) | 21,717,933 (12,119,595, 32,796,188) | 1.6E-07 (9.0E-08, 2.4E-07) | 144,599,787 (86,400,326, 215,693,681) | 1.1E-06 (6.4E-07, 1.6E-06) | 39,571,680 (22,097,549, 61,630,228) | 2.9E-07 (1.6E-07, 4.6E-07) |
| United States of America | 154,696,000,000 (144,163,000,000, 162,496,000,000) | 7.2E-03 (6.7E-03, 7.6E-03) | 86,558,292,694 (80,759,720,961, 90,316,982,639) | 4.0E-03 (3.8E-03, 4.2E-03) | 166,564,000,000 (155,463,000,000, 172,867,000,000) | 7.8E-03 (7.3E-03, 8.1E-03) | 9,603,612,976 (9,098,868,682, 10,122,592,945) | 4.5E-04 (4.3E-04, 4.7E-04) |
| Uruguay | 652,495,441 (603,169,785, 705,107,224) | 3.7E-04 (3.5E-04, 4.0E-04) | 316,923,660 (290,203,956, 345,704,347) | 1.8E-04 (1.7E-04, 2.0E-04) | 680,275,291 (629,483,097, 735,972,198) | 3.9E-04 (3.6E-04, 4.2E-04) | 61,109,012 (50,781,701, 73,129,798) | 3.5E-05 (2.9E-05, 4.2E-05) |
| Uzbekistan | 145,710,042 (117,590,073, 178,276,771) | 2.2E-07 (1.8E-07, 2.7E-07) | 15,081,905 (11,307,048, 19,372,547) | 2.3E-08 (1.7E-08, 3.0E-08) | 395,603,209 (325,602,757, 481,498,064) | 6.1E-07 (5.0E-07, 7.4E-07) | 41,575,460 (30,480,706, 55,736,195) | 6.4E-08 (4.7E-08, 8.6E-08) |
| Vanuatu | 376,931 (288,456, 496,959) | 5.7E-06 (4.4E-06, 7.5E-06) | 32,826 (16,016, 52,666) | 5.0E-07 (2.4E-07, 8.0E-07) | 861,576 (562,543, 1,141,217) | 1.3E-05 (8.5E-06, 1.7E-05) | 20,093 (11,093, 29,328) | 3.0E-07 (1.7E-07, 4.4E-07) |
| Viet Nam | 1,419,931,835 (1,078,879,407, 1,958,710,658) | 2.8E-07 (2.1E-07, 3.8E-07) | 90,055,552 (61,334,285, 124,579,327) | 1.8E-08 (1.2E-08, 2.4E-08) | 1,296,486,104 (959,745,524, 1,747,395,999) | 2.5E-07 (1.9E-07, 3.4E-07) | 142,956,998 (92,716,248, 213,688,046) | 2.8E-08 (1.8E-08, 4.2E-08) |
| Yemen | 213,602,258 (145,049,013, 319,578,638) | 1.0E-03 (6.9E-04, 1.5E-03) | 29,475,599 (15,478,947, 51,628,842) | 1.4E-04 (7.4E-05, 2.5E-04) | 888,600,013 (484,644,551, 1,278,788,703) | 4.2E-03 (2.3E-03, 6.1E-03) | 76,311,940 (42,158,216, 118,065,211) | 3.6E-04 (2.0E-04, 5.6E-04) |
| Zambia | 70,088,717 (41,680,057, 130,478,900) | 4.8E-04 (2.8E-04, 8.9E-04) | 11,233,924 (4,347,047, 20,567,556) | 7.7E-05 (3.0E-05, 1.4E-04) | 43,356,442 (25,579,116, 63,879,584) | 3.0E-04 (1.7E-04, 4.4E-04) | 13,729,972 (7,994,904, 20,759,330) | 9.4E-05 (5.5E-05, 1.4E-04) |

Note: GDP, Gross Domestic Product

# Table S6 Estimated economic burden by country groups from 1990 to 2050

|  | High-income | Upper-middle-income | Lower-middle-income | Low-income |
| --- | --- | --- | --- | --- |
| 1990 | $858,000,000,000 ($855,000,000,000, $858,000,000,000) | $94,400,000,000 ($94,000,000,000, $95,600,000,000) | $41,000,000,000 ($40,200,000,000, $41,500,000,000) | $1,120,000,000 ($1,100,000,000, $1,170,000,000) |
| 2021 | $1,150,000,000,000 ($1,140,000,000,000,$1,150,000,000,000) | $288,000,000,000 ($284,000,000,000, $293,000,000,000) | $78,100,000,000 ($77,300,000,000, $79,300,000,000) | $3,480,000,000 ($3,430,000,000, $3,680,000,000) |
| 2030 | $589,000,000,000 ($569,000,000,000, $598,000,000,000) | $330,000,000,000 ($309,000,000,000, $357,000,000,000) | $112,000,000,000 ($101,000,000,000, $119,000,000,000) | $8,160,000,000 ($7,930,000,000, $9,220,000,000) |
| 2040 | $489,000,000,000 ($483,000,000,000, $511,000,000,000) | $461,000,000,000 ($455,000,000,000, $496,000,000,000) | $167,000,000,000 ($165,000,000,000, $176,000,000,000) | $18,600,000,000 ($17,200,000,000, $18,900,000,000) |
| 2050 | $410,000,000,000 ($305,000,000,000, $514,000,000,000) | $657,000,000,000 ($563,000,000,000, $726,000,000,000) | $260,000,000,000 ($236,000,000,000, $310,000,000,000) | $47,600,000,000 ($40,700,000,000, $52,300,000,000 |

# Table S7 Proportion of HM Economic Burden Attributed to Countries of Different Income Levels

|  | High-income | Upper-middle-income | Lower-middle-income | Low-income |
| --- | --- | --- | --- | --- |
| 1990 | 86.27% | 9.49% | 4.13% | 0.11% |
| 2021 | 75.60% | 19.02% | 5.15% | 0.23% |
| 2030 | 52.69% | 31.75% | 10.78% | 0.79% |
| 2040 | 43.07% | 40.61% | 14.67% | 1.64% |
| 2050 | 29.81% | 47.79% | 18.94% | 3.47% |

# Table S8 Decomposition of the Global Economic Burden of Hodgkin Lymphoma

| Factor Category | Contribution to Total Cost Change (%) |
| --- | --- |
| Total Cost Change | 100.00% |
| Change due to VSLY | -140.50% |
| Change due to DALYs | 240.50% |
| Population growth | 300.87% |
| Population aging | 74.48% |
| Epidemiological changes | -134.85% |

# Table S9 Decomposition of the Global Economic Burden of Non-Hodgkin Lymphoma

| Factor Category | Contribution to Total Cost Change (%) |
| --- | --- |
| Total Cost Change | 100.00% |
| Change due to VSLY | 51.31% |
| Change due to DALYs | 48.69% |
| Population growth | 60.91% |
| Population aging | 15.08% |
| Epidemiological changes | -27.30% |

# Table S10 Decomposition of the Global Economic Burden of Multiple Myeloma

| Factor Category | Contribution to Total Cost Change(%) |
| --- | --- |
| Total Cost Change | 100.00% |
| Change due to VSLY | 29.68% |
| Change due to DALYs | 70.32% |
| Population growth | 87.97% |
| Population aging | 21.78% |
| Epidemiological changes | -39.43% |

# Table S11 Decomposition of the Global Economic Burden of Leukemia

| Factor Category | Contribution to Total Cost Change(%) |
| --- | --- |
| Total Cost Change | 100.00% |
| Change due to VSLY | 108.12% |
| Change due to DALYs | -8.12% |
| Population growth | -10.16% |
| Population aging | -2.51% |
| Epidemiological changes | 4.55% |
